# The Polyketides with Antimicrobial Activities from a Mangrove Endophytic Fungus *Trichoderma lentiforme* ML-P8-2

**DOI:** 10.3390/md21110566

**Published:** 2023-10-28

**Authors:** Yihao Yin, Qi Tan, Jianying Wu, Tao Chen, Wencong Yang, Zhigang She, Bo Wang

**Affiliations:** School of Chemistry, Sun Yat-sen University, Guangzhou 510006, China; yinyh6@mail2.sysu.edu.cn (Y.Y.); tanq27@mail2.sysu.edu.cn (Q.T.); wujy89@mail2.sysu.edu.cn (J.W.); chent296@mail2.sysu.edu.cn (T.C.); yangwc6@mail2.sysu.edu.cn (W.Y.); cesshzhg@mail.sysu.edu.cn (Z.S.)

**Keywords:** mangrove endophytic fungus, *Trichoderma lentiforme*, polyketide, antimicrobial activity, AChE inhibitory activity

## Abstract

Five new polyketides, including two chromones (**1**–**2**), two phenyl derivatives (**4**–**5**), and a tandyukusin derivative (**6**), along with five known polyketides (**3** and **7**–**10**) were isolated from mangrove endophytic fungus *Trichoderma lentiforme* ML-P8-2. The planar structures of compounds were elucidated via detailed 1D, 2D NMR, and HR-ESI-MS analysis. ECD spectra, optical rotation values calculation, and alkali hydrolysis were applied in the determination of the absolute configuration of the new compounds. In bioassays, **6** and **9** exhibited promising antifungal activities against *Penicillium italicum*, with an MIC value of 6.25 μM for both compounds. Moreover, **3** displayed moderate AChE inhibitory activity with an IC_50_ value of 20.6 ± 0.3 μM.

## 1. Introduction

Mangrove endophytic fungi are an important source to provide biologically active lead compounds due to their unique living environment. Increasing numbers of secondary metabolites from mangrove-associated fungi have been newly reported in recent decades [1,2]. *Trichoderma* species have been widely discovered in marine environments, including soil, decaying wood, and living plants in mangrove forests [3,4]. Over 450 metabolites have been structurally documented from the species of *Trichoderma* genus [4,5], wherein polyketides are considered a significant group of these metabolites. Lai et al. reported the isolation of two new chromone polyketides with a broad spectrum of antimicrobial activities from *Trichoderma* sp. JWM29-10-1 [6]. Yamada et al. documented six decalin polyketides named tandyukusins from *Trichoderma harzianum* OUPS-111D-4, some of which exhibited significant cytotoxicities against human cancer cell lines [7,8,9]. Polyketides have attracted extensive attention due to their diverse chemical structures and wide range of biological activities [3,4,10].

**Figure 1:**
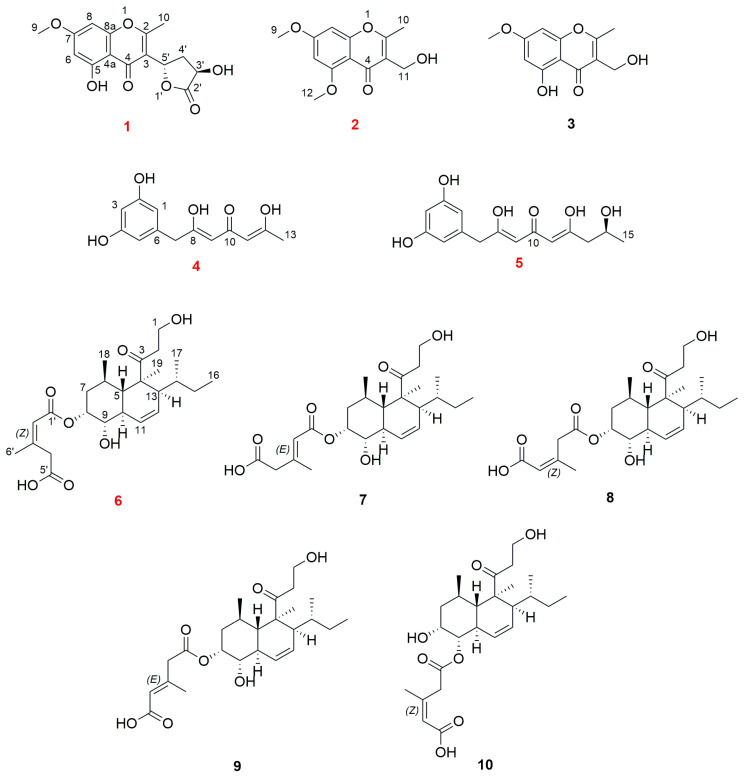
Structures of compounds **1**–**10**.

As part of our ongoing research for bioactive compounds from mangrove endophytic fungi [11,12,13], the study on chemical constituents of the ethyl acetate (EA) extract of the culture media of fungus *Trichoderma lentiforme* ML-P8-2 led to the isolation of ten polyketides (Figure 1), including five undescribed compounds (**1**–**2** and **4**–**6**) and five known compounds (**3** and **7**–**10**). The known compounds were identified as 5-hydroxy-3-(hydroxymethyl)-7-methoxy-2-methyl-4*H*-chromen-4-one (**3**) [14], trichoharzin (**7**) [15], tandyukisin D (**8**) [8], tandyukisin G (**9**) [6], and tandyukisin C (**10**) [8]. Antimicrobial, acetylcholinesterase (AChE) inhibitory, and cytotoxic activities of all isolated compounds were tested.

Herein, we report the detailed structural identification for the new compounds (**1**–**2** and **4**–**6**) and bioactivities results.

## 2. Results and Discussion

### 2.1. Structure Identification

**Figure 2:**
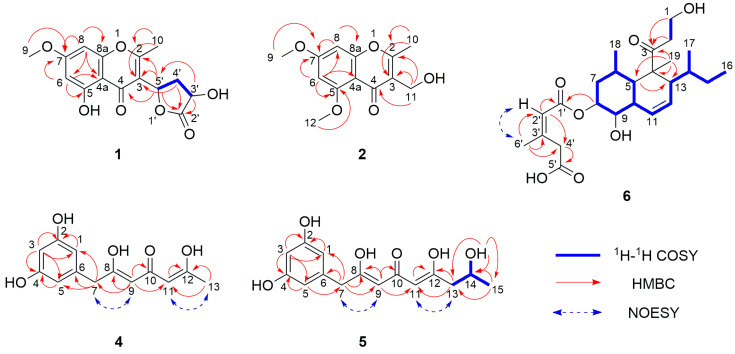
Two-dimensional NMR correlation signals of new compounds **1**, **2**, **4**–**6**.

Compound **1** was obtained as a light-yellow oil. Its molecular formula C_15_H_14_O_7_ with nine degrees of unsaturation was deduced by the ion peak of HR-ESI-MS *m*/*z* [M + Na]^+^ 329.0627 (calcd. for C_15_H_14_O_7_Na^+^, 329.0632). The ^1^H NMR spectrum showed two aromatic protons at *δ*_H_ 6.49 and 6.31, two oxymethines at *δ*_H_ 5.73 and 4.90, a methoxyl at *δ*_H_ 3.86, a methylene at *δ*_H_ 2.75 and 2.48, and a methyl at *δ*_H_ 2.52. The ^13^C NMR spectrum showed a conjugated carbonyl at *δ*_C_ 182.1, an ester carbonyl at *δ*_C_ 180.2, four oxygenated olefinic carbons at *δ*_C_ 168.0, 167.4, 163.2, and 159.0, two quaternary carbons at *δ*_C_ 117.8 and 105.1, two protonated olefinic carbons at *δ*_C_ 99.3 and 93.3, two oxymethines at *δ*_C_ 74.9 and 69.5, a methoxyl at *δ*_C_ 56.5, a methylene at *δ*_C_ 36.2, and a methyl at *δ*_C_ 18.1. The ^1^H–^1^H correlation spectroscopy (COSY) signal of H-6/H-8 and HMBC correlations from H-6 to C-4a, C-5, and C-7; H-8 to C-4a, C-7, and C-8a; H_3_-9 to C-7; and H_3_-10 to C-2 and C-3 revealed the presence of a 5-hydroxy-7-methoxy-2-methyl-4*H*-chromen-4-one moiety. Under the assistance of degrees of unsaturation, the ^1^H–^1^H COSY signals of H-3’/H-4’/H-5’ together with HMBC correlations from H-3’ to C-2’; H_2_-4’ to C-2’; H-5’ to C-2’ led to the identification of a 3’-hydroxy-2’-oxotetrahydrofuran moiety. And the above two moieties were connected via a C-3–C-5’ bond, according to HMBC correlations from H-5’ to C-2, C-3, C-4. Thus, the planar structure of **1** was established, as shown in Figure 2.

**Figure 3:**
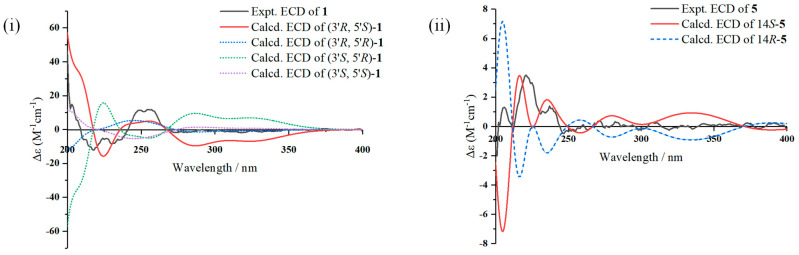
(**i**) Experimental and calculated ECD spectra of **1**; (**ii**) experimental and calculated ECD spectra of **5**.

The calculated ECD curves of all four possible configurations and the experimental ECD spectrum of **1** are shown in Figure 3. Only the ECD curve of (3’*R*, 5’*S*)-**1** was better matched with the experimental one, and the absolute configuration of **1** was assigned to be 3’*R*, 5’*S*. Finally, the structure of **1** was determined to be 5-hydroxy-3-((3’*R*, 5’*S*)-3’-hydroxy-2’-oxotetrahydrofuran-5’-yl)-7-methoxy-2-methyl-4*H*-chromen-4-one.

Compound **2** was isolated as a light-yellow oil. Its molecular formula C_13_H_14_O_5_ with seven degrees of unsaturation was deduced by the ion peak of HR-ESI-MS *m*/*z* [M + Na]^+^ 273.0734 (calcd. for C_13_H_14_O_5_Na^+^, 273.0733). The ^1^H NMR spectrum showed two aromatic protons at *δ*_H_ 6.59 and 6.50, an oxymethylene at *δ*_H_ 4.57, two methoxy signals at *δ*_H_ 3.91 and 3.90, and a methyl at *δ*_H_ 2.48. The ^13^C NMR spectrum showed a conjugated carbonyl at *δ*_C_ 178.2, four oxygenated olefinic carbons at *δ*_C_ 166.1, 165.5, 162.2, and 161.1, two quaternary carbons at *δ*_C_ 121.6 and 109.0, two protonated olefinic carbons at *δ*_C_ 97.1 and 93.9, two methoxys at *δ*_C_ 56.5 and 56.4, an oxymethylene at *δ*_C_ 55.5, and a methyl at *δ*_C_ 17.8. Comparing the 1D NMR data of **2** and **3**, **2** was highly similar to **3** [14] but possessed one more methoxyl. The 2D NMR data (Figure 2), especially the HMBC correlation signal from H_3_-12 to C-5, indicated that 5-OH was replaced by 5-OCH_3_, determining the structure of **2**, as 3-(hydroxymethyl)-5,7-dimethoxy-2-methyl-4*H*-chromen-4-one.

Compound **4** was acquired as a yellow oil. Its molecular formula C_13_H_14_O_5_ with seven degrees of unsaturation was deduced by the ion peak of HR-ESI-MS *m*/*z* [M + Na]^+^ 273.0734 (calcd. for C_13_H_14_O_5_Na^+^, 273.0733). The ^1^H NMR spectrum acquired in DMSO-*d*_6_ showed two exchangeable protons at *δ*_H_ 9.22, four aromatic protons at *δ*_H_ 6.12, 6.10, 6.07, and 6.04, a methylene adjacent to olefinic carbons at *δ*_H_ 3.65, and a methyl *δ*_H_ 2.20. The ^13^C NMR spectrum showed a conjugated carbonyl at *δ*_C_ 178.7, four oxygenated olefinic carbons at *δ*_C_ 167.6, 165.8, and 158.5 (2C), a quaternary carbon at *δ*_C_ 137.5, five protonated olefinic carbons at *δ*_C_ 113.2, 113.2, 106.9 (2C), and 101.2, a methylene adjacent to olefinic carbons at *δ*_C_ 38.7, and a methyl at *δ*_C_ 19.2. The HMBC correlation signals from H-3 to C-1, C-2, C-4, and C-5, and H-1 and H-5 to C-6 indicated the presence of a 2,4-dihydroxyphenyl moiety. HMBC correlations from H-9 to C-7, C-8, C-10, and C-11; H-11 to C-12; H_3_-13 to C-11 and C-12 constructed an 8,12-dihydroxyhepta-8,11-dien-10-one moiety. The two moieties connected with C-7 were determined via the analysis of HMBC correlations from H_2_-7 to C-1, C-5, and C-6. The NOESY correlations of H_2_-7/H-9 and H-11/H_3_-13 revealed that these protons were on the same side. Thus, the structure of **4** was established as (8*Z*,11*Z*)-7-(2,4-dihydroxyphenyl)-8,12-dihydroxyhepta-8,11-dien-10-one.

Compound **5** was isolated as a yellow oil. Its molecular formula C_15_H_18_O_6_ with seven degrees of unsaturation was deduced by the ion peak of HR-ESI-MS *m*/*z* [M − H_2_O + H]^+^ 277.1070 (calcd. for C_15_H_17_O_5_^+^, 277.1071). The ^1^H NMR spectrum acquired in DMSO-*d*_6_ showed three exchangeable protons at *δ*_H_ 9.22 (2H) and 4.80, four aromatic protons at *δ*_H_ 6.12, 6.10, 6.05, and 6.04, an oxymethine at *δ*_H_ 3.90, two methylenes at *δ*_H_ 3.64 and 2.52, and a methyl at *δ*_H_ 1.06. The ^13^C NMR spectrum showed a conjugated carbonyl at *δ*_C_ 178.7, four oxygenated olefinic carbons at *δ*_C_ 167.7, 166.9, and 158.5 (2C), a quaternary carbon at *δ*_C_ 137.5, five protonated olefinic carbons at *δ*_C_ 114.0, 113.3, 106.9 (2C), and 101.2, an oxymethine at *δ*_C_ 64.0, two methylenes at *δ*_C_ 42.6 and 38.8, and a methyl at *δ*_C_ 23.2. Compared with **4**, compound **5** was identified to possess an iso-propanol group instead of a methyl connecting to C-12, which was verified via the analyses of ^1^H–^1^H COSY signals H_2_-13/H-14/H_3_-15 and HMBC correlations from 14-OH to C-13, C-14, and C-15; H_2_-13 to C-11 and C-12. The NOESY correlations H_2_-7/H-9 and H-11/H_2_-13 revealed that these protons were on the same side. Therefore, the planar structure of **5** was established, as shown in Figure 2.

To identify the configuration of the only chiral center of **5**, C-14, experimental and calculated optical rotation values were obtained. The experimental optical rotation value ${\left[\alpha \right]}_{D}^{25}$ +23.5 (*c* 0.26, MeOH) was matched with the calculated one for 14*S*-**5**, ${\left[\alpha \right]}_{D}^{25}$ +29.0 (MeOH). Also, the calculated ECD spectrum of 14*S*-**5** showed a good agreement with the experimental one, as shown in Figure 3. Accordingly, the structure of **5** was determined to be (14*S*,8*Z*,11*Z*)-7-(2,4-dihydroxyphenyl)-8,12,14-trihydroxynona-8,11-dien-10-one.

Compound **6** was isolated as a pale-yellow oil. Its molecular formula C_25_H_38_O_7_ with seven degrees of unsaturation was deduced by the ion peak of HR-ESI-MS *m*/*z* [M + Na]^+^ 473.2509 (calcd. for C_25_H_38_O_7_Na^+^, 473.2510). Analyses of the ^1^H and ^13^C NMR data aided with HSQC revealed the presence of three carbonyls, two quaternary carbons, ten methines, five methylenes, and five methyls (NMR data in Table 1). A comparison with the NMR data of tandyukusin D (**8**) [8] indicated that **6** and **8** shared the same core structure, eujavanicol A [16], and the main difference occurred on the side chain. With the key HMBC from H-2’ to C-4’ and C-6’; H_2_-4’ to C-3’, C-5’, and C-6’; H_3_-6’ to C-2’, C-3’, and C-4’; and NOESY correlation H-2’/H_3_-6’, the side chain was established as a (2’*Z*)-3’-methyl-2’-pentenedioic acid moiety. The ^13^C resonance of C-6’ at *δ*_C_ 26.2 (larger than 20 ppm) additionally supported the *Z* configuration of the double bond on the side chain [6,17]. The HMBC from H-8 to C-1’ attached the side chain at C-8 of the eujavanicol A fragment. 

**Figure 4:**
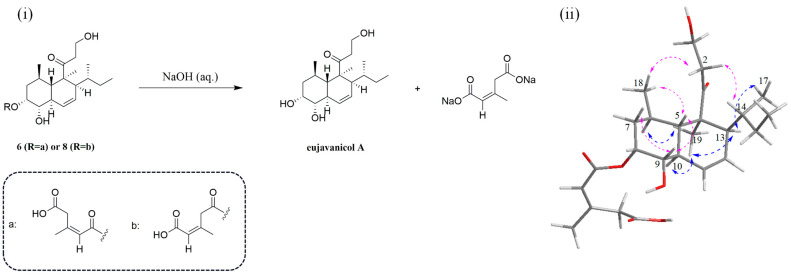
(**i**) alkali-hydrolysis reactions of compounds **6** and **8**; (**ii**) key NOESY correlation signals of **6**.

Further, the relative configuration of the eujavanicol A fragment was identified using the key NOESY correlations of H_3_-19 with H-6, H-10, and H-13 and of H-13 with H_3_-17, indicating these protons were on one face (Figure 4). The NOESY correlations of H-5 with H-9 and H_3_-18 suggested these protons were on the other face, and the decalin ring was *trans*. And ^1^H coupling constants *J*_7_*_α_*_,8_ = *J*_7_*_β_*_,8_ = *J*_9,8_ = 2.5 Hz showed that 9-OH was oriented *cis* to the esterified side chain at C-8 [7,8,9,15,16]. As for the configuration of C-14 at the sec-butyl group, the observed NOESY correlations in **6** were equal to those of **7**–**10** [6,8,15], which revealed that the rotation of the sec-butyl group in its pseudo-axial arrangement was restricted. Therefore, the relative configuration of C-14 was identified as R*. As a result, the relative configuration of **6** was determined as 4S*, 5S*, 6R*, 8R*, 9S*, 10R*, 13S*, and 14R*, in line with that in **7**–**10**. To determine the absolute configuration of **6**, treatments of **6** and tandyukusin D (**8**) with NaOH aqueous in MeOH were carried out, and the reactions resulted in the acquisition of eujavanicol A (Figure 4), which was identified according to its ^1^H, ^13^C NMR and HR-ESI-MS data (Figures S40–S42). Comparing the optical rotation values of products (eujavanicol A) from **6** and **8** with previous article [16] (respectively, for ${\left[\alpha \right]}_{D}^{25}$ +41.8, ${\left[\alpha \right]}_{D}^{25}$ +39.7, and ${\left[\alpha \right]}_{D}^{25}$ +49.9), it could be confirmed that the absolute configuration of **6** was 4*S*, 5*S*, 6*R*, 8*R*, 9*S*, 10*R*, 13*S*, and 14*R*. Finally, the structure of **6** was determined and named Tandyukisin J.

### 2.2. Antimicrobial Assays

The isolated compounds **1**–**10** along with the alkali hydrolysis treatment product eujavanicol A were evaluated for antibacterial activities against methicillin-resistant *Staphylococcus aureus* (MRSA), *Staphylococcus aureus*, *Bacillus subtilis*, *Salmonella typhimurium*, and *Pseudomonas aeruginosa* and for antifungal activities against *Candida albicans* and an agricultural plant pathogenic fungus *Penicillium italicum*. The results showed that the decalin derivatives (**6**–**10** and eujavanicol A) exhibited promising inhibitory activities against the fungi, with a minimal inhibition concentration (MIC) value in the range of 6.25 to 50 μM (Table 2), wherein **6** and **9** showed significant antifungal activities against *P. italicum*, with an MIC value of 6.25 μM for both compounds. And the chromone derivative **3** displayed moderate inhibitory activity against *C. albicans*, with an MIC value of 25 μM.

### 2.3. AChE Inhibitory Activity Assays

The isolated compounds **1**–**10**, along with the alkali hydrolysis treatment product eujavanicol A, were also evaluated for AChE inhibitory activities. The results showed that the chromone derivative **3** moderately inhibited AChE with IC_50_ values for 20.6 ± 0.3 μM, but the other tested compounds mostly exhibited weak inhibitory activities toward AChE (Table 3).

### 2.4. Cytotoxic Assays

The isolated compounds **1**–**10**, along with the alkali hydrolysis treatment product eujavanicol A, were also tested for cytotoxicities against six human cancer cell lines, which were MDA-MB-435, MDA-MB-231, HCT116, A549, SNB19, and PC3. But, only compound **1** exhibited a weak cytotoxicity against A549 with an IC_50_ value of 47.2 ± 5.5 μM, and the other compounds were inactive to the tested cell lines (IC_50_ > 50 μM).

## 3. Experimental Section

### 3.1. General Experimental Procedures

The 1D and 2D NMR spectra were obtained on a Bruker Advance 400 MHz spectrometer (Billerica, MA, USA) at room temperature. HR-ESI-MS spectra were acquired from a Thermo Fisher LTQ-Orbitrap-LCMS spectrometer (Palo Alto, Santa Clara, CA, USA). Optical rotation values were measured on an MCP500 modular polarimeter (Anton Paar, North Ryde, Austria) at 25 °C. UV-Vis and ECD curves were achieved on an Applied Photophysics Chirascan spectropolarimeter (Surrey, UK). Semi-preparative HPLC was utilized on an Ultimate 3000 separation module combined with a DAD detector produced by Thermo Fisher, and a ChiralPak AY-H column (5 μm, 4.6 × 250 mm) was applied for separation at 22 °C. Organic solvent was evaporated using a vacuum pump with a Heidolph rotavapor.

### 3.2. Fungal Material

The fungus *Trichoderma lentiforme* ML-P8-2 was isolated from a fresh leaf of the mangrove plant *Bruguiera gymnorrhiza*, which was collected in July 2022 from Dong Zhai Gang National Nature Reserve in Hainan Province, China. The fungus strain was identified according to sequencing of the internal transcribed spacer, and the results of a BLAST search on National Center for Biotechnology Information (NCBI) revealed it was most similar (99%) to the sequence of *Trichoderma lentiforme* (compared to MK714910.1). The sequence data have been uploaded and deposited at GenBank with accession No. OR617437. And the fungus specimen was kept in our laboratory at −20 °C.

### 3.3. Fermentation, Extraction, and Isolation

The fungus ML-P8-2 was proliferated in potato dextrose broth (PDB) in 4 × 500 mL Erlenmeyer flasks at 28 °C for 4 days in a shaker and then cultured in 150 × 1 L Erlenmeyer flasks, each containing 60 mL of 0.3% saline and 60 g of rice. After fermentation for 28 days, the culture media were soaked with MeOH and extracted with EA three times after concentration. Then, the extracts were condensed under 45 °C with a vacuum pump to finally obtain a crude extract (61 g). The crude extract was separated using a silica gel column, eluting with a gradient of petroleum ether (PE)/EA from 1:0 to 0:1 to afford 7 fractions (Frs. 1–7).

Frs. 7 (10.6 g) was subjected to Sephadex LH-20 [dichloromethane (DCM)/MeOH, *v*/*v*, 1:1] to yield five subfractions (SFrs. 7.1–7.5). SFrs. 7.3 (5.4 g) was applied to silica gel column chromatography (CC) (DCM/MeOH, *v*/*v*, 20:1) to give a mixture of **1**, **4**, and **5** (24 mg). The mixture was isolated using reverse phase C18 silica gel column (40−60 μm, MeOH/H_2_O, *v*/*v*, 8:2) to yield **1** (5.1 mg), **4** (2.3 mg), and **5** (4.5 mg). Frs. 3 (13.2 g) was subjected to Sephadex LH-20 (DCM/MeOH, *v*/*v*, 1:1) to yield three subfractions (SFrs. 3.1–3.3). SFrs. 3.3 (4.6 g) was applied to silica gel CC (DCM/MeOH, *v*/*v*, 200:1) to give mixture of **2** and **3** (26 mg). The mixture was isolated utilizing HPLC with the ChiralPak AY-H column, respectively, at *t*_R_ = 6.0 and 15.5 min (the gradient was *n*-hexane/2-propanol *v*/*v*, 9:1; flow rate: 1 mL/min) to give **2** (3.2 mg) and **3** (20.6 mg). Frs. 4 (15.5 g) was subjected to Sephadex LH-20 (DCM/MeOH, *v*/*v*, 1:1) to yield three subfractions (SFrs. 4.1–4.3). SFrs. 4.2 (6.0 g) was applied to silica gel CC (DCM/MeOH, *v*/*v*, 100:1) to give mixture of **6−10** (126 mg). The mixture was isolated utilizing HPLC with the ChiralPak AY-H column, respectively, at *t*_R_ = 15.0, 18.5, 9.0, 12.5, and 4.5 min (the gradient was *n*-hexane/2-propanol *v*/*v*, 85:15; flow rate: 1 mL/min) to give **6** (10.2 mg), **7** (35.7 mg), **8** (34.3 mg) **9** (12.3 mg), and **10** (6.5 mg).

5-hydroxy-3-((3’*R*, 5’*S*)-3’-hydroxy-2’-oxotetrahydrofuran-5’-yl)-7-methoxy-2-methyl-4*H*-chromen-4-one (**1**): C_15_H_14_O_7_; Light-yellow oil; ${\left[\alpha \right]}_{D}^{25}$ +20.5 (*c* 0.35, MeOH); UV (MeOH) *λ*_max_ (log *ε*): 206 (4.08), 249 (3.97), 258 (3.96), 291 (3.49) nm; HR-ESI-MS: *m*/*z* [M + Na]^+^ 329.0627 (calcd. for C_15_H_14_O_7_Na^+^, 329.0632); ^1^H and ^13^C NMR data (Table 4). Spectra in Figures S1–S8.

3-(hydroxymethyl)-5,7-dimethoxy-2-methyl-4*H*-chromen-4-one (**2**): C_13_H_14_O_5_; Light-yellow oil; UV (MeOH) *λ*_max_ (log *ε*): 201 (3.81), 230 (3.98), 280 (3.51) nm; HR-ESI-MS: *m*/*z* [M + Na]^+^ 273.0734 (calcd. for C_13_H_14_O_5_Na^+^, 273.0733); ^1^H and ^13^C NMR data (Table 4). Spectra in Figures S9–S15.

(8*Z*,11*Z*)-7-(2,4-dihydroxyphenyl)-8,12-dihydroxyhepta-8,11-dien-10-one (**4**): C_13_H_14_O_5_; Yellow oil; UV (MeOH) *λ*_max_ (log *ε*): 201 (4.12), 241 (3.74), 276 (3.04) nm; HR-ESI-MS: *m*/*z* [M + Na]^+^ 273.0734 (calcd. for C_13_H_14_O_5_Na^+^, 273.0733); ^1^H and ^13^C NMR data (Table 5). Spectra in Figures S16–S23.

(14*S*,8*Z*,11*Z*)-7-(2,4-dihydroxyphenyl)-8,12,14-trihydroxynona-8,11-dien-10-one (**5**): C_15_H_18_O_6_; Yellow oil; ${\left[\alpha \right]}_{D}^{25}$ +23.5 (*c* 0.26, MeOH); UV (MeOH) *λ*_max_ (log *ε*): 201 (4.18), 246 (3.76), 277 (3.02) nm; HR-ESI-MS: *m*/*z* [M − H_2_O + H]^+^ 277.1070 (calcd. for C_15_H_17_O_5_^+^, 277.1071); ^1^H and ^13^C NMR data (Table 5). Spectra in Figures S24–S31.

Tandyukisin J (**6**): C_25_H_38_O_7_; Pale-yellow oil; ${\left[\alpha \right]}_{D}^{25}$ +17.6 (*c* 0.45, MeOH); UV (MeOH) *λ*_max_ (log *ε*): 215 (3.82) nm; HR-ESI-MS: *m*/*z* [M + Na]^+^ 473.2509 (calcd. for C_25_H_38_O_7_Na^+^, 473.2510); ^1^H and ^13^C NMR data (Table 1). Spectra in Figures S32–S39.

### 3.4. Alkali-hydrolysis Treatments for Compounds 6 and 8

Compound **6** (3.0 mg, 6.7 μmol) in MeOH (200 μL) was stirred evenly for 5 min, and then 200 μL of aqueous NaOH (1.0 M) was added. The reaction mixture was stirred for 30 min at room temperature. After completion, the reaction mixture was extracted with EA thrice, and the organic layer was concentrated under reduced pressure to afford eujavanicol A (1.9 mg). In order to determine its absolute configuration and exclude potential differences in test conditions with the previous articles, the known compound **8** (5.0 mg) was also hydrolyzed to produce eujavanicol A (3.2 mg) following the same procedure.

### 3.5. ECD and Optical Rotation Computation Methods

Initial conformational analysis was carried out using the Merck molecular force field method with the Spartan 14’ software (Wavefunction Inc., Irvine, CA, USA). The conformation with a Boltzmann population larger than 1% was selected for optimization and calculation in MeOH at B3LYP/6-31+G(d,p) level with the density functional theory (DFT) executed via Gaussian 09 [18]. The ECD spectra and optical rotation values were extracted and generated via the SpecDis 1.6 software (University of Würzburg, Würzburg, Germany). The Gibbs free energy, Boltzmann population and Cartesian coordinates for low-energy conformers of **1** and **5** for calculation in Tables S1–S6.

### 3.6. Antimicrobial Assays

The compounds to be tested were dissolved individually in dimethyl sulfoxide (DMSO), and the antimicrobial activity assays were performed in 96-well plates via a serial dilution test in the range of 0.1–100 μM, according to the methods previously reported [11,19]. All measurements were conducted in triplicate. Ampicillin and ketoconazole were applied as positive controls for antibacterial and antifungal assays, respectively, and DMSO was utilized as a blank control. 

### 3.7. AChE Inhibition Assays

Compounds **1**–**10** and eujavanicol A were evaluated for AChE inhibitory activity, following the same method previously described [12]. Donepezil hydrochloride was taken as a positive control. All measurements were conducted in triplicate from two independent experiments. The reported IC_50_ was the average value of two independent experiments.

### 3.8. Cytotoxic Assays

The cytotoxicities of compounds **1**–**10** and eujavanicol A on cells were assessed using MTT assay as described previously [13]. Six cell lines were used, MDA-MB-435 (breast cancer cells), MDA-MB-231 (breast cancer cells), HCT116 (colon cancer cells), A549 (lung cancer cells), SNB19 (glioma cells), and PC3 (prostate cancer cells), which were acquired from Fu Erbo Biotechnology Co., Ltd. (Guangzhou, China).

## 4. Conclusions

In summary, five new polyketides, including two chromones (**1**–**2**), two phenyl derivatives (**4**–**5**), and a tandyukusin derivative (**6**), along with five known polyketides (**3** and **7**–**10**), were isolated from mangrove endophytic fungus *Trichoderma lentiforme* ML-P8-2, and it is the first time to report secondary metabolites from this specific species. The planar structures of the isolated compounds were elucidated via detailed 1D, 2D NMR, and HR-ESI-MS analysis. ECD spectra, optical rotation values calculation, and alkali hydrolysis were applied in the determination of the absolute configuration of the new compounds. In bioassays, antimicrobial, AChE inhibitory, and cytotoxic activities tests for compounds **1**–**10**, together with the alkali-hydrolysis treatment product eujavanicol A, were carried out. Compounds **6** and **9** exhibited promising antifungal activities against *Penicillium italicum*, with MIC, both for 6.25 μM. Moreover, **3** displayed moderate AChE inhibitory activity with an IC_50_ value of 20.6 ± 0.3 μM.

## Figures and Tables

**Table 1:** ^1^H and ^13^C NMR data of **6** (acquired in CDCl_3_).

Position	6		Position	6	
	*δ*_C_, Type	*δ*_H_ (*J* in Hz)		*δ*_C_, Type	*δ*_H_ (*J* in Hz)
1	58.1, CH_2_	3.85, ddd (11.0, 6.0, 3.8) 3.93, ddd (11.0, 7.2, 3.4)	14	37.3, CH	1.14, m
2	41.3, CH_2_	2.69, ddd (18.9, 6.0, 3.4) 2.88, ddd (18.9, 7.2, 3.8)	15	24.6, CH_2_	0.75, overlap 1.48, d (7.0)
3	215.7, C		16	12.7, CH_3_	0.77, d (4.2)
4	52.6, C		17	19.4, CH_3_	0.95, d (6.7)
5	43.1, CH	1.97, d (4.1)	18	22.4, CH_3_	0.60, d (5.8)
6	31.6, CH	1.62, overlap	19	19.5, CH_3_	1.28, s
7	39.1, CH_2_	1.59, overlap 1.89, dd (12.1, 2.8)	1’	167.5, C	
8	73.6, CH	5.30, m	2’	119.6, CH	6.01, s
9	74.4, CH	3.58, dd (10.9, 3.2)	3’	151.9, C	
10	40.4, CH	2.12, d (10.9)	4’	39.8, CH_2_	3.63, d (14.6) 3.77, d (14.6)
11	125.7, CH	6.05, d (10.6)	5’	172.8, C	
12	124.1, CH	5.72, ddd (10.6, 4.7, 2.6)	6’	26.2, CH_3_	2.06, s
13	52.5, CH	1.97, d (4.7)			

**Table 2:** MIC for antibacterial and antifungal activities of compounds **1**–**10** and eujavanicol A.

	MIC of Compounds/μM												
	1	2	3	4	5	6	7	8	9	10	Euj. A ^1^	Amp. ^2^	Ket. ^3^
MRSA	>100	>100	>100	>100	>100	>100	>100	>100	>100	>100	>100	0.25	NT ^4^
*S. aureus*	>100	>100	>100	>100	>100	>100	>100	>100	50	>100	>100	0.25	NT
*B. subtilis*	>100	>100	>100	>100	>100	>100	>100	>100	>100	>100	>100	0.25	NT
*S. typhimurium*	>100	>100	>100	>100	>100	>100	>100	50	>100	>100	>100	0.25	NT
*P. aeruginosa*	>100	>100	>100	>100	>100	>100	>100	50	50	>100	>100	0.13	NT
*C. albicans*	50	100	25	>100	>100	25	50	50	25	25	25	NT	0.13
*P. italicum*	>100	>100	>100	>100	>100	6.25	12.5	12.5	6.25	50	25	NT	1.56

^1^ Eujavanicol A; ^2^ Ampicillin, positive control toward bacteria; ^3^ Ketoconazole, positive control toward fungi; ^4^ Not tested.

**Table 3:** IC_50_ for AChE inhibitory activities of compounds **1**–**10** and eujavanicol A.

Compounds	IC_50_/μM	Compounds	IC_50_/μM
**1**	38.6 ± 0.2	**7**	38.3 ± 0.4
**2**	33.7 ± 0.4	**8**	77.9 ± 1.7
**3**	20.6 ± 0.3	**9**	43.6 ± 0.4
**4**	37.7 ± 0.6	**10**	50.9 ± 0.5
**5**	51.3 ± 0.5	Eujavanicol A	32.4 ± 0.7
**6**	40.2 ± 0.7	Donepezil Hydrochloride ^1^	65.5 ± 1.5 (nM)

^1^ Positive control.

**Table 4:** ^1^H and ^13^C NMR data of **1** and **2** (acquired in CD_3_OD).

Position	1		2	
	*δ*_C_, Type	*δ*_H_ (*J* in Hz)	*δ*_C_, Type	*δ*_H_ (*J* in Hz)
2	168.0, C		165.5, C	
3	117.8, C		121.6, C	
4	182.1, C		178.2, C	
4a	105.1, C		109.0, C	
5	163.2, C		162.2, C	
6	99.3, CH	6.32, d (2.2)	97.1, CH	6.50, d (2.3)
7	167.4, C		166.1, C	
8	93.3, CH	6.49, d (2.2)	93.9, CH	6.59, d (2.3)
8a	159.0, C		161.1, C	
9	56.5, CH_3_	3.86, s	56.4, CH_3_	3.91, s
10	18.1, CH_3_	2.52, s	17.8, CH_3_	2.48, s
11			55.5, CH_2_	4.57, s
12			56.5, CH_3_	3.90, s
2′	180.2, C			
3′	69.5, CH	4.90, dd (9.3, 5.9)		
4′	36.2, CH_2_	2.48, m 2.75, ddd (13.5, 9.3, 4.1)		
5′	74.9, CH	5.73, dd (9.9, 4.1)		

**Table 5:** ^1^H and ^13^C NMR data of **4** and **5** (acquired in DMSO-*d*_6_).

Position	4		5	
	*δ*_C_, Type	*δ*_H_ (*J* in Hz)	*δ*_C_, Type	*δ*_H_ (*J* in Hz)
1	106.9, CH	6.12, d (1.9)	106.9, CH	6.12, d (2.1)
2	158.5, C		158.5, C	
3	101.2, CH	6.10, d (1.9)	101.2, CH	6.10, d (2.1)
4	158.5, C		158.5, C	
5	106.9, CH	6.12, d (1.9)	106.9, CH	6.12, d (2.1)
6	137.5, C		137.5, C	
7	38.7, CH_2_	3.65, s	38.8, CH_2_	3.64, s
8	167.6, C		167.7, C	
9	113.2, CH	6.04, d (2.3)	113.3, CH	6.04, d (2.3)
10	178.7, C		178.7, C	
11	113.2, CH	6.07, dd (2.3, 0.9)	114.0, CH	6.05, d (2.3)
12	165.8, C		166.9, C	
13	19.2, CH_3_	2.20, d (0.9)	42.6, CH_2_	2.52, d (3.7)
14			64.0, CH	3.90, m
15			23.2, CH_3_	1.06, d (6.1)
2-OH		9.22, s		9.22, s
4-OH		9.22, s		9.22, s
14-OH				4.80, d (5.1)

## Data Availability

Data are contained within this article and Supplementary Materials.

## Supplementary Materials

The following supporting information can be downloaded at: https://www.mdpi.com/article/10.3390/md21110566/s1, the 1D, 2D NMR, HR-ESI-MS, and UV-vis spectra of compound **1** (Figures S1–S8), compound **2** (Figures S9–S15), compound **4** (Figures S16–S23), compound **5** (Figures S24–S31), and compound **6** (Figures S32–S39); the 1D, 2D NMR, and HR-ESI-MS spectra of the alkali hydrolysis treatment product eujavanicol A (Figures S40–S42); the Gibbs free energy, Boltzmann population and Cartesian coordinates for low-energy conformers of **1** and **5** for calculation (Tables S1–S6).
