# Supplementary material for: The Polyketides with Antimicrobial Activities from a Mangrove Endophytic Fungus Trichoderma lentiforme ML-P8-2
Source: Mar Drugs. 2023 Oct 28;21(11):566. doi: 10.3390/md21110566 (PMC10672575; doi:10.3390/md21110566)
Supplement: Supplementary file 1 [file marinedrugs-21-00566-s001.zip › marinedrugs-2692587-supplementary.pdf]

Supplementary Materials

The Polyketides with Antimicrobial Activities from a Mangrove Endophytic Fungus *Trichoderma lentiforme* ML-P8-2

Yihao Yin, Qi Tan, Jianying Wu, Tao Chen, Wencong Yang, Zhigang She, and Bo Wang *

School of Chemistry, Sun Yat-sen University, Guangzhou 510006, China; yinyh6@mail2.sysu.edu.cn (Y.Y.); tanq27@mail2.sysu.edu.cn (Q.T.); wujy89@mail2.sysu.edu.cn (J.W.); chent296@mail2.sysu.edu.cn (T.C.); yangwc6@mail2.sysu.edu.cn (W.Y.); cesshzhg@mail.sysu.edu.cn (Z.S.)

* Correspondence: ceswb@mail.sysu.edu.cn

Abstract: Five new polyketides, including two chromones (**1** – **2**), two phenyl derivatives (**4** – **5**), and a tandyukusin derivative (**6**), along with five known polyketides (**3** and **7** – **10**) were isolated from mangrove endophytic fungus *Trichoderma lentiforme* ML-P8-2. The planar structures of compounds were elucidated through detailed 1D, 2D NMR and HR-ESI-MS analysis. ECD spectra, optical rotation values calculation and alkali-hydrolysis were applied in the determination of the absolute configuration of the new compounds. In bioassays, **6** and **9** exhibited promising antifungal activities against *Penicillium italicum*, with MIC both for 6.25 μM. Besides, **3** displayed moderate AChE inhibitory activity with IC_{50} for 20.6 ± 0.3 μM.

Keywords: mangrove endophytic fungus; *Trichoderma lentiforme*; polyketide; antimicrobial activity; AChE inhibitory activity

List of Contents

Figure S1. ^{1}H NMR spectrum of **1** in CD_3OD. S1

Figure S2. ^{13}C NMR spectrum of **1** in CD_3OD. S1

Figure S3. HSQC spectrum of **1**........ S2

Figure S4. ^{1}H-^{1}H COSY spectrum of **1**. S2

Figure S5. HMBC spectrum of **1**........ S3

Figure S6. NOESY spectrum of **1**. S3

Figure S7. HR-ESI-MS spectrum of **1**. S4

Figure S8. UV-vis spectrum of **1**........ S4

Figure S9. ^{1}H NMR spectrum of **2** in CD_3OD. S5

Figure S10. ^{13}C NMR spectrum of **2** in CD_3OD. S5

Figure S11. HSQC spectrum of **2**........ S6

Figure S12. ^{1}H-^{1}H COSY spectrum of **2**. S6

Figure S13. HMBC spectrum of **2**........ S7

Figure S14. HR-ESI-MS spectrum of **2**. S7

Figure S15. UV-vis spectrum of **2**........ S8

Figure S16. ^{1}H NMR spectrum of **4** in DMSO-d_6........ S8

Figure S17. ^{13}C NMR spectrum of **4** in DMSO-d_6. .... S9

Figure S18. HSQC spectrum of **4**........ S9

Figure S19. ^{1}H-^{1}H COSY spectrum of **4**. S10

Figure S20. HMBC spectrum of **4**........ S10

Figure S21. NOESY spectrum of **4**. S11

Figure S22. HR-ESI-MS spectrum of **4**. S11

Figure S23. UV-vis spectrum of **4**........ S12

Figure S24. ^{1}H NMR spectrum of **5** in DMSO-d_6........ S12

Figure S25. ^{13}C NMR spectrum of **5** in DMSO-d_6. .... S13

Figure S26. HSQC spectrum of **5**........ S13

Figure S27. ^{1}H-^{1}H COSY spectrum of **5**. S14

Figure S28. HMBC spectrum of **5**........ S14

Figure S29. NOESY spectrum of **5**. S15

Figure S30. HR-ESI-MS spectrum of **5**. S15

Figure S31. UV-vis spectrum of **5**..S16

Figure S32. ^{1}H NMR spectrum of **6** in $CDCl_3$. S16

Figure S33. ^{13}C NMR spectrum of **6** in $CDCl_3$. S17

Figure S34. HSQC spectrum of **6**..S17

Figure S35. ^{1}H-^{1}H COSY spectrum of **6**. S18

Figure S36. HMBC spectrum of **6**..S18

Figure S37. NOESY spectrum of **6**. S19

Figure S38. HR-ESI-MS spectrum of **6**. S19

Figure S39. UV-vis spectrum of **6**..S20

Figure S40. ^{1}H NMR spectrum of eujavanicol A in $CDCl_3$. S20

Figure S41. ^{13}C NMR spectrum of eujavanicol A in $CDCl_3$. S21

Figure S42. HR-ESI-MS spectrum of eujavanicol A. S21

Table S1. Gibbs free energy and Boltzmann population of low energy conformers of (3′*R*, 5′*S*)-**1** in MeOH. S22

Table S2. Gibbs free energy and Boltzmann population of low energy conformers of (3′*R*, 5′*R*)-**1** in MeOH. S22

Table S3. Gibbs free energy and Boltzmann population of low energy conformers of 14*S*-**5** in MeOH. 22

Table S4. Cartesian coordinates for the low-energy optimized conformers of (3′*R*, 5′*S*)-**1** at B3LYP/6–31+g (d,p) level of theory in MeOH. S23

Table S5. Cartesian coordinates for the low-energy optimized conformers of (3′*R*, 5′*R*)-**1** at B3LYP/6–31+g (d,p) level of theory in MeOH. S30

Table S6. Cartesian coordinates for the low-energy optimized conformers of 14*S*-**5** at B3LYP/6–31+g (d,p) level of theory in MeOH. S32

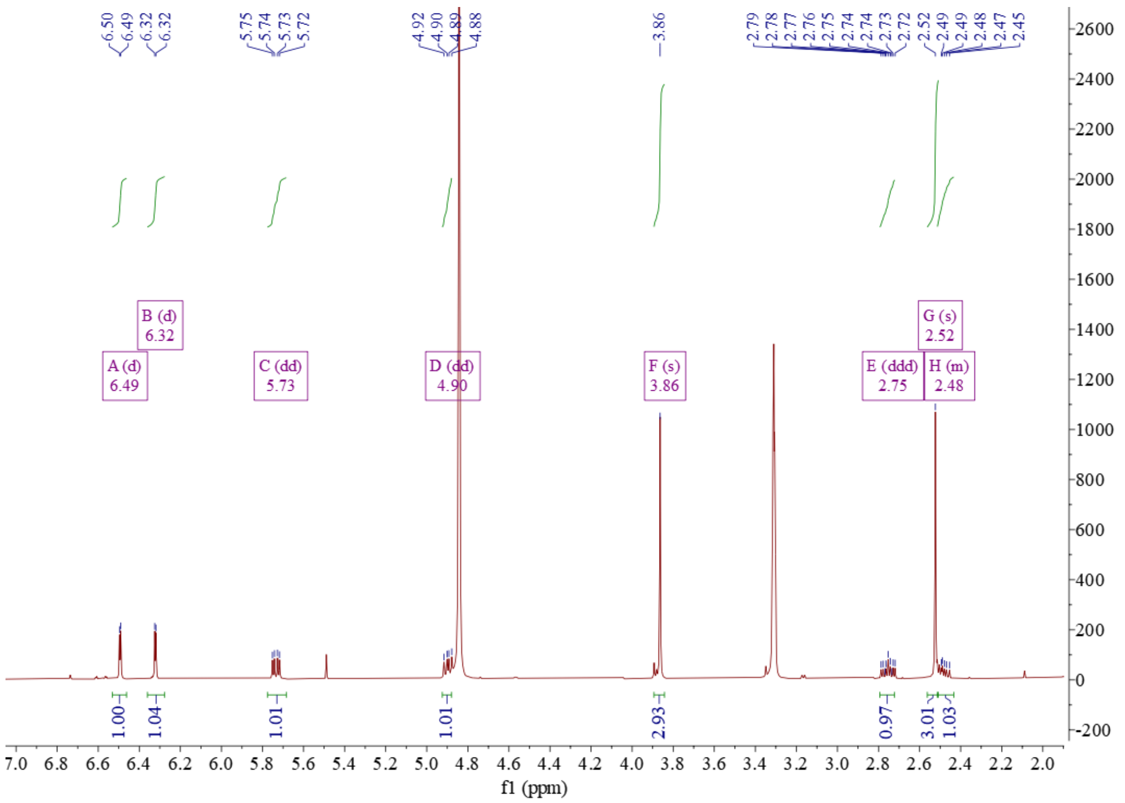

Figure S1. ^{1}H NMR spectrum of **1** in CD_3OD.

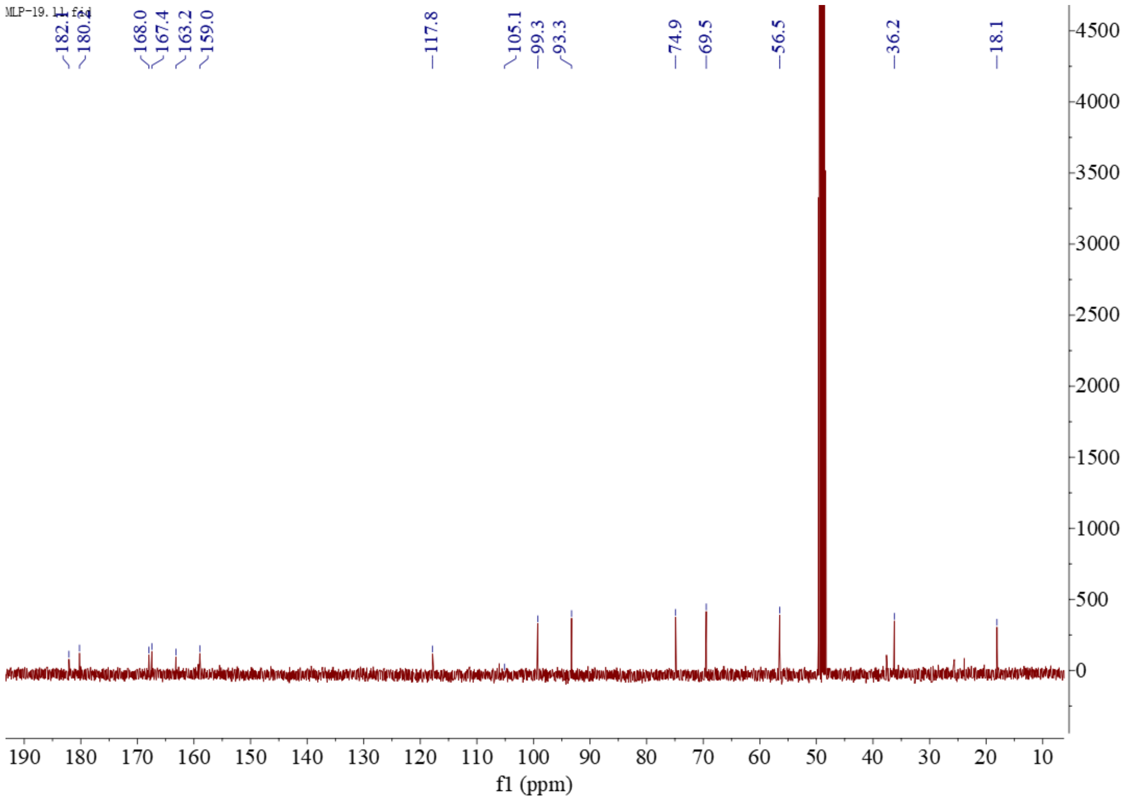

Figure S2. ^{13}C NMR spectrum of **1** in CD_3OD.

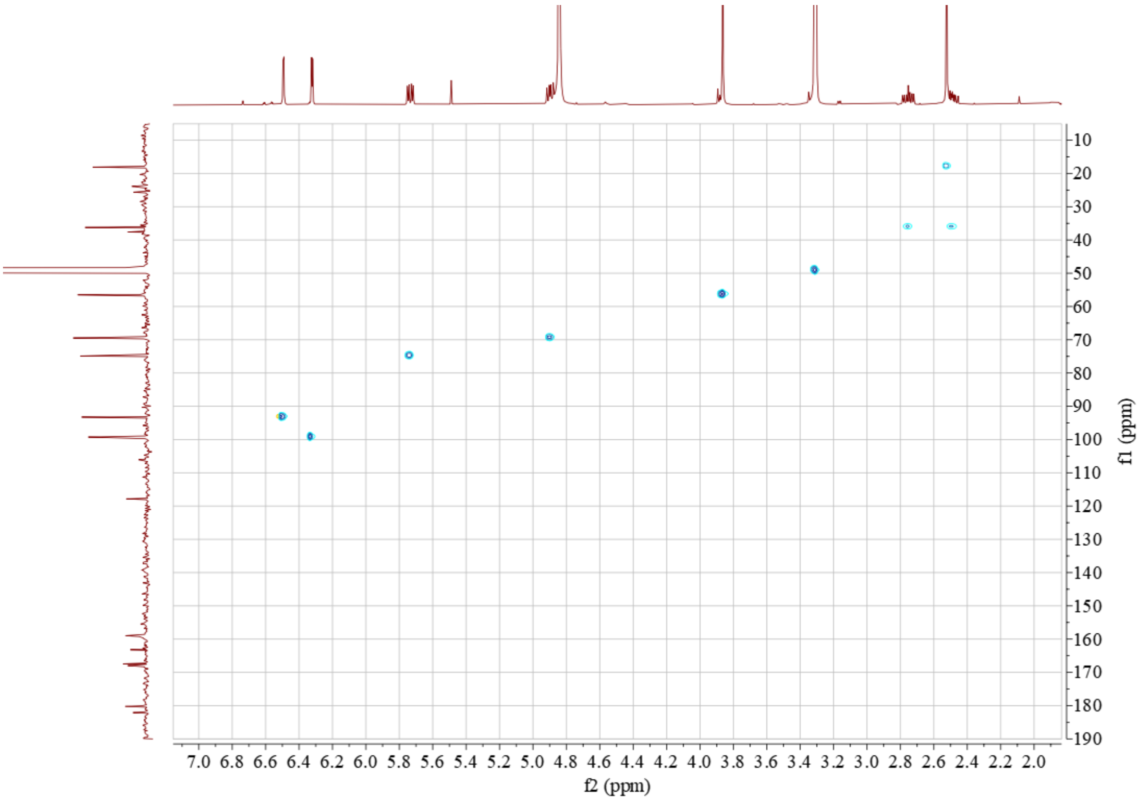

Figure S3. HSQC spectrum of **1**.

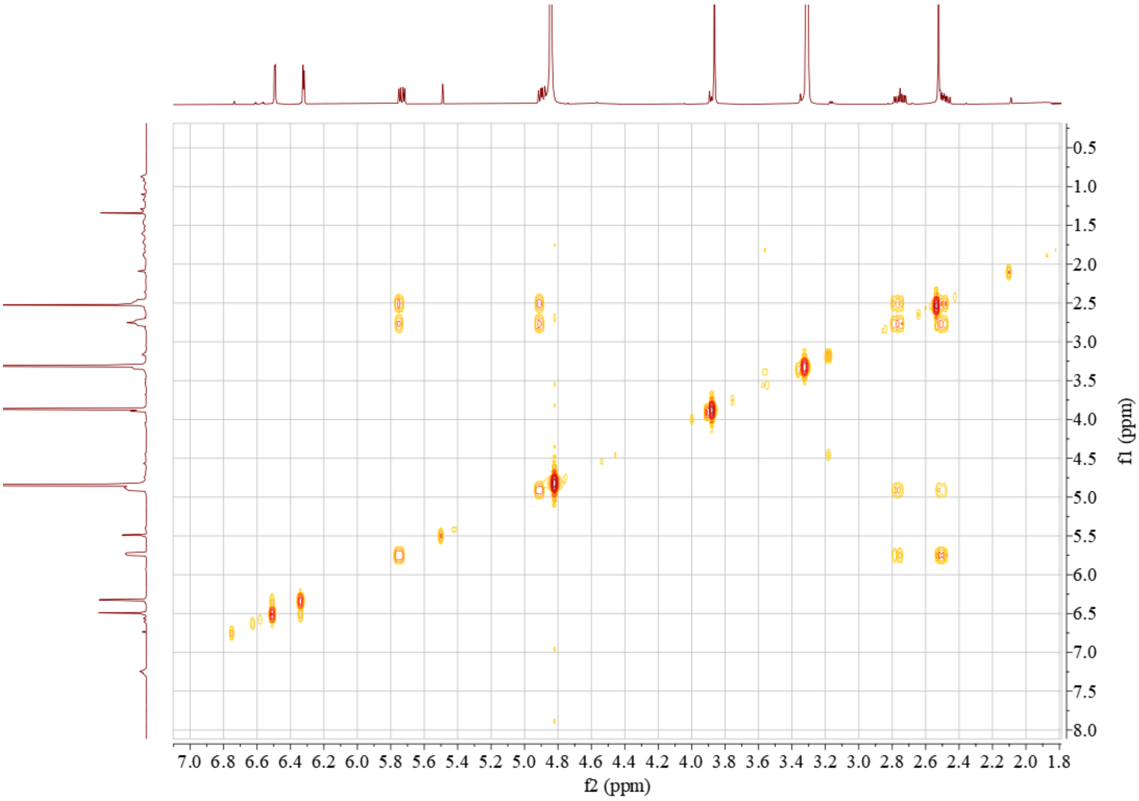

Figure S4. ^1H-^1H COSY spectrum of **1**.

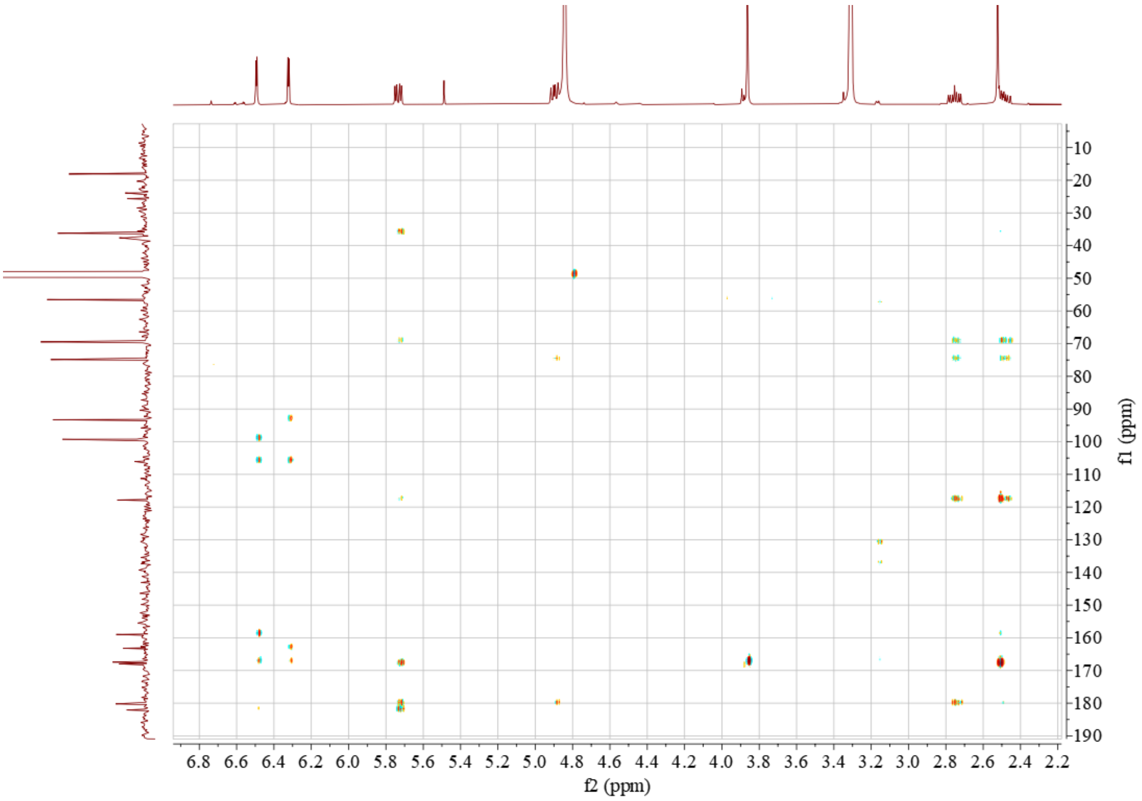

Figure S5. HMBC spectrum of **1**.

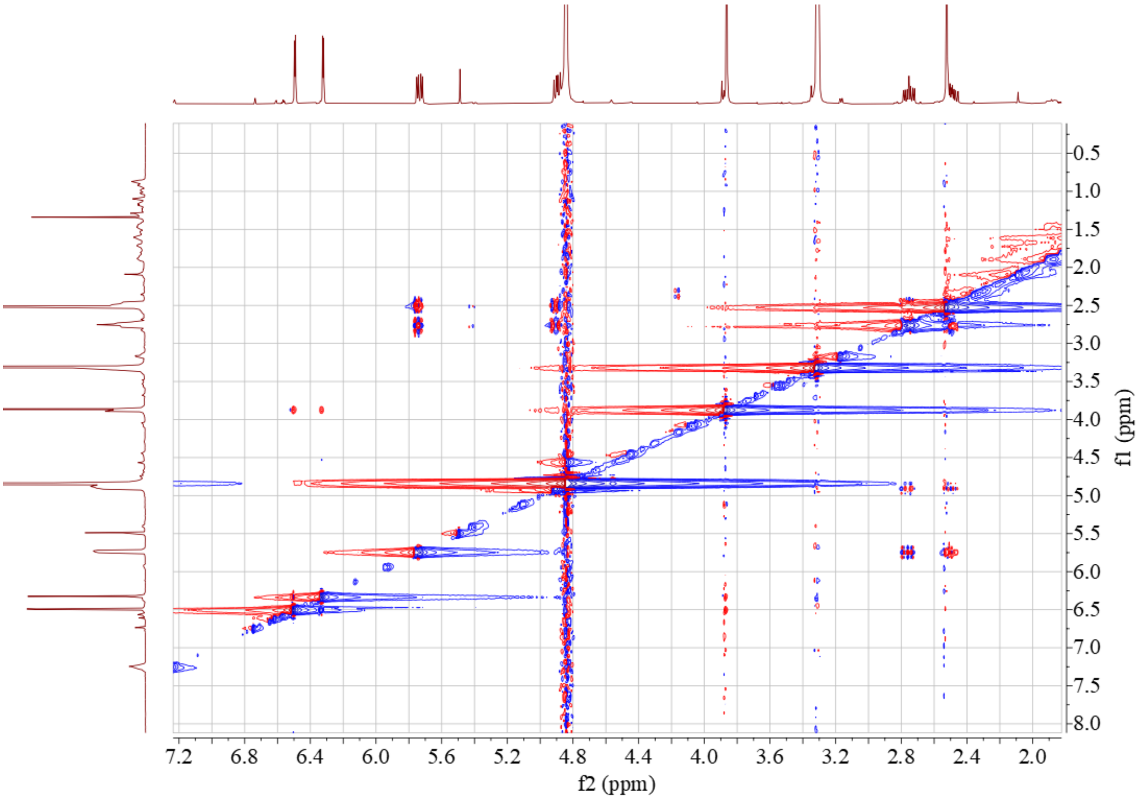

Figure S6. NOESY spectrum of **1**.

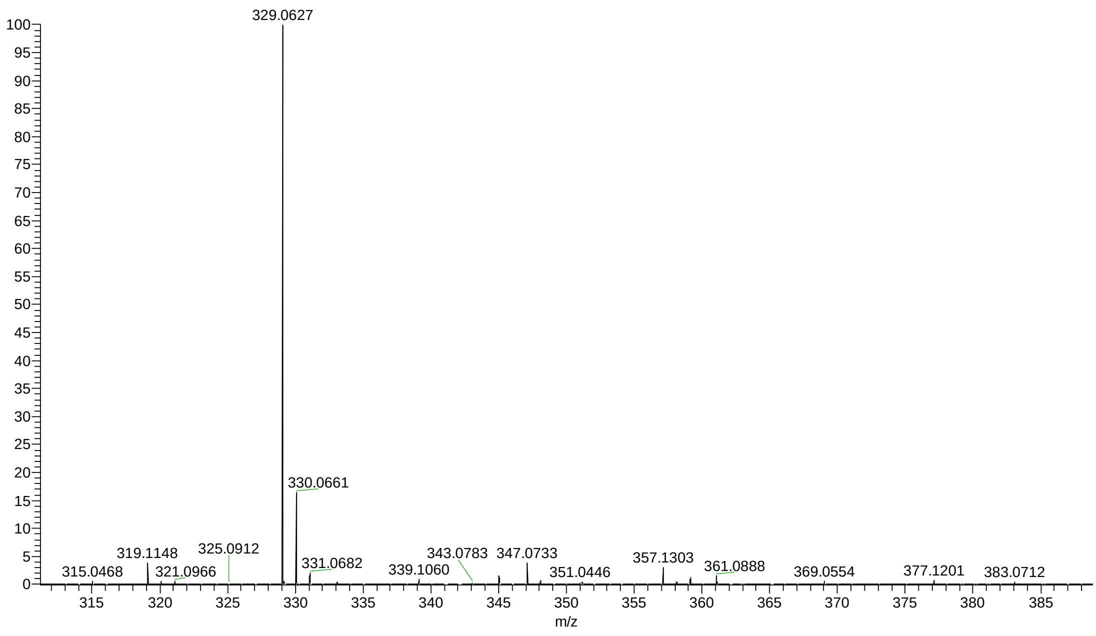

Figure S7. HR-ESI-MS spectrum of **1**.

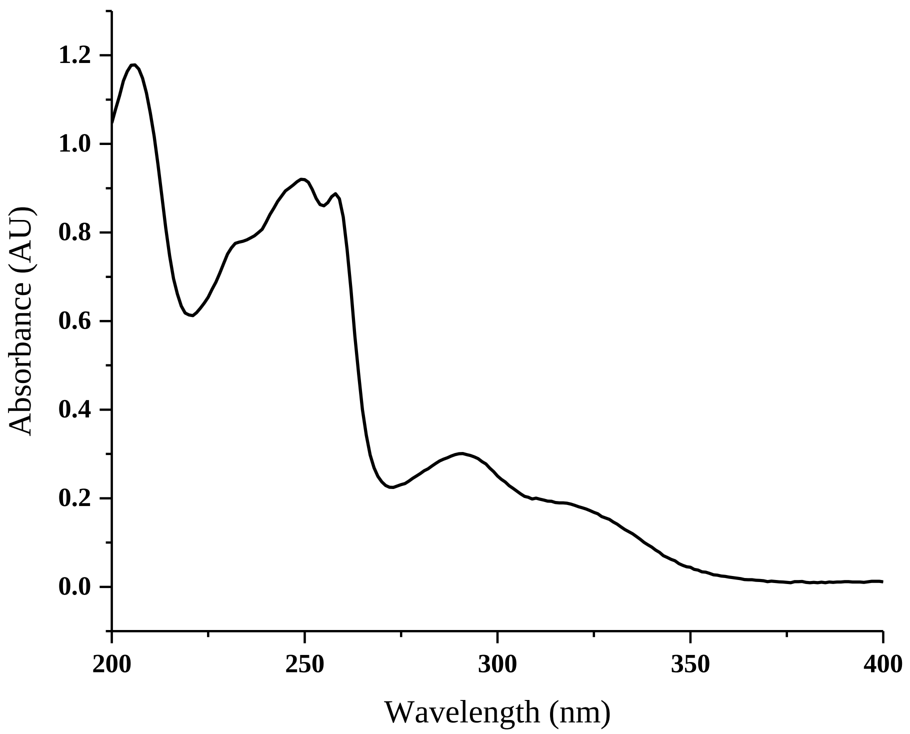

Figure S8. UV-vis spectrum of **1**.

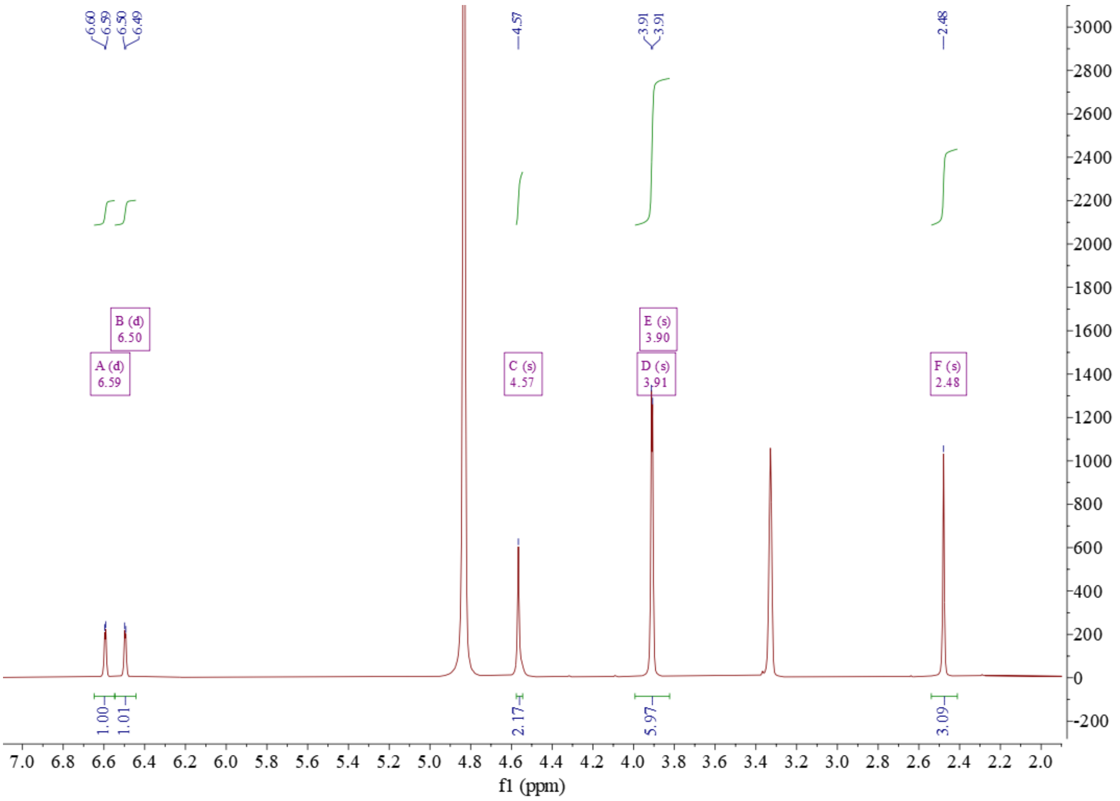

Figure S9. ^{1}H NMR spectrum of **2** in CD_3OD.

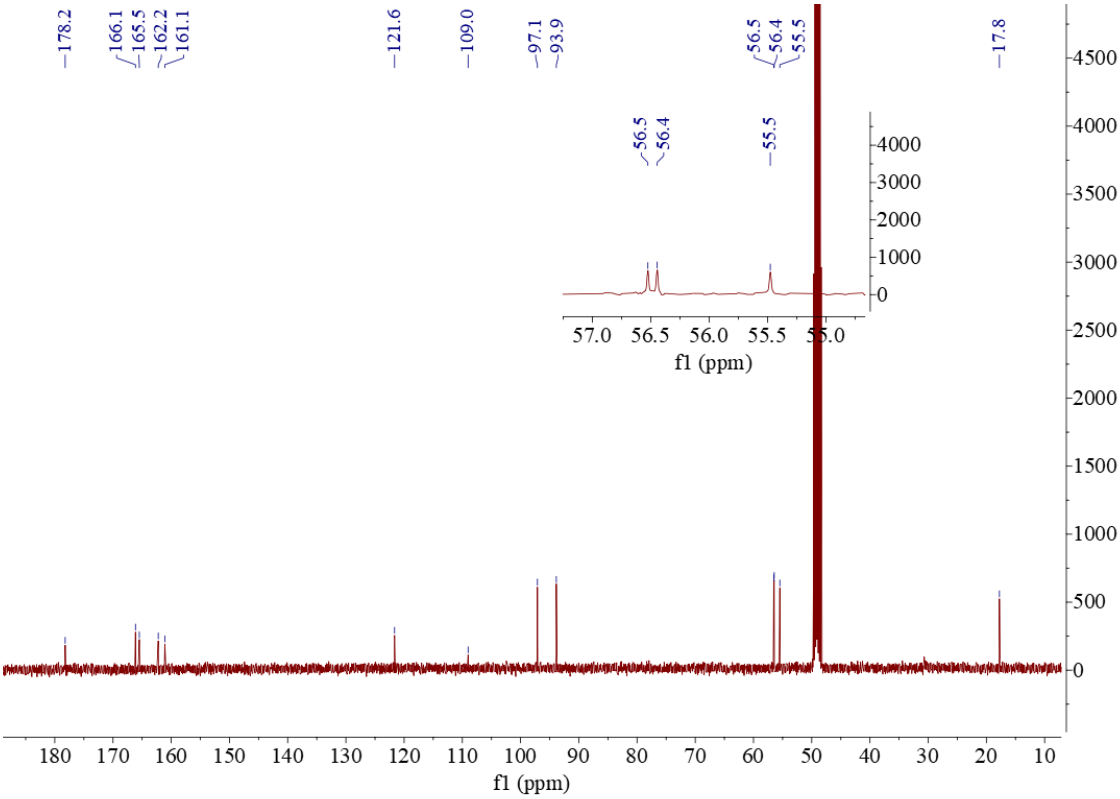

Figure S10. ^{13}C NMR spectrum of **2** in CD_3OD.

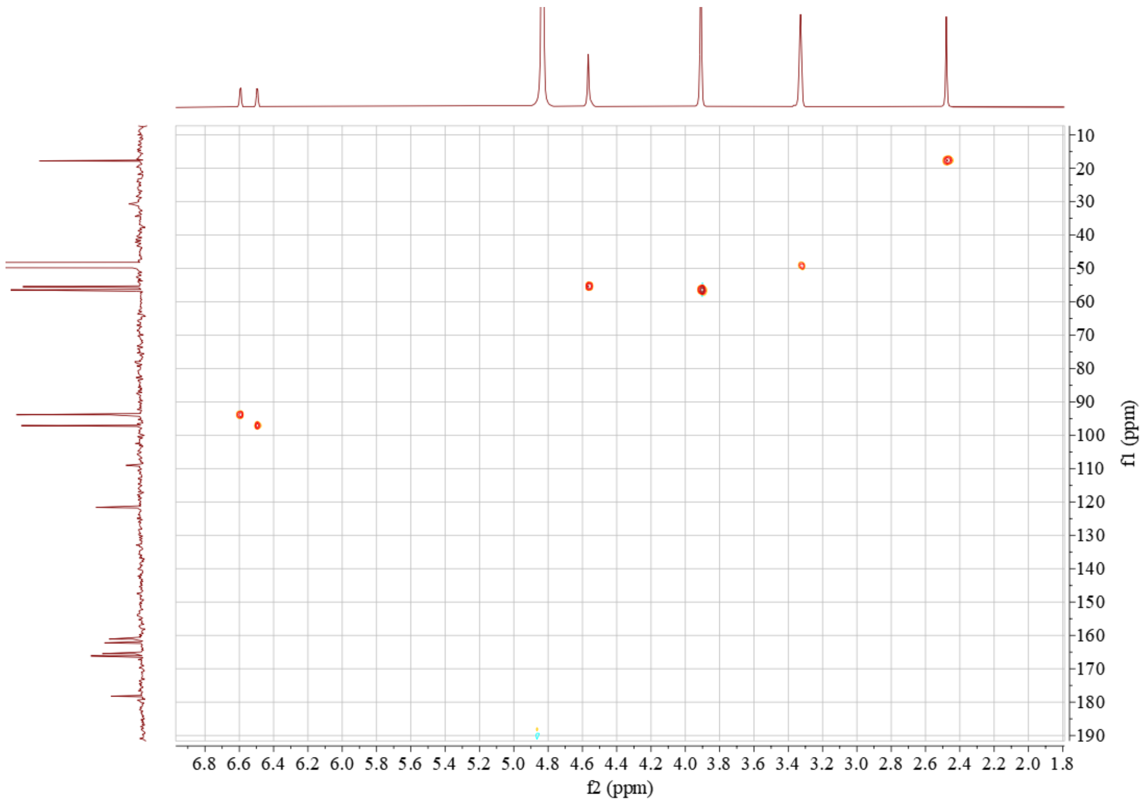

Figure S11. HSQC spectrum of **2**.

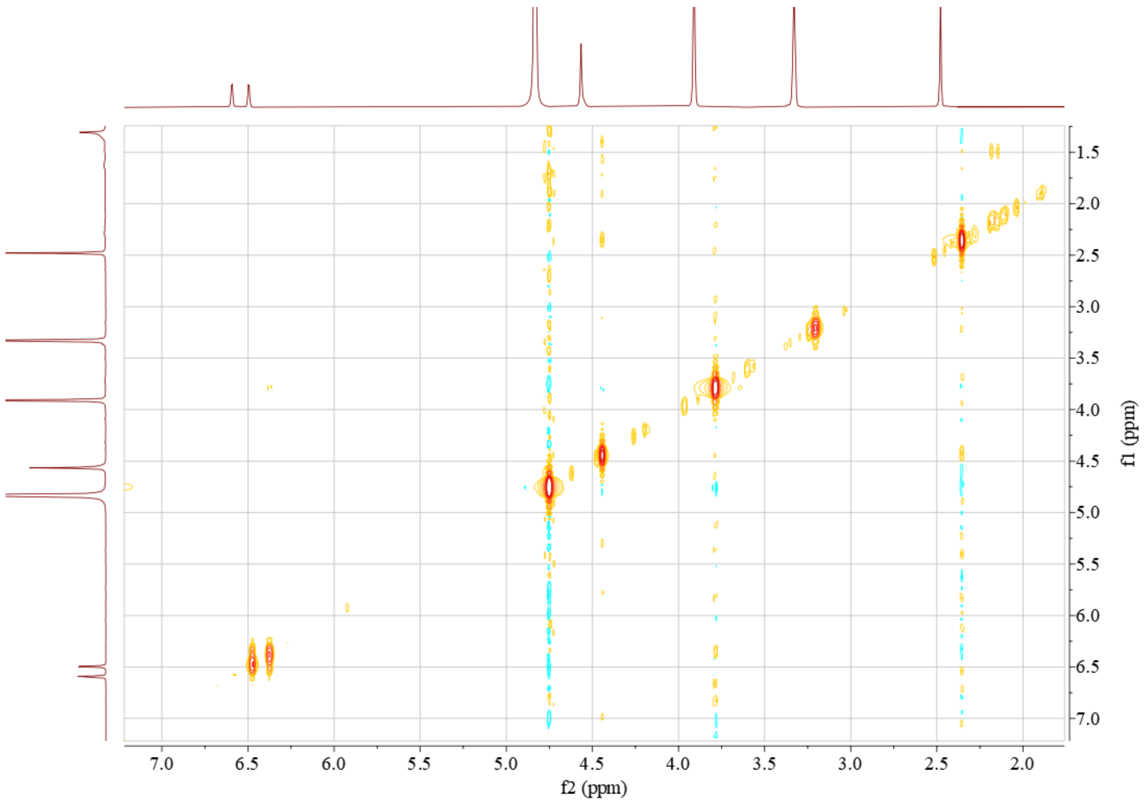

Figure S12. ^1H-^1H COSY spectrum of **2**.

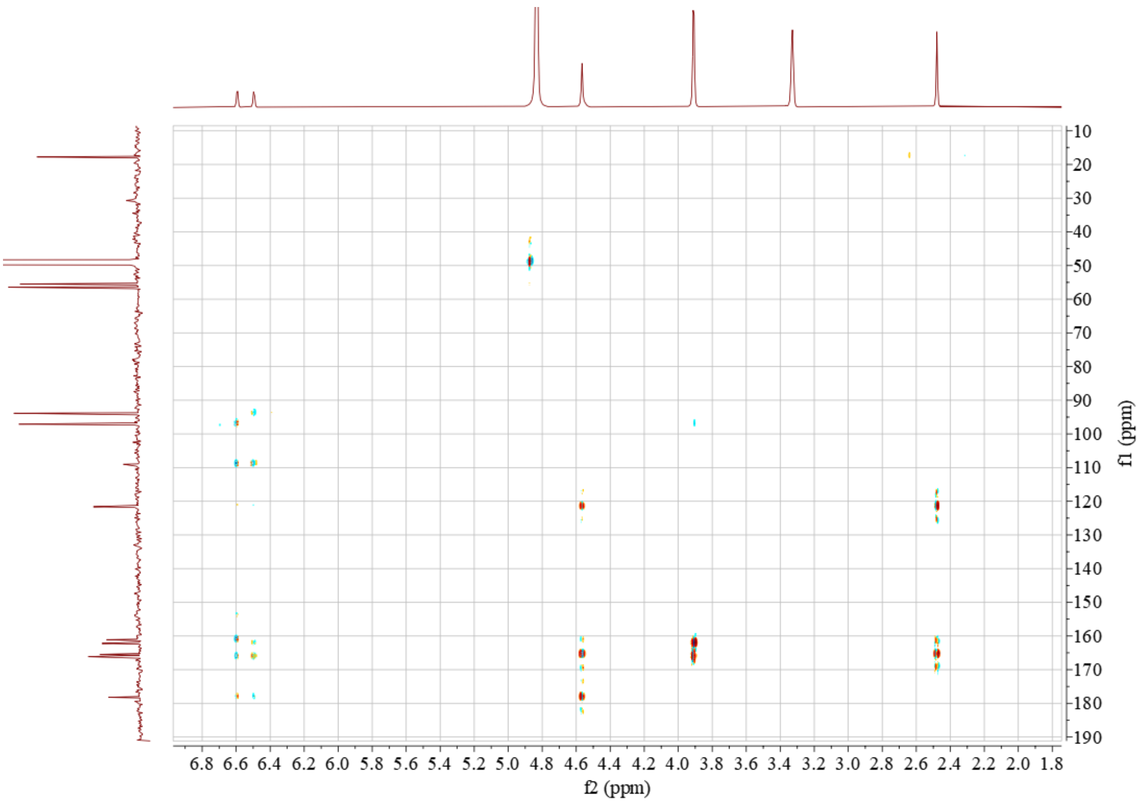

Figure S13. HMBC spectrum of **2**.

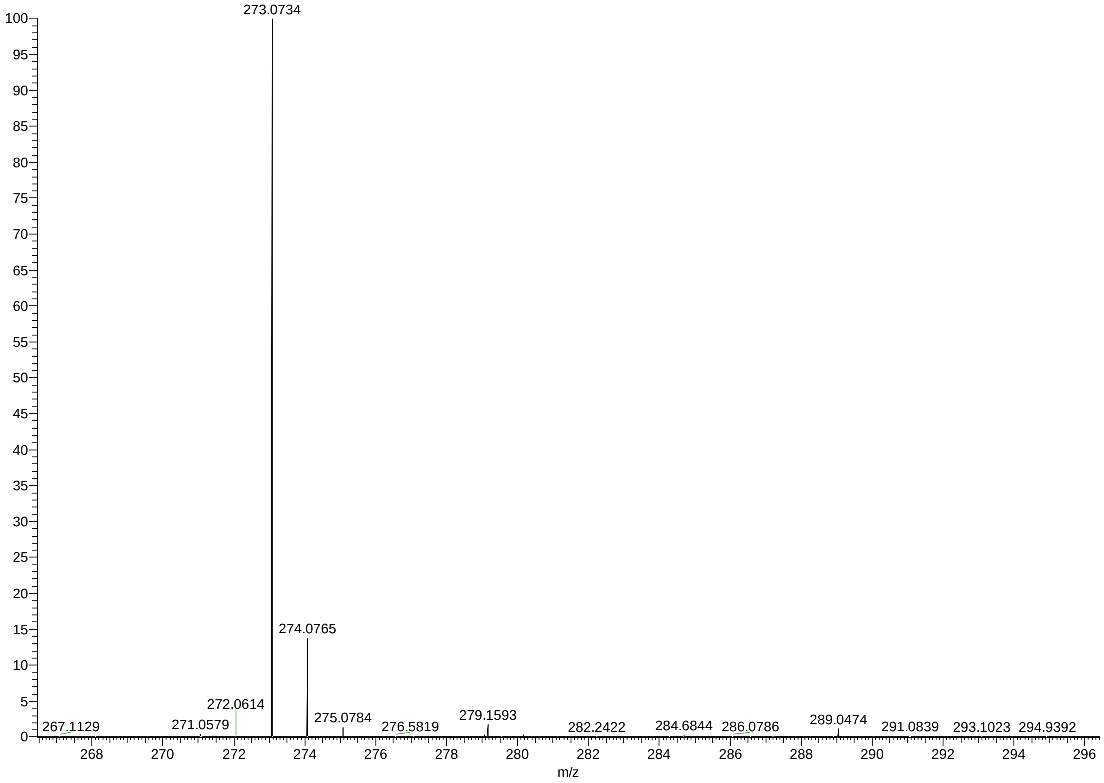

Figure S14. HR-ESI-MS spectrum of **2**.

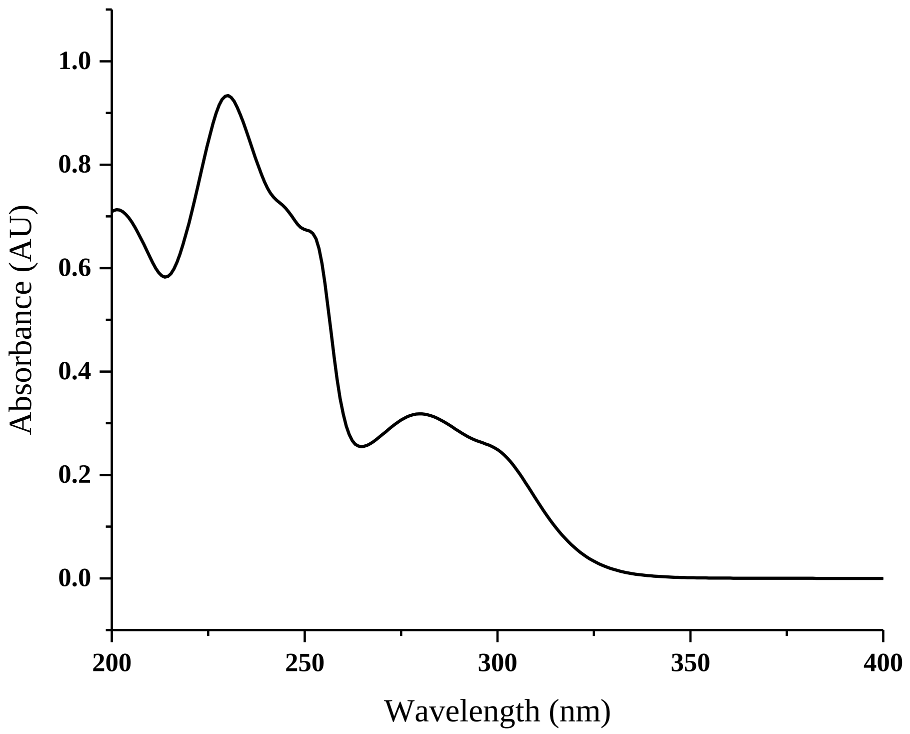

Figure S15. UV-vis spectrum of **2**.

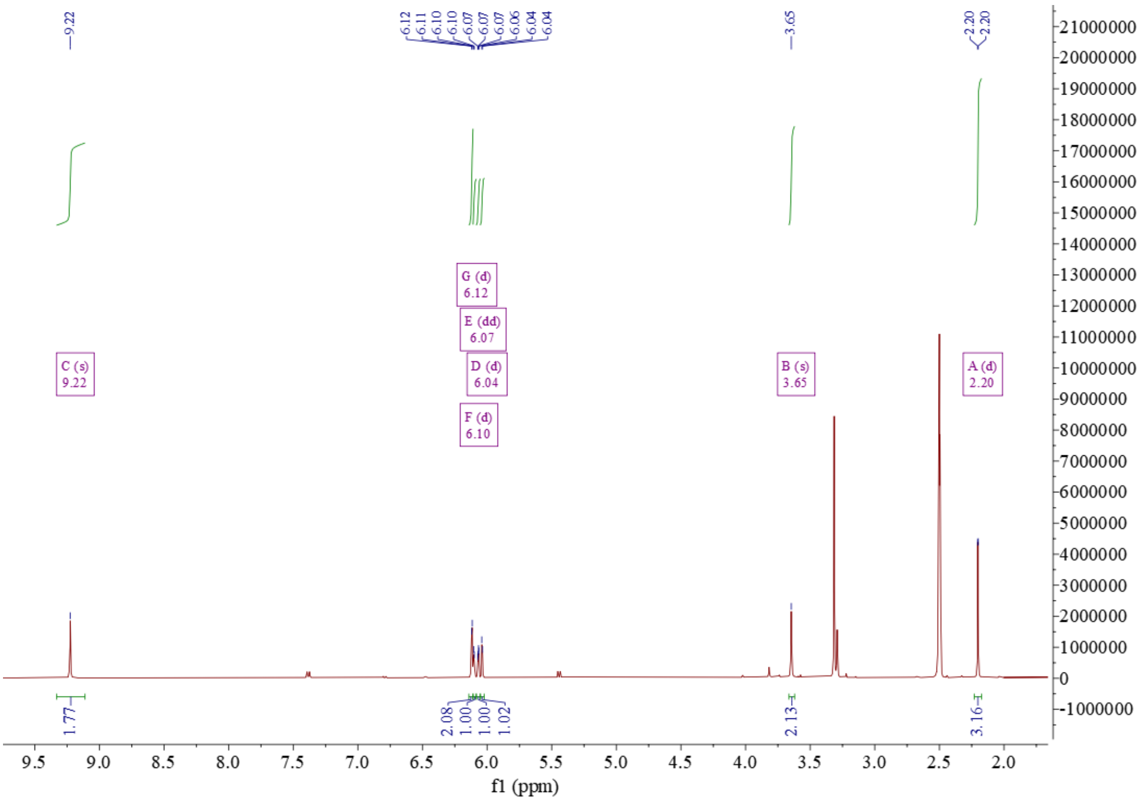

Figure S16. ^{1}H NMR spectrum of **4** in DMSO-d_6.

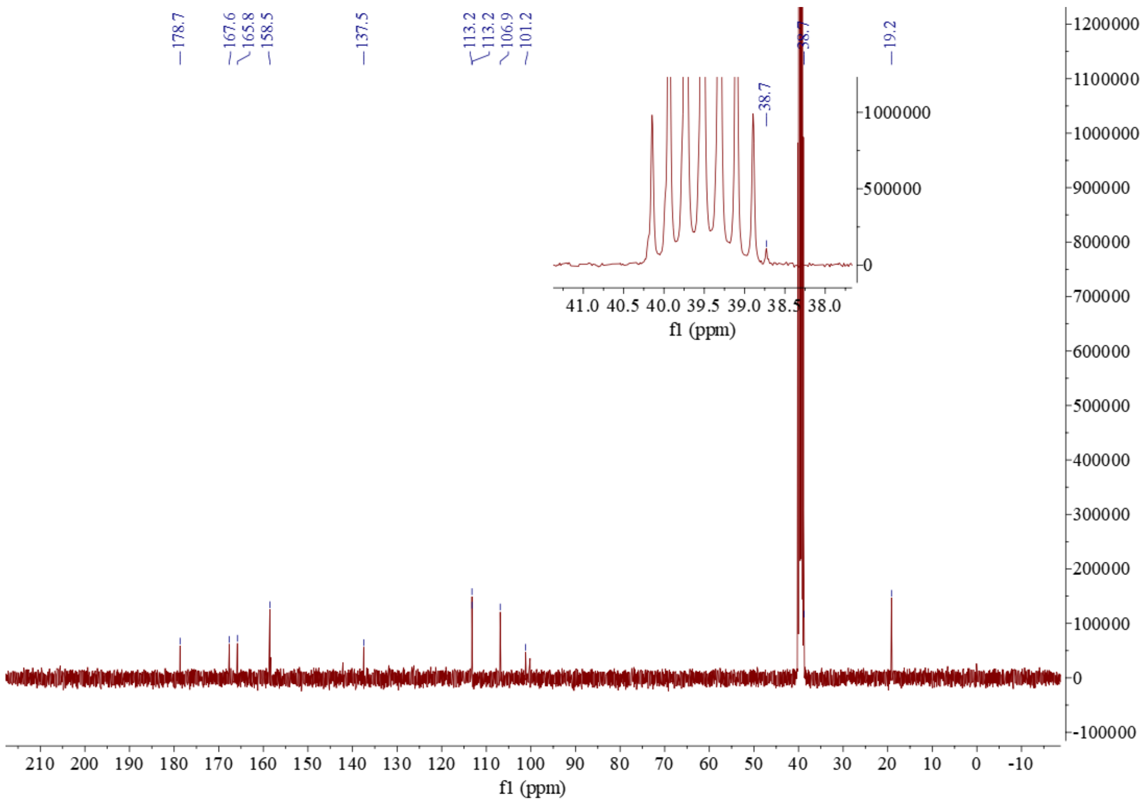

Figure S17. ^{13}C NMR spectrum of **4** in DMSO-*d*6.

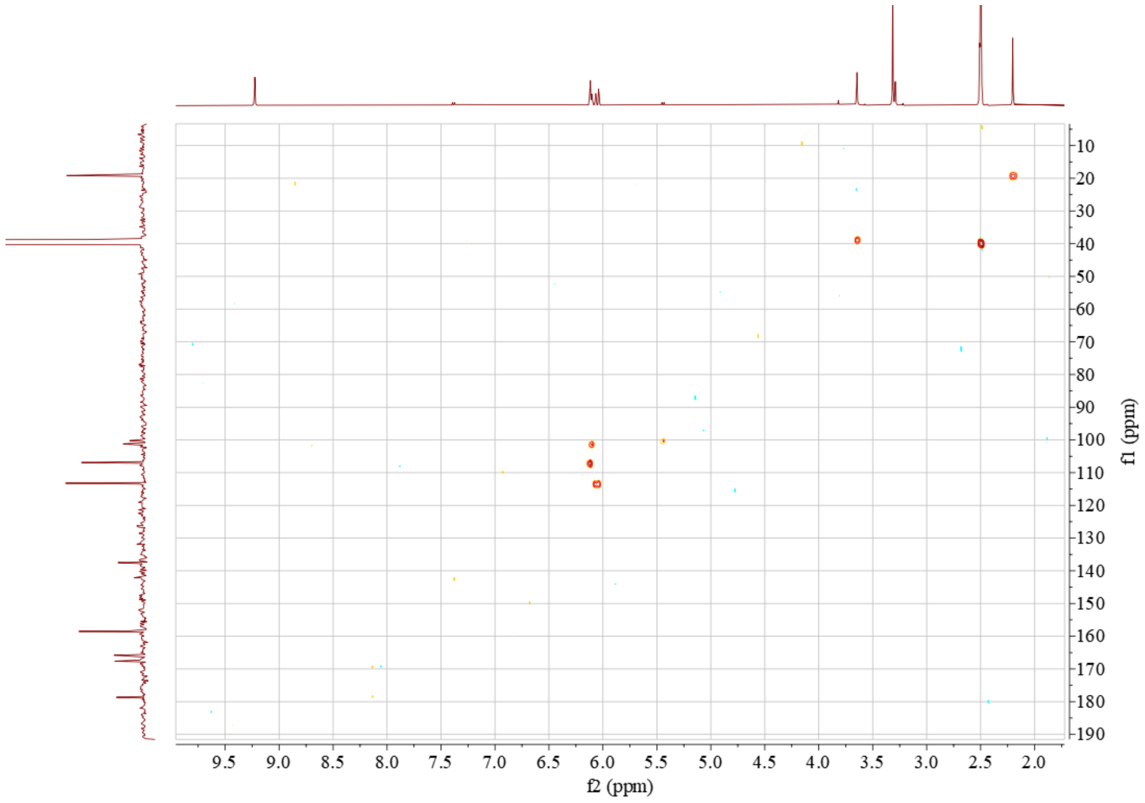

Figure S18. HSQC spectrum of **4**.

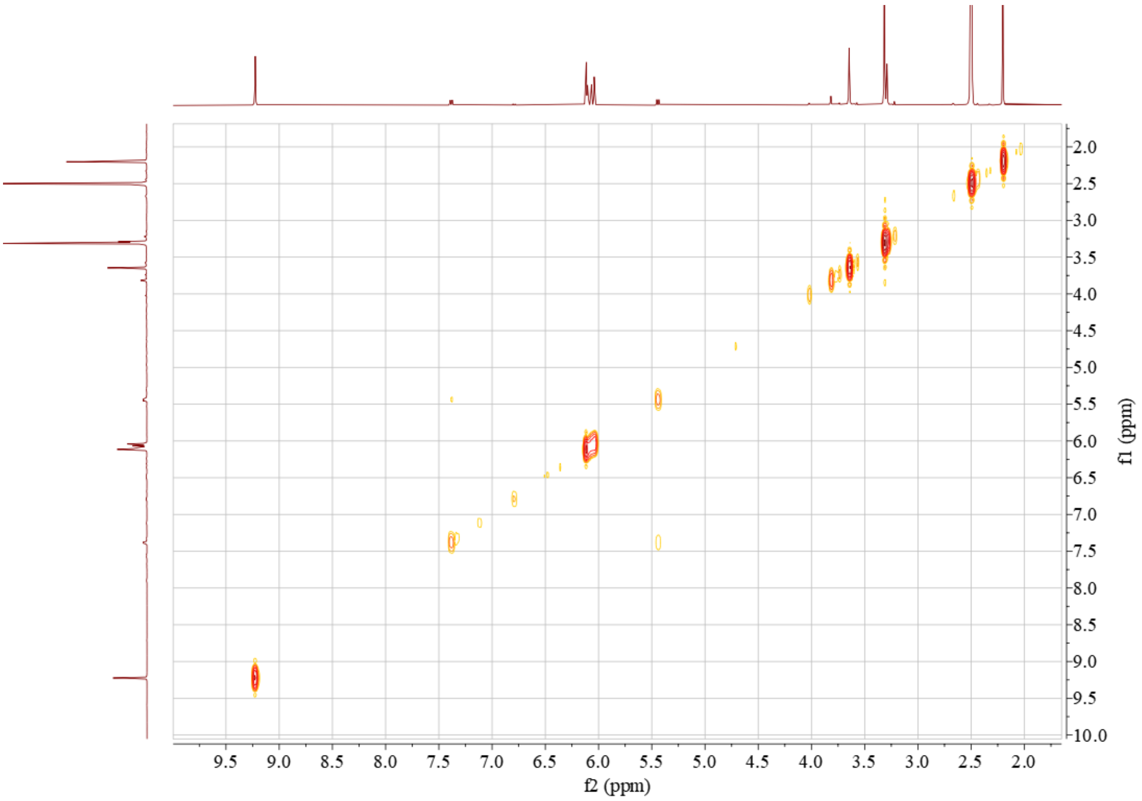

Figure S19. ^1H-^1H COSY spectrum of **4**.

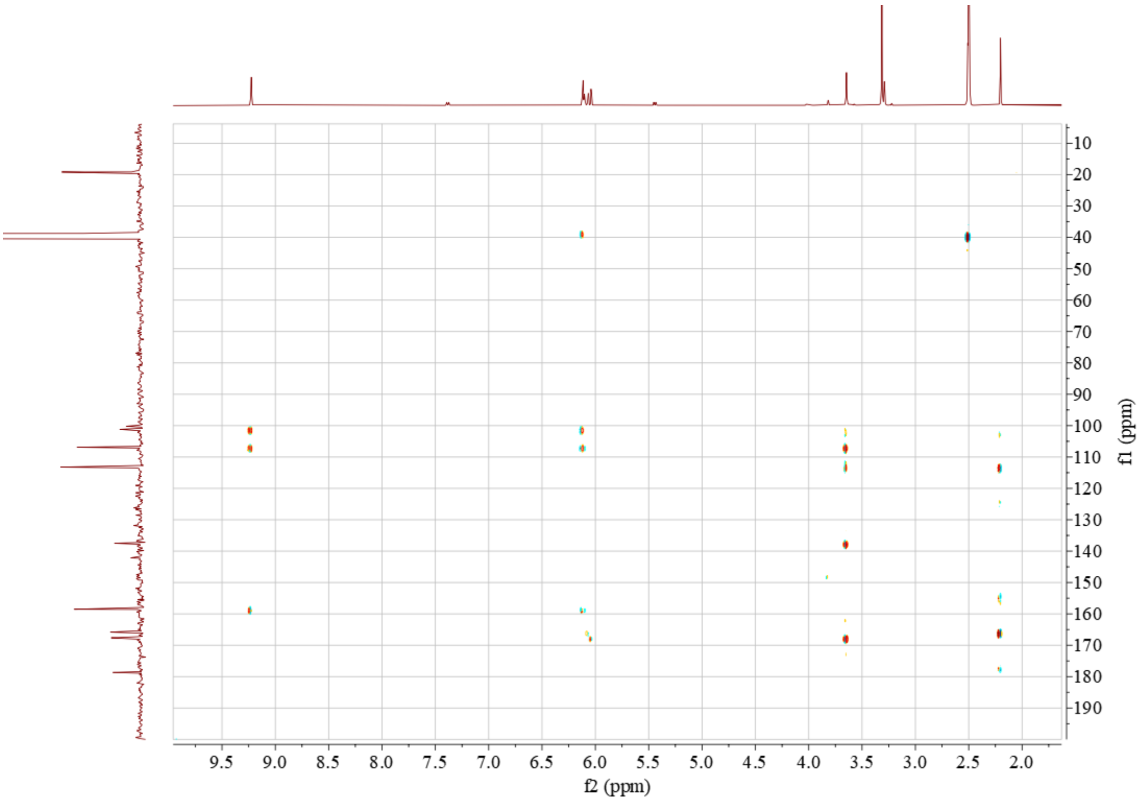

Figure S20. HMBC spectrum of **4**.

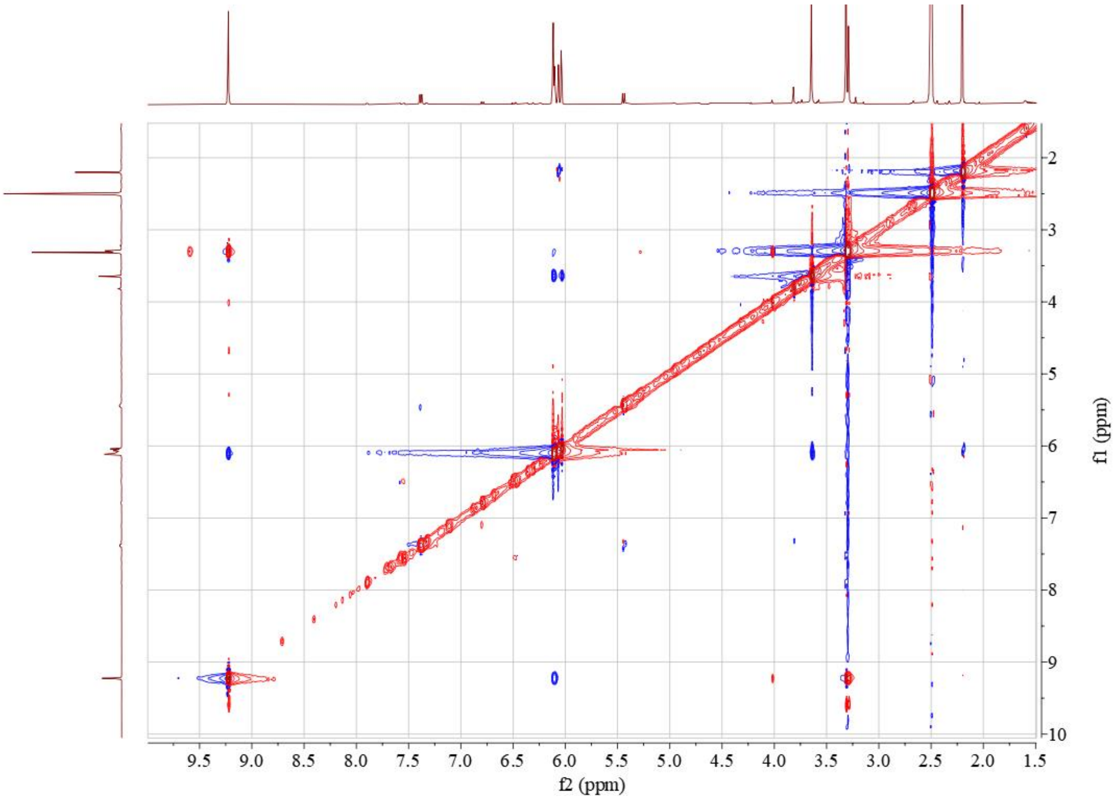

Figure S21. NOESY spectrum of **4**.

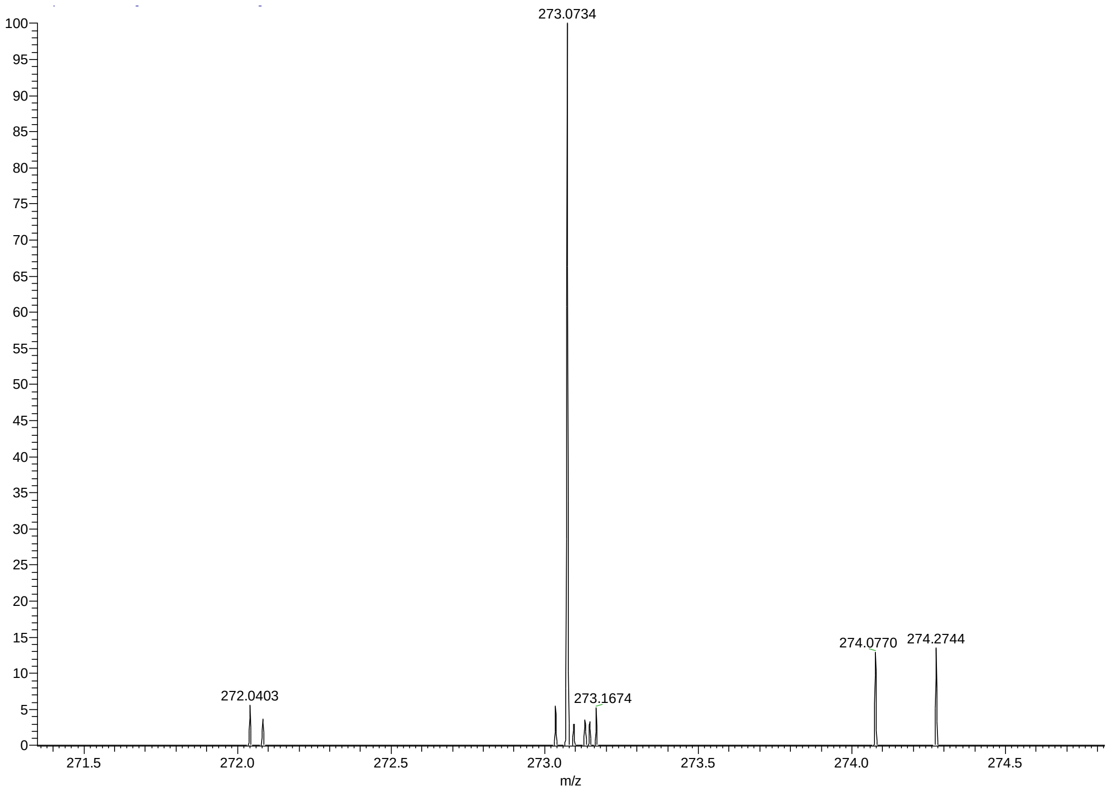

Figure S22. HR-ESI-MS spectrum of **4**.

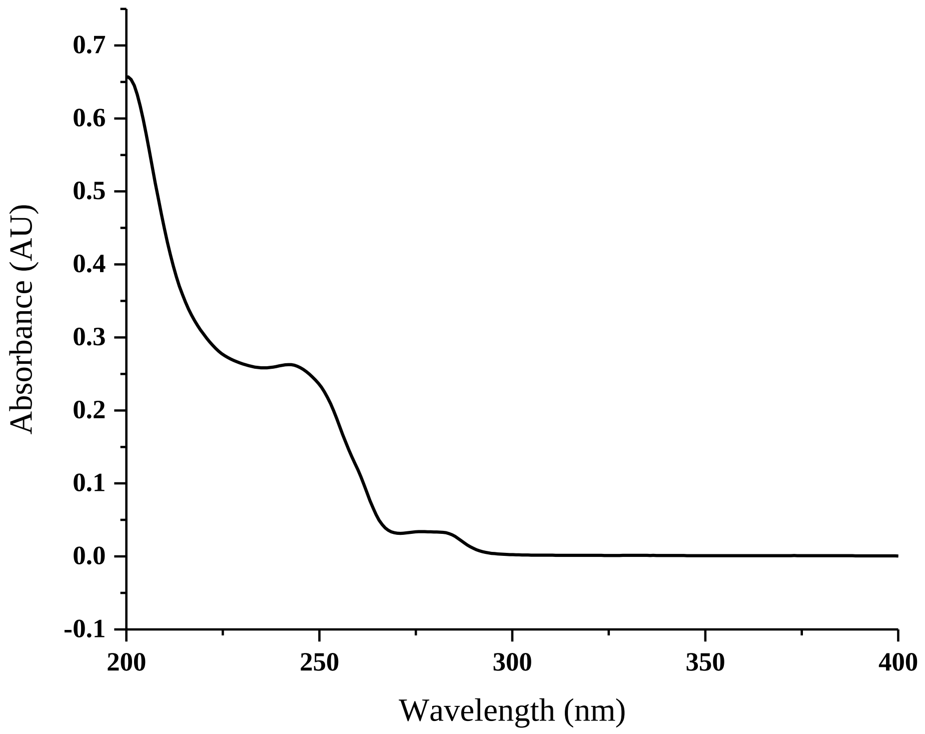

Figure S23. UV-vis spectrum of **4**.

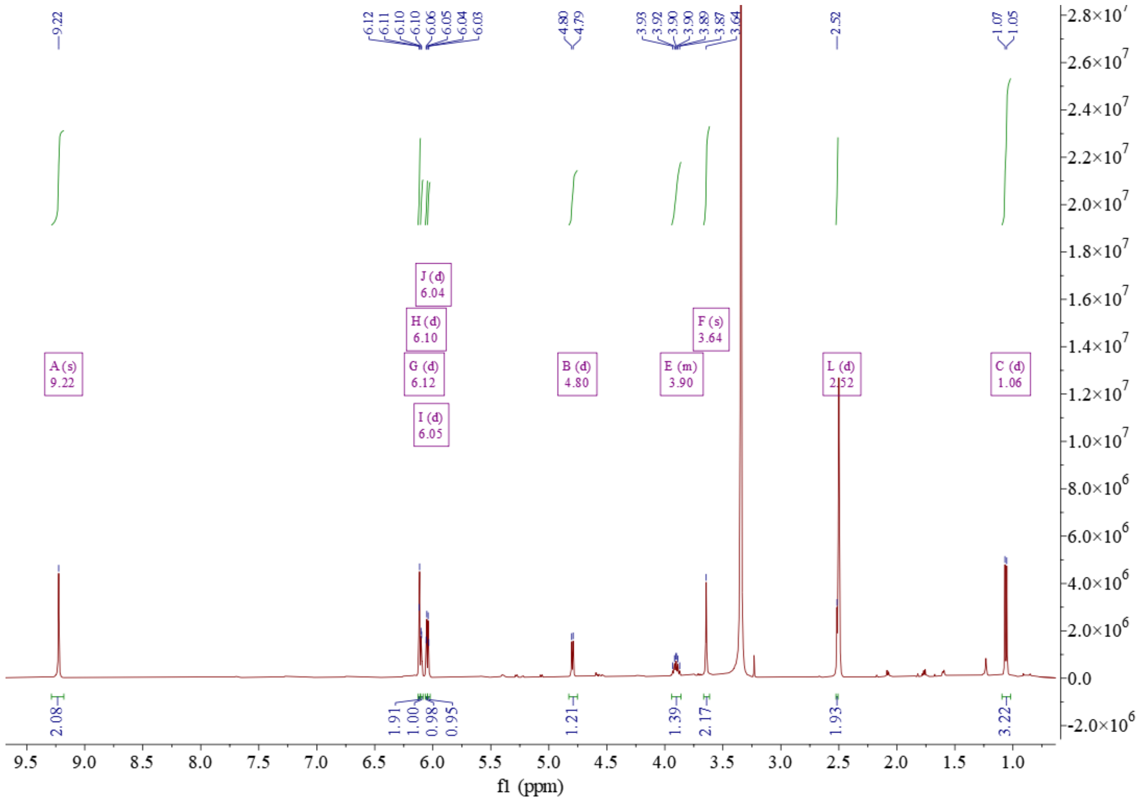

Figure S24. ^{1}H NMR spectrum of **5** in DMSO-d_6.

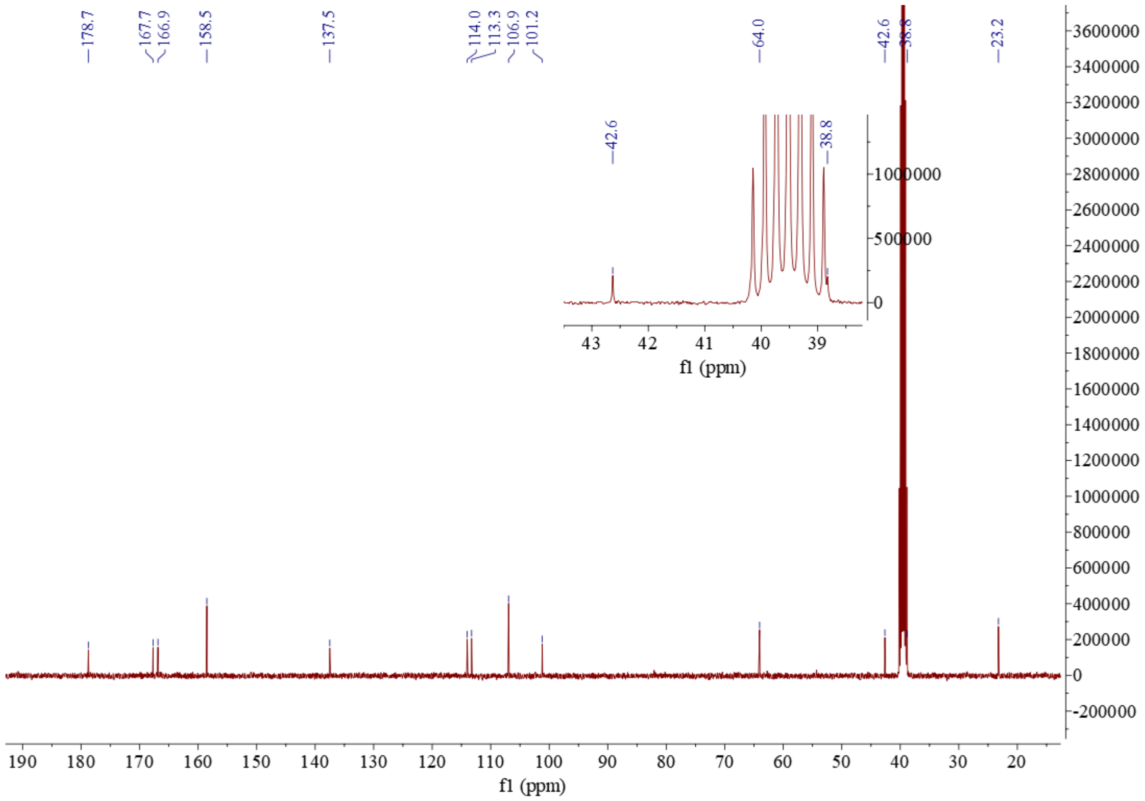

Figure S25. ^{13}C NMR spectrum of **5** in DMSO-d_6.

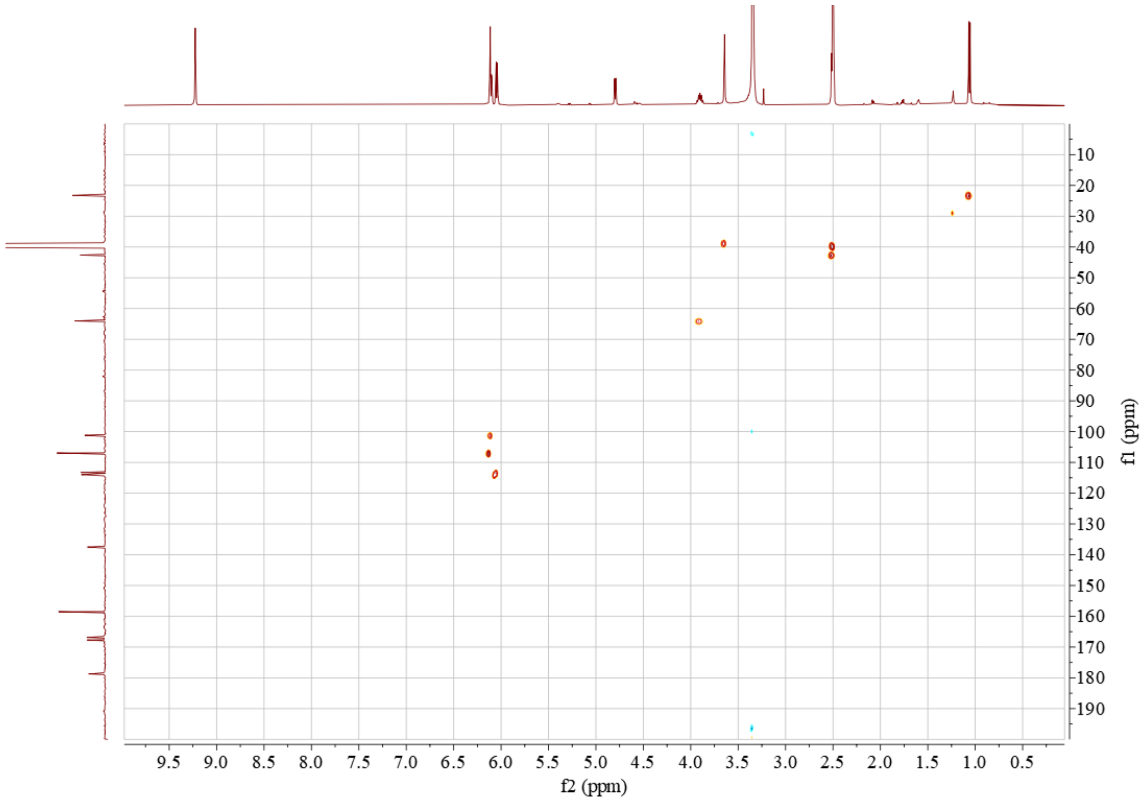

Figure S26. HSQC spectrum of **5**.

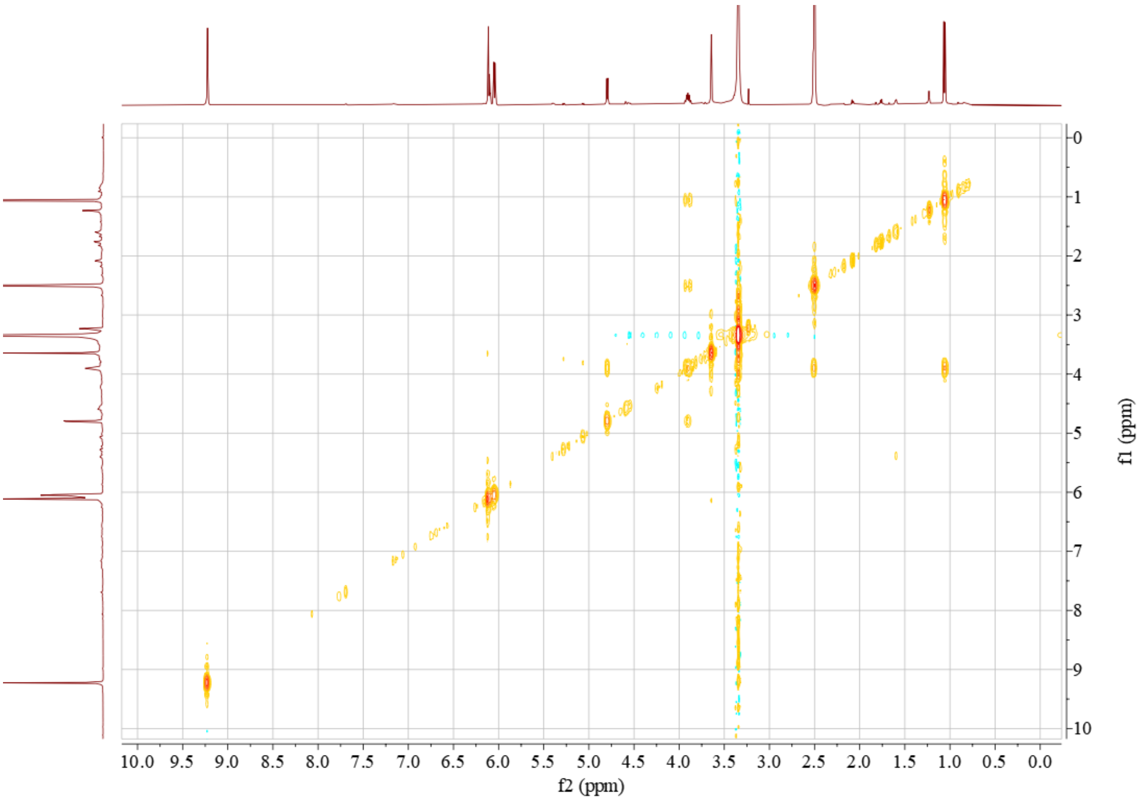

Figure S27. ^1H-^1H COSY spectrum of **5**.

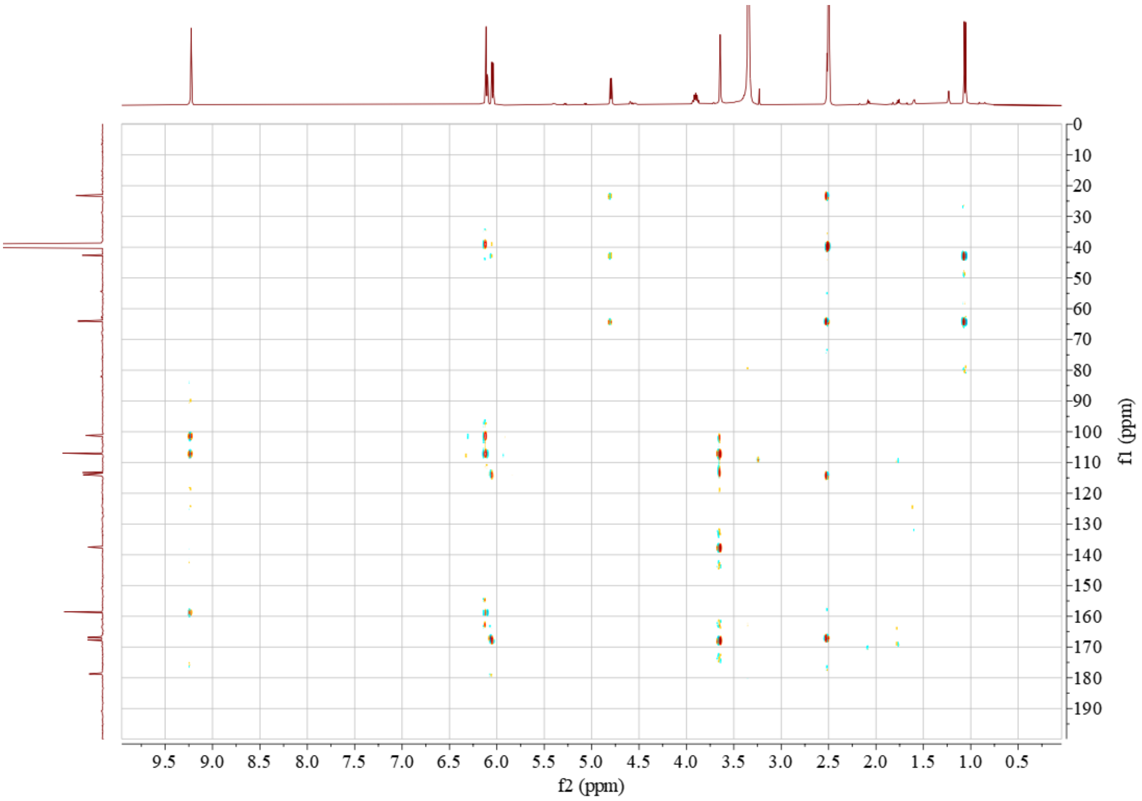

Figure S28. HMBC spectrum of **5**.

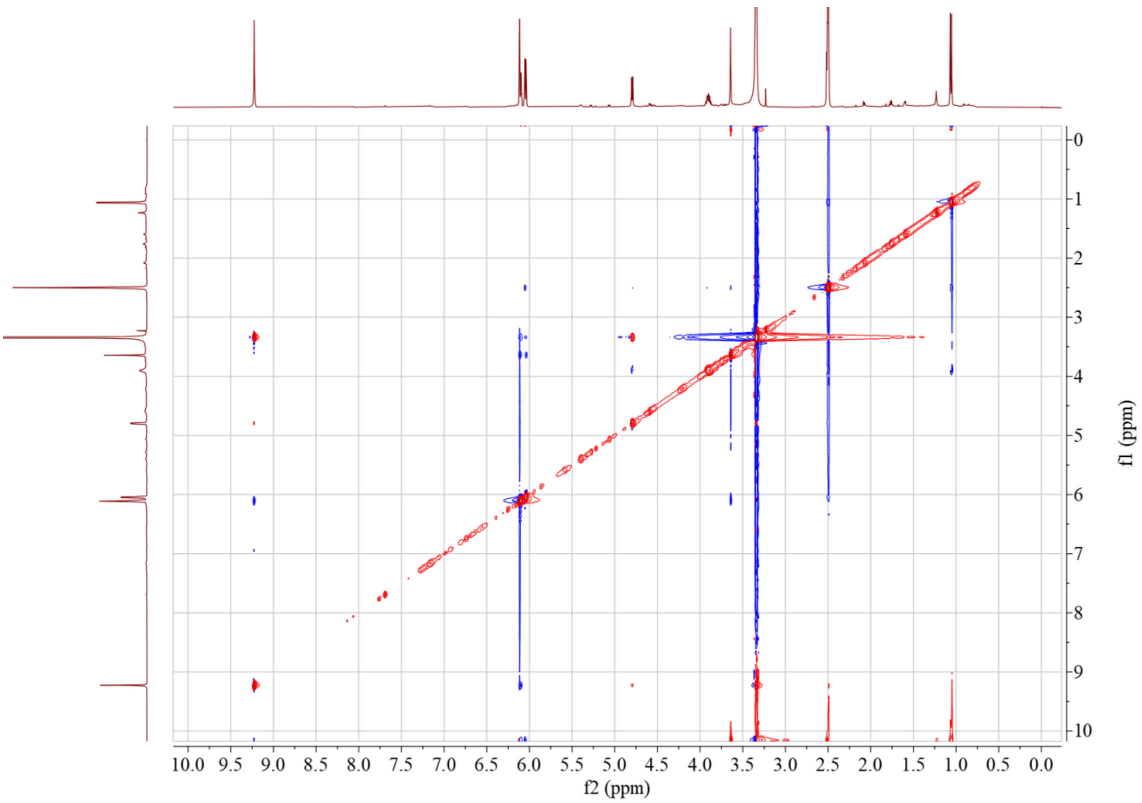

Figure S29. NOESY spectrum of **5**.

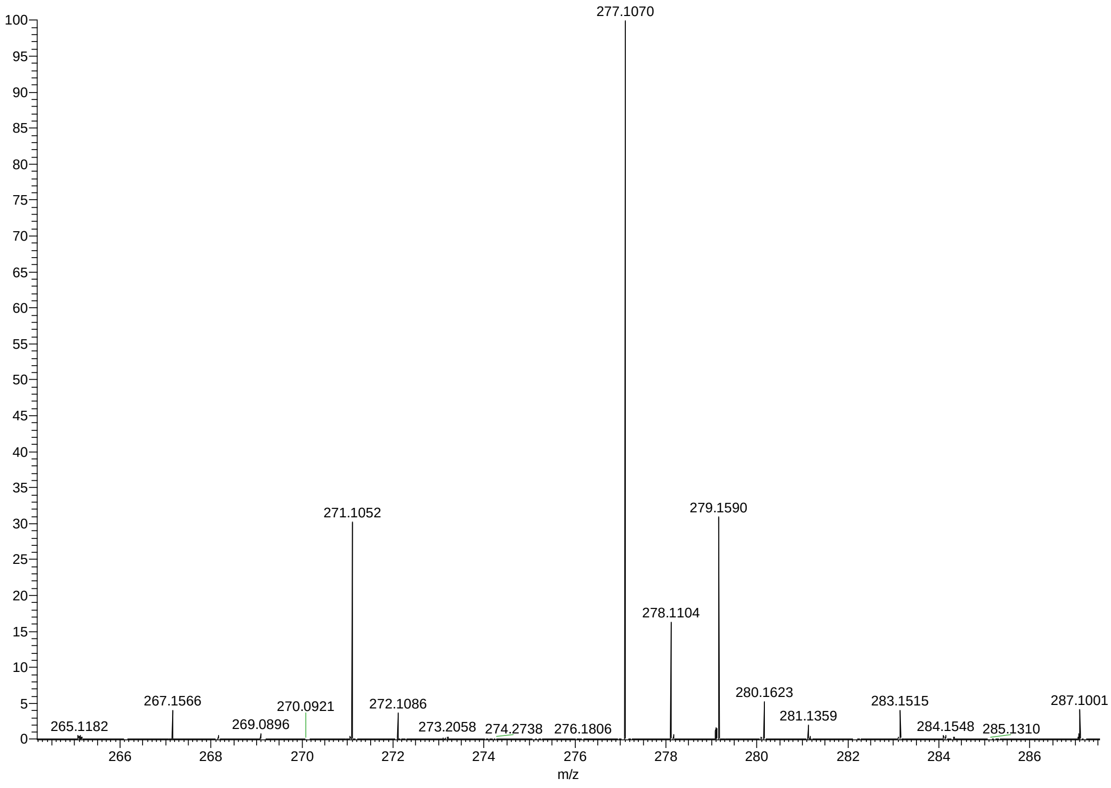

Figure S30. HR-ESI-MS spectrum of **5**.

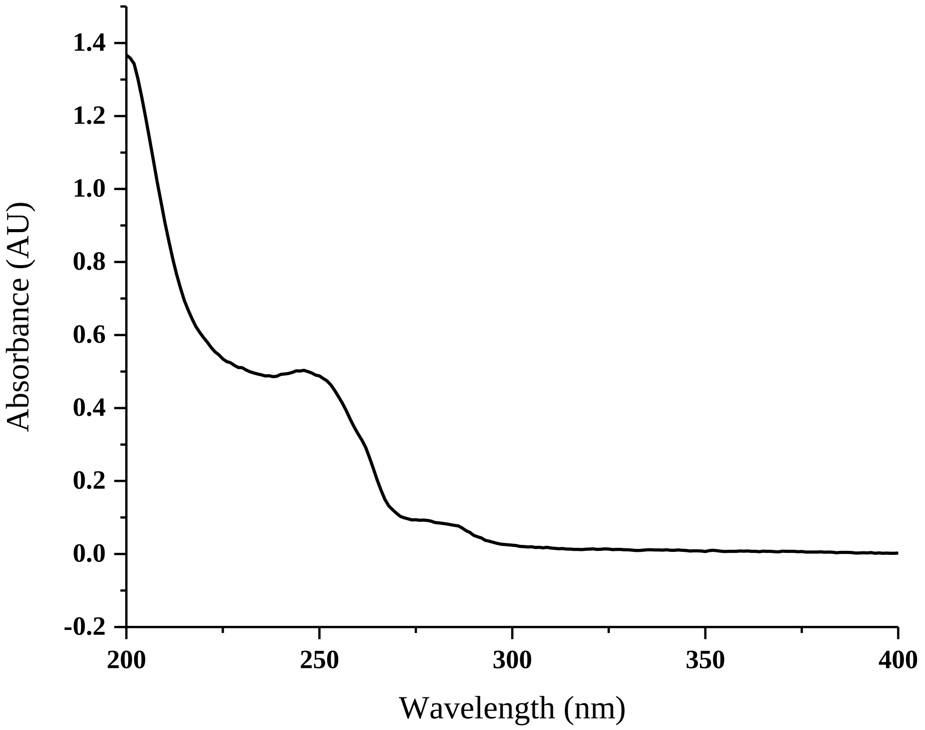

Figure S31. UV-vis spectrum of **5**.

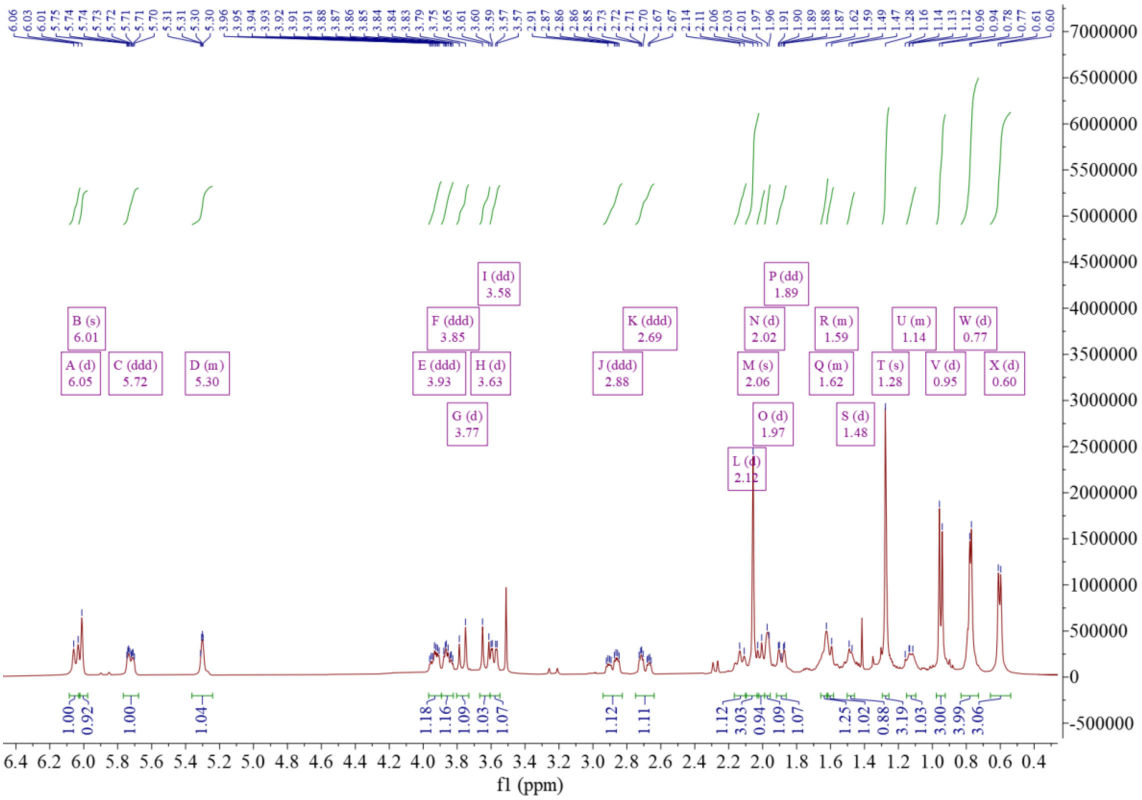

Figure S32. ^{1}H NMR spectrum of **6** in $CDCl_3$.

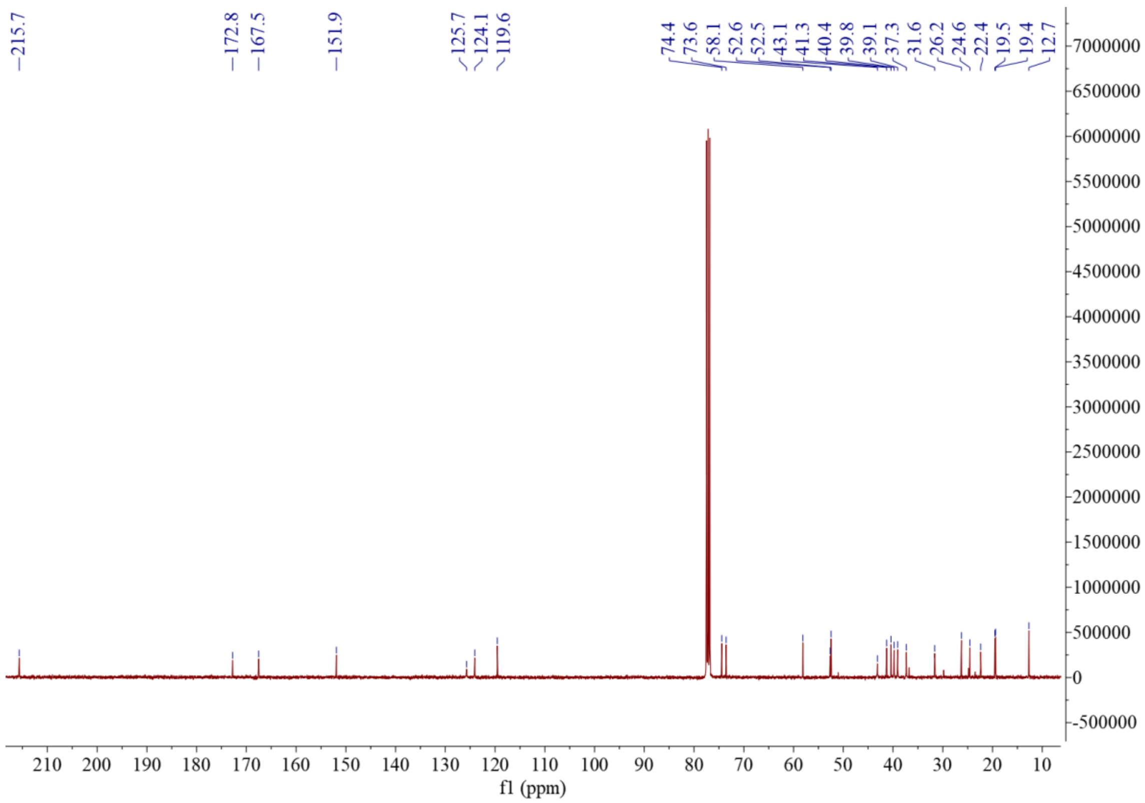

Figure S33. ^{13}C NMR spectrum of **6** in $CDCl_3$.

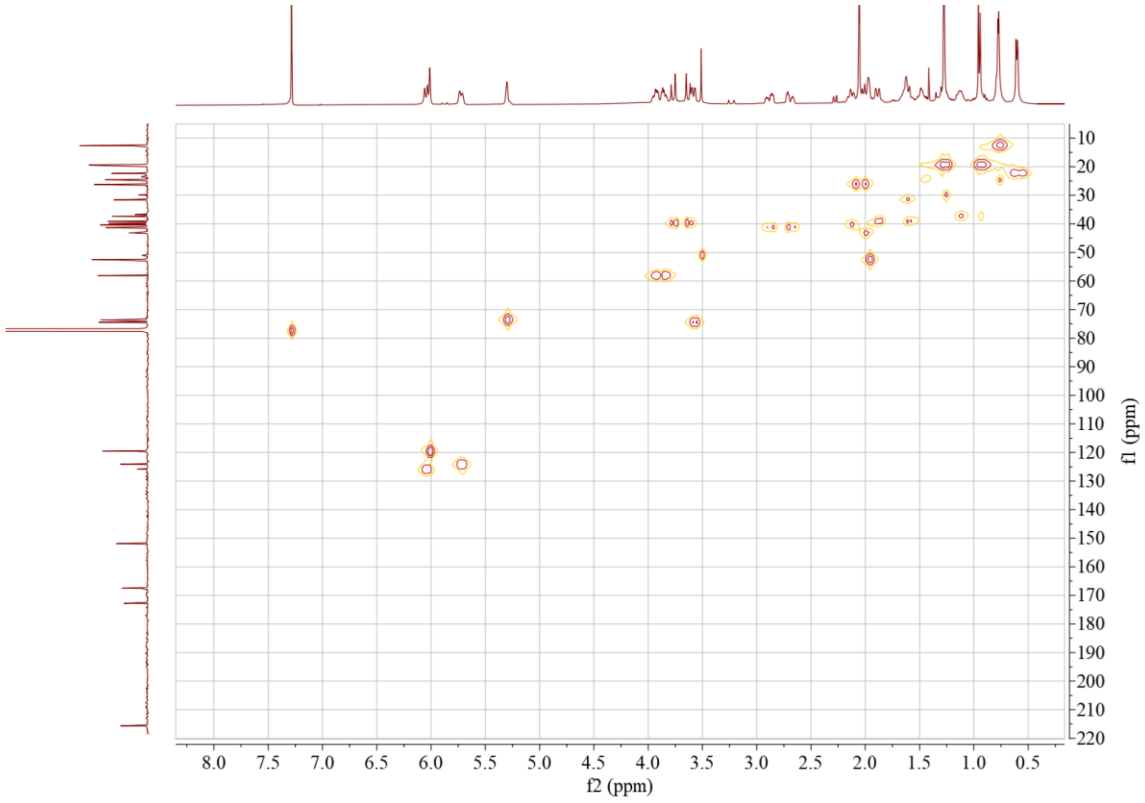

Figure S34. HSQC spectrum of **6**.

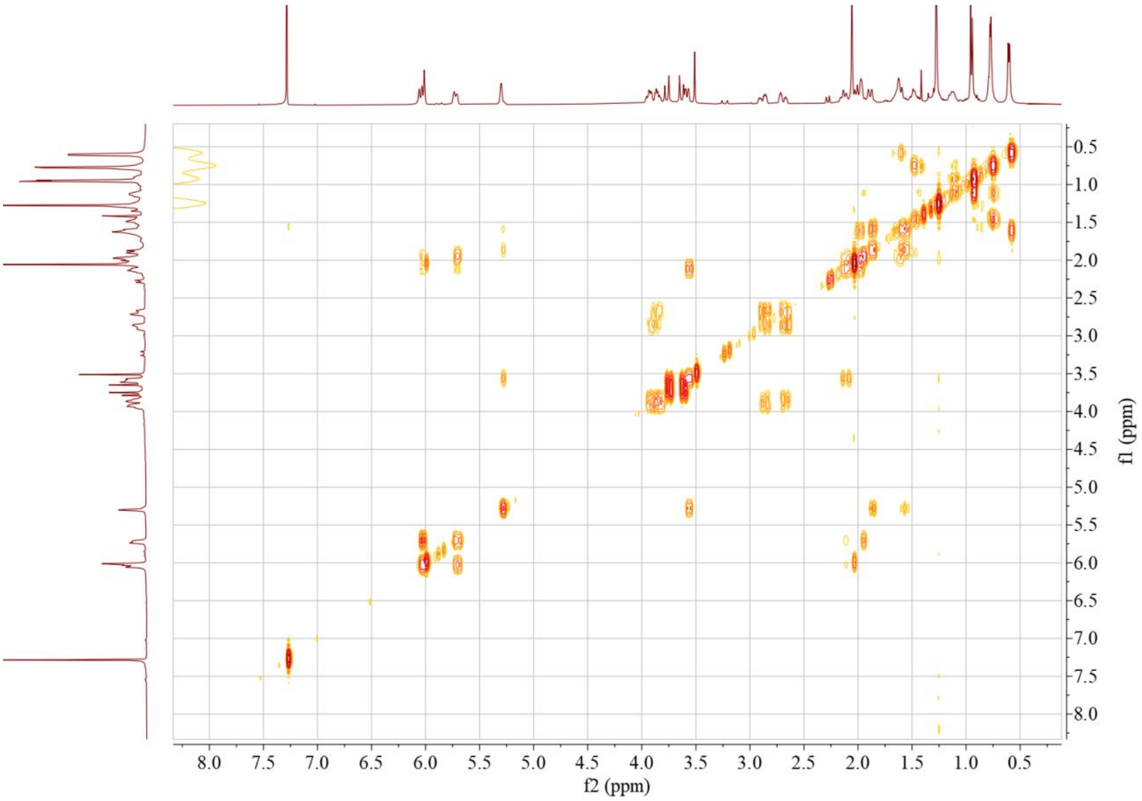

Figure S35. ^{1}H-^{1}H COSY spectrum of **6**.

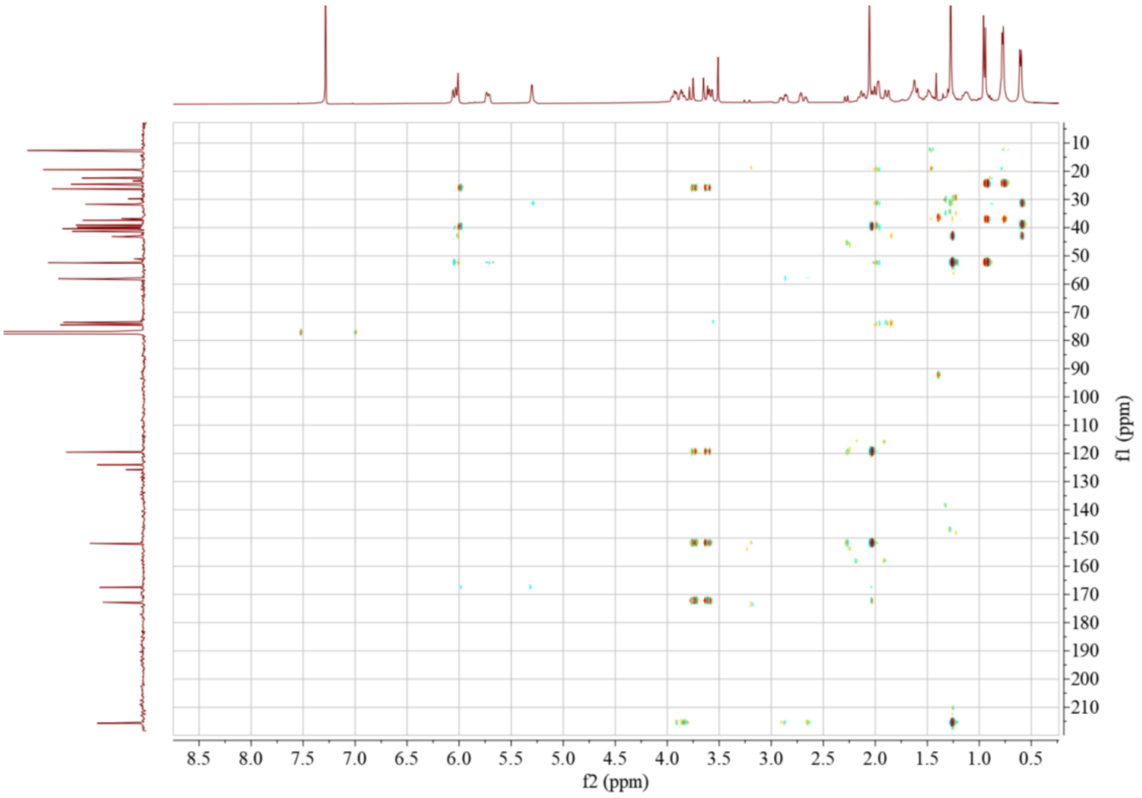

Figure S36. HMBC spectrum of **6**.

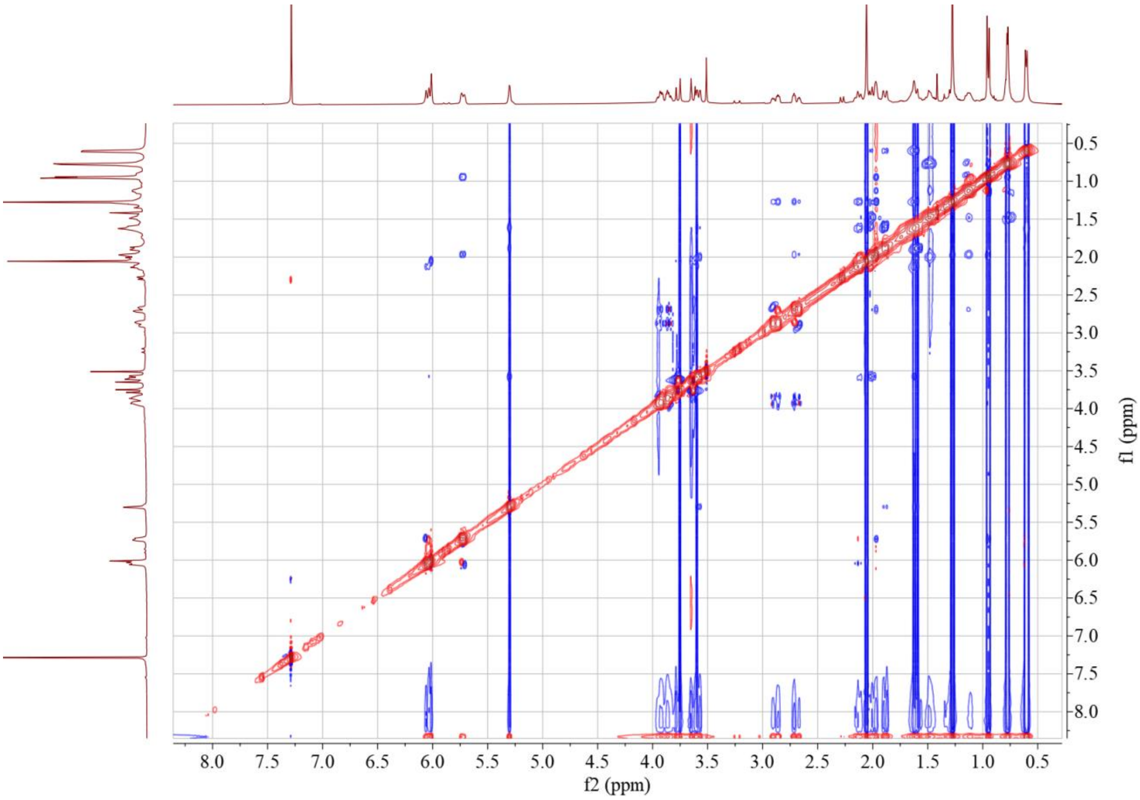

Figure S37. NOESY spectrum of **6**.

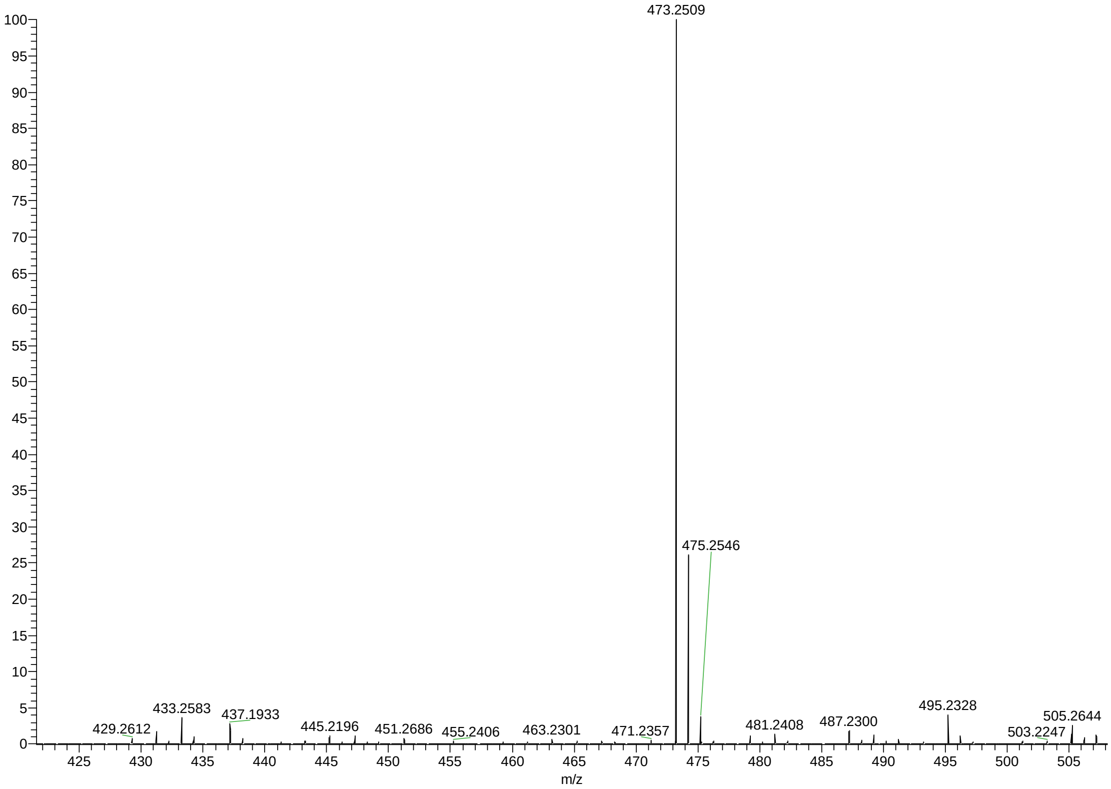

Figure S38. HR-ESI-MS spectrum of **6**.

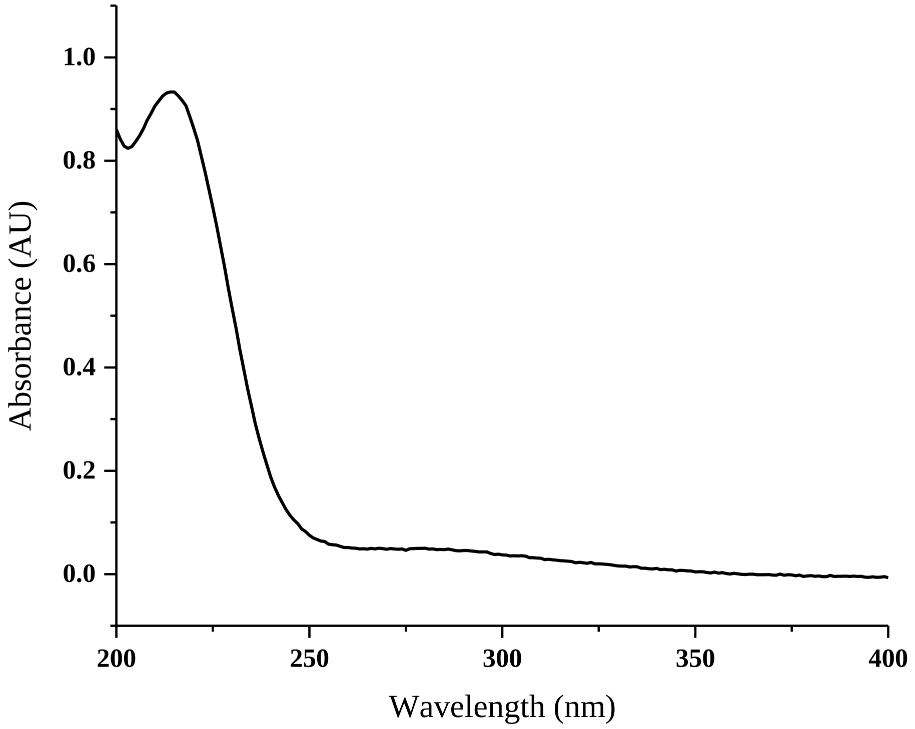

Figure S39. UV-vis spectrum of **6**.

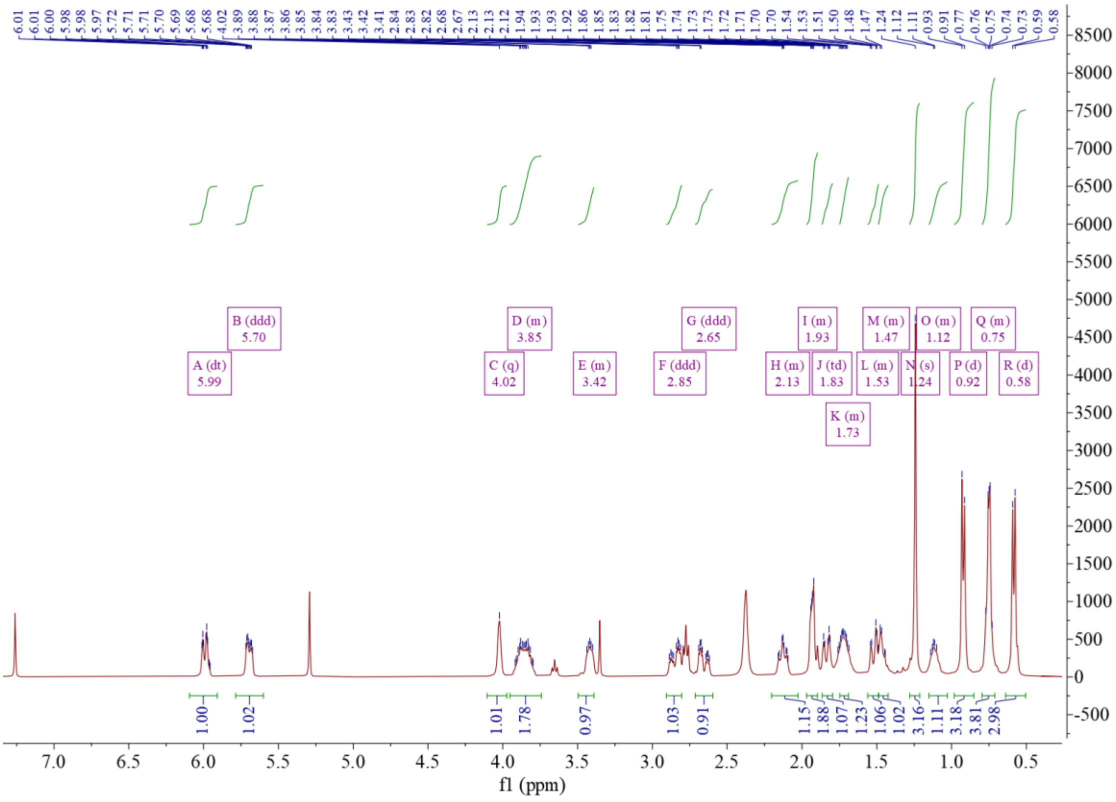

Figure S40. ^{1}H NMR spectrum of eujavanicol A in $CDCl_3$.

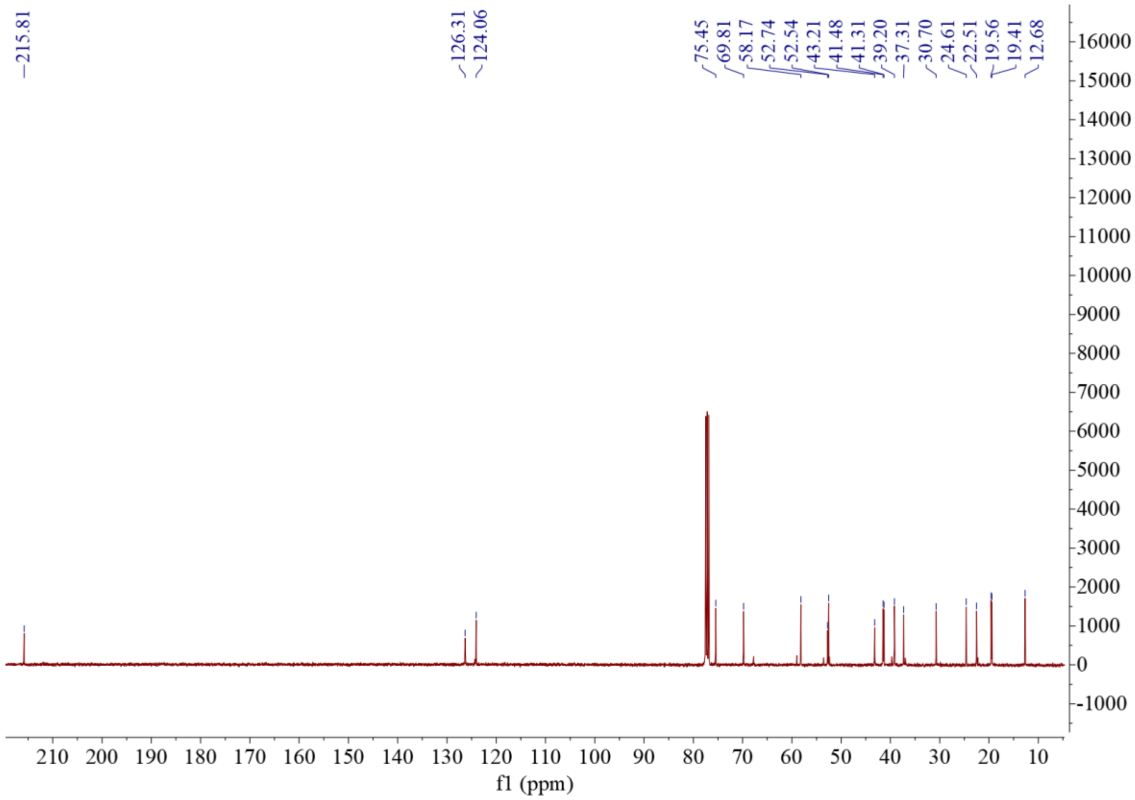

Figure S41. ^{13}C NMR spectrum of eujavanicol A in $CDCl_3$.

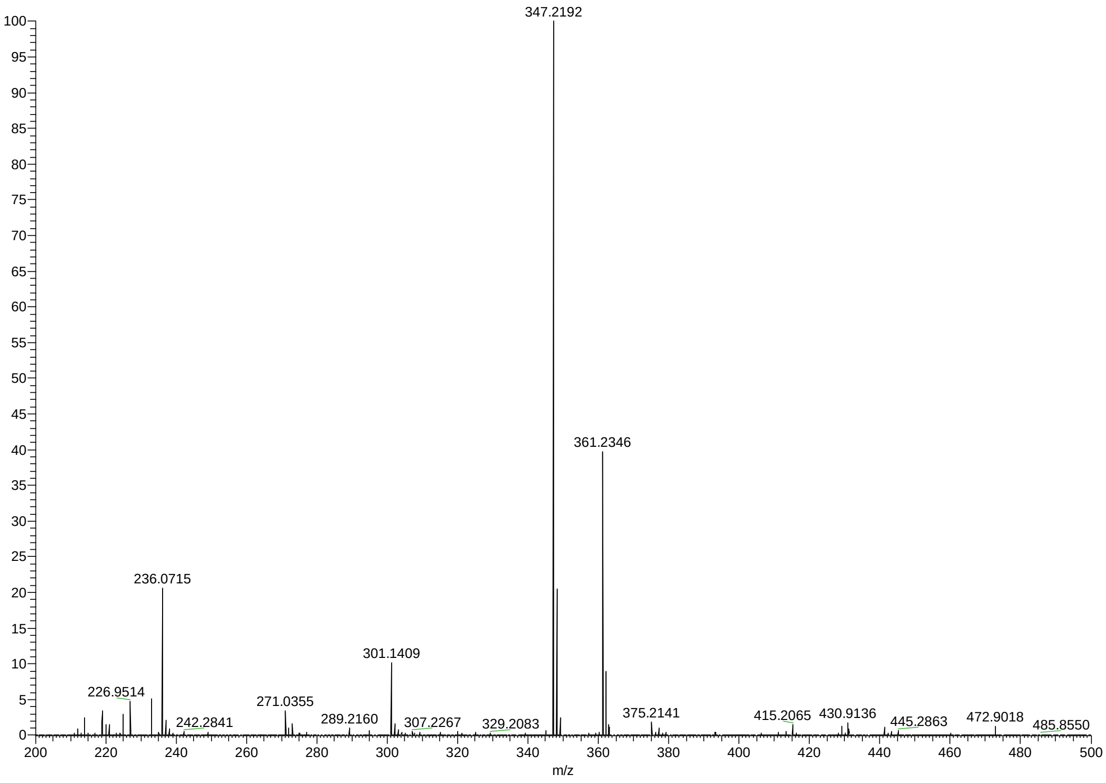

Figure S42. HR-ESI-MS spectrum of eujavanicol A.

Table S1. Gibbs free energy and Boltzmann population of low energy conformers of (3′*R*, 5′*S*)-**1** in MeOH.

Conformers	Δ*G* (kcal/mol)	P
1A	0.00	30.8%
1B	0.05	28.5%
1C	0.32	18.1%
1D	0.88	7.0%
1E	0.90	6.8%
1F	1.17	4.3%
1G	1.26	3.6%

Table S2. Gibbs free energy and Boltzmann population of low energy conformers of (3′*R*, 5′*R*)-**1** in MeOH.

Conformers	Δ*G* (kcal/mol)	P
1AA	0.00	78.4%
1BB	0.77	21.3%

Table S3. Gibbs free energy and Boltzmann population of low energy conformers of 14*S*-**5** in MeOH.

Conformers	Δ*G* (kcal/mol)	P
5A	0.00	16.2%
5B	0.02	15.8%
5C	0.28	10.2%
5D	0.38	8.6%
5E	0.40	8.3%
5F	0.66	5.4%
5G	0.66	5.4%
5H	0.71	4.9%
5I	0.79	4.3%
5J	0.84	3.9%
5K	1.13	2.4%
5L	1.16	2.3%
5M	1.19	2.2%
5N	1.43	1.4%
5O	1.54	1.2%
5P	1.64	1.0%
5Q	1.66	1.0%

Table S4. Cartesian coordinates for the low-energy optimized conformers of (3′*R*, 5′*S*)-**1** at B3LYP/6 – 31+g (d,p) level of theory in MeOH.

1A:

Center Number	Atomic Number	Atomic Type	Coordinates (Angstroms)		
			X	Y	Z
1	6	0	1.678966	-0.886286	-0.013639
2	6	0	1.464588	0.490108	-0.201055
3	6	0	0.127269	0.985305	-0.416828
4	6	0	-0.953858	0.004945	-0.426216
5	6	0	-0.662173	-1.317916	-0.242149
6	8	0	0.615528	-1.747004	-0.042240
7	6	0	-2.318944	0.620143	-0.670225
8	6	0	-1.594212	-2.493898	-0.210231
9	6	0	2.935135	-1.430110	0.200102
10	6	0	4.033969	-0.555005	0.227393
11	6	0	3.873630	0.830450	0.044582
12	6	0	2.597724	1.348094	-0.168040
13	8	0	5.234983	-1.144992	0.439440
14	6	0	6.418847	-0.334998	0.480167
15	8	0	2.440659	2.675523	-0.342956
16	8	0	-0.100606	2.217016	-0.587396
17	8	0	-3.311702	-0.352444	-1.119149
18	6	0	-4.316757	-0.487893	-0.228867
19	6	0	-4.008286	0.316694	1.036470
20	6	0	-2.964019	1.318714	0.557056
21	8	0	-5.293059	-1.171788	-0.447297
22	8	0	-5.151660	0.962777	1.561540
23	1	0	-2.211037	1.327493	-1.493297
24	1	0	-1.006312	-3.411818	-0.172356
25	1	0	-2.232566	-2.465214	0.678872
26	1	0	-2.238971	-2.514601	-1.089019
27	1	0	3.071145	-2.495243	0.341028
28	1	0	4.712697	1.512999	0.063729
29	1	0	7.239069	-1.029494	0.657733
30	1	0	6.572455	0.180876	-0.472787
31	1	0	6.367981	0.391544	1.297119
32	1	0	1.462890	2.826182	-0.475695
33	1	0	-3.585210	-0.383007	1.772229
34	1	0	-3.473140	2.232860	0.237913
35	1	0	-2.229082	1.576071	1.319048
36	1	0	-5.794354	0.288576	1.828824

1B:

Center Number	Atomic Number	Atomic Type	Coordinates (Angstroms) X	Y	Z
1	6	0	1.779026	-0.878086	0.071474
2	6	0	1.486021	0.491817	-0.052610
3	6	0	0.112962	0.939479	-0.088453
4	6	0	-0.919982	-0.091170	0.015700
5	6	0	-0.546993	-1.400566	0.142290
6	8	0	0.755741	-1.785094	0.159983
7	6	0	-2.344767	0.397909	-0.014029
8	6	0	-1.419267	-2.607152	0.273023
9	6	0	3.071459	-1.372582	0.110585
10	6	0	4.130535	-0.452732	0.022378
11	6	0	3.893594	0.927334	-0.101845
12	6	0	2.581048	1.395025	-0.139325
13	8	0	5.370139	-0.996727	0.066830
14	6	0	6.517630	-0.138414	-0.011637
15	8	0	2.354591	2.718009	-0.258737
16	8	0	-0.170793	2.165011	-0.197247
17	8	0	-3.147331	-0.335931	-1.010954
18	6	0	-4.437716	-0.408470	-0.621336
19	6	0	-4.607665	0.232516	0.768684
20	6	0	-3.174491	0.299550	1.280448
21	8	0	-5.301856	-0.890393	-1.323862
22	8	0	-5.119253	1.557343	0.607944
23	1	0	-2.315528	1.433139	-0.356902
24	1	0	-1.244835	-3.080874	1.245254
25	1	0	-2.474328	-2.371343	0.173788
26	1	0	-1.150843	-3.334290	-0.500129
27	1	0	3.265357	-2.433681	0.207056
28	1	0	4.700517	1.644805	-0.169291
29	1	0	7.379925	-0.801412	0.046979
30	1	0	6.534839	0.405627	-0.961155
31	1	0	6.536387	0.566068	0.825762
32	1	0	1.362482	2.831140	-0.265508
33	1	0	-5.272070	-0.373838	1.391064
34	1	0	-3.008889	1.156357	1.936497
35	1	0	-2.932193	-0.611502	1.834028
36	1	0	-6.039087	1.505326	0.308834

1C:

Center Number	Atomic Number	Atomic Type	Coordinates (Angstroms) X	Y	Z
1	6	0	-1.835070	1.015213	0.005598
2	6	0	-1.172253	-0.225047	-0.031954
3	6	0	0.271680	-0.274717	-0.051064
4	6	0	0.970234	1.002115	-0.030979
5	6	0	0.250589	2.164434	0.022684
6	8	0	-1.105467	2.176548	0.032351
7	6	0	2.476406	1.025807	-0.071310
8	6	0	0.789067	3.564043	0.054059
9	6	0	-3.214317	1.132570	0.022328
10	6	0	-3.975271	-0.048937	0.001079
11	6	0	-3.363171	-1.314172	-0.036549
12	6	0	-1.972231	-1.400183	-0.052833
13	8	0	-5.317572	0.130770	0.020395
14	6	0	-6.179899	-1.016385	0.006917
15	8	0	-1.384962	-2.612802	-0.089466
16	8	0	0.891417	-1.374861	-0.083088
17	8	0	3.007303	0.375012	1.137631
18	6	0	3.740325	-0.713314	0.843372
19	6	0	3.796134	-0.950516	-0.669523
20	6	0	3.155238	0.299894	-1.265312
21	8	0	4.288929	-1.376920	1.700190
22	8	0	5.140476	-1.092102	-1.116656
23	1	0	2.826902	2.053816	-0.010282
24	1	0	-0.013869	4.254773	0.314726
25	1	0	1.591814	3.668100	0.787367
26	1	0	1.184835	3.850774	-0.926119
27	1	0	-3.696490	2.101800	0.053128
28	1	0	-3.938410	-2.230375	-0.052755
29	1	0	-7.193253	-0.617444	0.029047
30	1	0	-6.034335	-1.603607	-0.905037
31	1	0	-6.010435	-1.642445	0.888414
32	1	0	-0.400629	-2.447251	-0.094897
33	1	0	3.214180	-1.851167	-0.886031
34	1	0	3.940268	0.936367	-1.681752
35	1	0	2.442518	0.069148	-2.057185
36	1	0	5.501153	-1.912461	-0.748565

1D:

Center Number	Atomic Number	Atomic Type	Coordinates (Angstroms) X	Y	Z
1	6	0	1.773359	-0.646989	-0.068860
2	6	0	1.456777	0.710819	-0.202031
3	6	0	0.086144	1.109326	-0.411299
4	6	0	-0.917466	0.050407	-0.464254
5	6	0	-0.523844	-1.253198	-0.342696
6	8	0	0.784165	-1.585841	-0.149703
7	6	0	-2.324951	0.575537	-0.681053
8	6	0	-1.353328	-2.502983	-0.373474
9	6	0	3.069267	-1.113181	0.140791
10	6	0	4.091758	-0.157964	0.218846
11	6	0	3.823883	1.220153	0.087283
12	6	0	2.524488	1.654523	-0.121497
13	8	0	5.393869	-0.470681	0.422262
14	6	0	5.765336	-1.847997	0.575657
15	8	0	2.264248	2.972797	-0.247477
16	8	0	-0.234663	2.325116	-0.540122
17	8	0	-3.263382	-0.455437	-1.112141
18	6	0	-4.230180	-0.668360	-0.194258
19	6	0	-3.949340	0.155730	1.065004
20	6	0	-2.984960	1.225405	0.564840
21	8	0	-5.158551	-1.422031	-0.390592
22	8	0	-5.123335	0.726993	1.610626
23	1	0	-2.280974	1.291877	-1.502524
24	1	0	-0.694310	-3.362480	-0.505500
25	1	0	-1.891782	-2.635158	0.571474
26	1	0	-2.083505	-2.478857	-1.180818
27	1	0	3.245355	-2.175655	0.235207
28	1	0	4.637319	1.933705	0.151232
29	1	0	6.843572	-1.843157	0.730211
30	1	0	5.269761	-2.291070	1.445288
31	1	0	5.524595	-2.420094	-0.325931
32	1	0	1.280155	3.051741	-0.387585
33	1	0	-3.468357	-0.512428	1.794117
34	1	0	-3.560894	2.104668	0.261796
35	1	0	-2.251068	1.528985	1.310287
36	1	0	-5.710330	0.013222	1.901916

1E:

Center Number	Atomic Number	Atomic Type	Coordinates (Angstroms) X	Y	Z
1	6	0	1.866168	-0.626660	0.046856
2	6	0	1.483029	0.717301	-0.057331
3	6	0	0.081825	1.072337	-0.074975
4	6	0	-0.877833	-0.027113	0.023733
5	6	0	-0.415953	-1.310337	0.121965
6	8	0	0.910313	-1.602991	0.127387
7	6	0	-2.333931	0.360087	0.018730
8	6	0	-1.205161	-2.574496	0.234300
9	6	0	3.193251	-1.045961	0.071475
10	6	0	4.183041	-0.056620	-0.015217
11	6	0	3.849906	1.308274	-0.123222
12	6	0	2.518882	1.695810	-0.144616
13	8	0	5.510614	-0.323153	-0.002758
14	6	0	5.949214	-1.684725	0.110114
15	8	0	2.200643	3.002702	-0.248072
16	8	0	-0.284404	2.276830	-0.166139
17	8	0	-3.079739	-0.368204	-1.026415
18	6	0	-4.361253	-0.560303	-0.648850
19	6	0	-4.579600	-0.020298	0.776601
20	6	0	-3.155705	0.120623	1.299648
21	8	0	-5.186517	-1.061474	-1.383939
22	8	0	-5.187098	1.270748	0.694044
23	1	0	-2.378827	1.413611	-0.260910
24	1	0	-1.067884	-3.008541	1.231125
25	1	0	-2.264718	-2.417159	0.055545
26	1	0	-0.832176	-3.300188	-0.494863
27	1	0	3.417163	-2.100437	0.154276
28	1	0	4.637512	2.050079	-0.188432
29	1	0	7.037529	-1.641832	0.096063
30	1	0	5.609550	-2.126871	1.052006
31	1	0	5.592486	-2.281024	-0.735522
32	1	0	1.204260	3.048471	-0.244012
33	1	0	-5.198223	-0.710811	1.357036
34	1	0	-3.055257	0.944365	2.009077
35	1	0	-2.847680	-0.803089	1.795815
36	1	0	-6.099497	1.170250	0.384661

1F:

Center Number	Atomic Number	Atomic Type	Coordinates (Angstroms) X	Y	Z
1	6	0	-1.834740	1.015773	0.008457
2	6	0	-1.164315	-0.220003	-0.034012
3	6	0	0.278725	-0.258263	-0.055135
4	6	0	0.975244	1.023802	-0.033999
5	6	0	0.246932	2.180799	0.022599
6	8	0	-1.111678	2.179261	0.036513
7	6	0	2.482424	1.027783	-0.073398
8	6	0	0.734792	3.594961	0.055377
9	6	0	-3.214605	1.123765	0.027479
10	6	0	-3.967715	-0.062858	0.002970
11	6	0	-3.347664	-1.324096	-0.039715
12	6	0	-1.956280	-1.400768	-0.057674
13	8	0	-5.310972	0.108103	0.024153
14	6	0	-6.166210	-1.044318	0.005300
15	8	0	-1.361220	-2.609297	-0.099004
16	8	0	0.905882	-1.353550	-0.090290
17	8	0	3.000459	0.369689	1.136588
18	6	0	3.726425	-0.723837	0.844587
19	6	0	3.797936	-0.954487	-0.668792
20	6	0	3.150830	0.291497	-1.267350
21	8	0	4.261601	-1.395162	1.703942
22	8	0	5.149379	-1.079130	-1.101415
23	1	0	2.851346	2.047941	-0.012520
24	1	0	1.808669	3.671968	0.208579
25	1	0	0.480860	4.092711	-0.887089
26	1	0	0.225146	4.132695	0.860330
27	1	0	-3.703326	2.089581	0.061889
28	1	0	-3.916999	-2.243920	-0.058579
29	1	0	-7.181997	-0.651739	0.029305
30	1	0	-6.016955	-1.626239	-0.909441
31	1	0	-5.992829	-1.673439	0.883830
32	1	0	-0.378282	-2.437642	-0.104666
33	1	0	3.229764	-1.861210	-0.895589
34	1	0	3.930538	0.925439	-1.697252
35	1	0	2.430793	0.053103	-2.050173
36	1	0	5.514989	-1.897051	-0.732684

1G:

Center Number	Atomic Number	Atomic Type	Coordinates (Angstroms) X	Y	Z
1	6	0	-1.914098	0.709211	0.003747
2	6	0	-1.133864	-0.454677	-0.032568
3	6	0	0.306876	-0.356340	-0.050487
4	6	0	0.879135	0.985737	-0.034979
5	6	0	0.045174	2.068894	0.018651
6	8	0	-1.307178	1.935527	0.031731
7	6	0	2.379197	1.129706	-0.081187
8	6	0	0.396554	3.523088	0.048768
9	6	0	-3.305881	0.697416	0.017783
10	6	0	-3.939580	-0.553160	-0.007033
11	6	0	-3.199697	-1.752081	-0.043465
12	6	0	-1.814266	-1.709286	-0.055630
13	8	0	-5.284448	-0.710106	0.001935
14	6	0	-6.123510	0.453530	0.036574
15	8	0	-1.105503	-2.856902	-0.091347
16	8	0	1.034817	-1.387289	-0.079540
17	8	0	2.960408	0.534371	1.132236
18	6	0	3.787717	-0.486471	0.847027
19	6	0	3.875706	-0.723967	-0.664458
20	6	0	3.105304	0.445154	-1.272227
21	8	0	4.388439	-1.094739	1.709955
22	8	0	5.231467	-0.717386	-1.101714
23	1	0	2.653549	2.180169	-0.033025
24	1	0	-0.178374	4.015702	0.838275
25	1	0	1.455074	3.700469	0.224657
26	1	0	0.118577	3.989249	-0.903050
27	1	0	-3.847280	1.632792	0.047211
28	1	0	-3.718989	-2.703333	-0.061680
29	1	0	-7.145079	0.075604	0.036681
30	1	0	-5.945137	1.035812	0.945987
31	1	0	-5.960780	1.077740	-0.847612
32	1	0	-0.144275	-2.593510	-0.094220
33	1	0	3.400189	-1.685330	-0.879588
34	1	0	3.815400	1.145624	-1.719150
35	1	0	2.404554	0.127095	-2.044095
36	1	0	5.679959	-1.486403	-0.719546

Table S5. Cartesian coordinates for the low-energy optimized conformers of (3′*R*, 5′*R*)-**1** at B3LYP/6 – 31+g (d,p) level of theory in MeOH.

1AA:

Center Number	Atomic Number	Atomic Type	Coordinates (Angstroms)		
			X	Y	Z
1	6	0	-1.754581	-0.865357	0.023434
2	6	0	-1.534006	0.522509	-0.033590
3	6	0	-0.186477	1.042177	-0.066695
4	6	0	0.898739	0.061757	-0.031864
5	6	0	0.595383	-1.270609	0.022945
6	8	0	-0.684819	-1.722122	0.042620
7	6	0	2.296736	0.620374	-0.052170
8	6	0	1.532499	-2.434099	0.066216
9	6	0	-3.019011	-1.427494	0.060016
10	6	0	-4.125181	-0.560395	0.038437
11	6	0	-3.961035	0.834408	-0.019744
12	6	0	-2.675029	1.370870	-0.055490
13	8	0	-5.334414	-1.169015	0.077508
14	6	0	-6.525487	-0.368360	0.061995
15	8	0	-2.519152	2.708155	-0.110473
16	8	0	0.032810	2.284479	-0.116229
17	8	0	3.096307	0.027304	-1.147178
18	6	0	4.398320	-0.015717	-0.798504
19	6	0	4.580637	0.552167	0.614543
20	6	0	3.175556	0.438672	1.198044
21	8	0	5.260844	-0.456575	-1.528959
22	8	0	5.533491	-0.163132	1.374122
23	1	0	2.206592	1.682485	-0.287676
24	1	0	2.566677	-2.140956	-0.088141
25	1	0	1.442198	-2.942687	1.032503
26	1	0	1.253445	-3.152203	-0.711129
27	1	0	-3.156693	-2.500788	0.103843
28	1	0	-4.804284	1.511853	-0.037463
29	1	0	-7.351519	-1.077382	0.100967
30	1	0	-6.564349	0.291364	0.934468
31	1	0	-6.588541	0.220657	-0.858264
32	1	0	-1.534775	2.873870	-0.126863
33	1	0	4.873116	1.607902	0.505815
34	1	0	3.045932	-0.553465	1.640932
35	1	0	2.952978	1.192280	1.955169
36	1	0	6.396304	-0.091100	0.939111

1BB:

Center Number	Atomic Number	Atomic Type	Coordinates (Angstroms) X	Y	Z
1	6	0	-1.843067	-0.627053	0.007973
2	6	0	-1.539092	0.740676	-0.028857
3	6	0	-0.161105	1.176378	-0.051493
4	6	0	0.860696	0.130060	-0.028029
5	6	0	0.475051	-1.181458	0.004396
6	8	0	-0.831751	-1.549796	0.015654
7	6	0	2.291835	0.598671	-0.033217
8	6	0	1.337491	-2.401647	0.030544
9	6	0	-3.143227	-1.122957	0.034797
10	6	0	-4.189156	-0.189191	0.022507
11	6	0	-3.936437	1.196473	-0.016452
12	6	0	-2.630339	1.660972	-0.042225
13	8	0	-5.498576	-0.533054	0.046518
14	6	0	-5.855028	-1.922375	0.090290
15	8	0	-2.389207	2.987766	-0.079212
16	8	0	0.134743	2.402985	-0.083527
17	8	0	3.051649	-0.012274	-1.146470
18	6	0	4.347313	-0.153806	-0.800515
19	6	0	4.565925	0.351648	0.630730
20	6	0	3.155077	0.322013	1.210566
21	8	0	5.179307	-0.626700	-1.546149
22	8	0	5.460760	-0.458507	1.365855
23	1	0	2.270780	1.670781	-0.237142
24	1	0	0.991168	-3.105813	-0.732062
25	1	0	2.382375	-2.173061	-0.157516
26	1	0	1.246173	-2.897749	1.003371
27	1	0	-3.306064	-2.191343	0.063066
28	1	0	-4.766073	1.893955	-0.025346
29	1	0	-6.943990	-1.943041	0.103068
30	1	0	-5.486225	-2.447891	-0.796135
31	1	0	-5.466257	-2.396222	0.997122
32	1	0	-1.397376	3.091145	-0.091221
33	1	0	4.936813	1.385309	0.558738
34	1	0	2.955541	-0.669908	1.626531
35	1	0	2.985115	1.068670	1.987958
36	1	0	6.326989	-0.438616	0.932128

Table S6. Cartesian coordinates for the low-energy optimized conformers of 14*S*-**5** at B3LYP/6－31+g (d,p) level of theory in MeOH.

5A:

Center	Atomic	Atomic	Coordinates (Angstroms)		
Number	Number	Type	X	Y	Z
1	8	0	4.049994	3.244841	0.050126
2	6	0	5.296592	-0.134494	-0.689814
3	6	0	4.369161	-0.904772	0.021069
4	6	0	3.341800	-0.268302	0.729011
5	6	0	3.248731	1.127319	0.727030
6	6	0	4.186431	1.881320	0.012318
7	6	0	5.214815	1.261012	-0.701856
8	8	0	6.324900	-0.698076	-1.399550
9	6	0	2.316254	-1.090211	1.498812
10	6	0	1.013824	-1.243206	0.757393
11	6	0	-0.173138	-0.701838	1.183414
12	8	0	1.103417	-1.960397	-0.364561
13	6	0	-1.394844	-0.875555	0.439488
14	6	0	-2.623290	-0.287833	0.915462
15	8	0	-1.391110	-1.557475	-0.657078
16	6	0	-3.804526	-0.441696	0.237461
17	6	0	-5.111584	0.155822	0.663979
18	8	0	-3.870692	-1.146290	-0.903641
19	6	0	-5.725853	1.105841	-0.392596
20	6	0	-6.938623	1.843283	0.159417
21	8	0	-6.160086	0.388024	-1.557389
22	1	0	4.750829	3.668000	-0.466282
23	1	0	4.448467	-1.988286	0.023398
24	1	0	2.463065	1.637622	1.275118
25	1	0	5.947654	1.839755	-1.255983
26	1	0	6.289216	-1.662862	-1.330057
27	1	0	2.715997	-2.093178	1.686099
28	1	0	2.114478	-0.625855	2.467156
29	1	0	-0.192571	-0.128831	2.101811
30	1	0	0.179783	-1.984030	-0.756487
31	1	0	-2.611660	0.289645	1.831222
32	1	0	-5.830264	-0.652387	0.854318
33	1	0	-4.963044	0.701749	1.599233
34	1	0	-2.933692	-1.468408	-1.090620
35	1	0	-4.960709	1.839741	-0.687387
36	1	0	-7.701040	1.133372	0.498386
37	1	0	-7.376373	2.477181	-0.617581
38	1	0	-6.653181	2.479556	1.002547
39	1	0	-5.432832	-0.187957	-1.841684

5B:

Center Number	Atomic Number	Atomic Type	Coordinates (Angstroms) X	Y	Z
1	8	0	-6.562755	-1.368102	-0.619345
2	6	0	-4.524546	1.626264	-0.299573
3	6	0	-3.420497	1.155562	0.419789
4	6	0	-3.365732	-0.190447	0.797236
5	6	0	-4.411779	-1.057299	0.458192
6	6	0	-5.508528	-0.568641	-0.261437
7	6	0	-5.575490	0.772989	-0.646957
8	8	0	-4.527280	2.953888	-0.641123
9	6	0	-2.165170	-0.709156	1.576776
10	6	0	-0.919672	-0.823579	0.737986
11	6	0	0.237921	-0.133393	0.995957
12	8	0	-1.025068	-1.672804	-0.286354
13	6	0	1.412864	-0.291259	0.176863
14	6	0	2.618088	0.436213	0.491303
15	8	0	1.389464	-1.088915	-0.838266
16	6	0	3.752658	0.311108	-0.267550
17	6	0	5.041499	1.023795	0.011690
18	8	0	3.794338	-0.498489	-1.337781
19	6	0	6.234729	0.066112	0.245182
20	6	0	7.467633	0.817950	0.728703
21	8	0	6.602357	-0.619881	-0.961189
22	1	0	-6.417259	-2.272402	-0.306057
23	1	0	-2.622092	1.844128	0.677811
24	1	0	-4.375952	-2.102753	0.751848
25	1	0	-6.435827	1.133490	-1.202865
26	1	0	-5.334873	3.174433	-1.127034
27	1	0	-1.954617	-0.054246	2.426432
28	1	0	-2.392666	-1.704419	1.975614
29	1	0	0.272998	0.541671	1.841671
30	1	0	-0.137260	-1.660102	-0.753753
31	1	0	2.627124	1.096925	1.349025
32	1	0	5.285852	1.670680	-0.841401
33	1	0	4.910001	1.660427	0.890361
34	1	0	2.884700	-0.928767	-1.401117
35	1	0	5.943494	-0.674519	1.005212
36	1	0	7.755672	1.591966	0.008960
37	1	0	8.305914	0.124544	0.845843
38	1	0	7.275955	1.292633	1.695717
39	1	0	5.796516	-0.996590	-1.348764

5C:

Center Number	Atomic Number	Atomic Type	Coordinates (Angstroms) X	Y	Z
1	8	0	-4.136711	-2.290647	-2.186729
2	6	0	-4.366108	-0.913579	1.215816
3	6	0	-3.755352	0.329965	1.023186
4	6	0	-3.275252	0.676213	-0.247794
5	6	0	-3.417034	-0.215533	-1.315247
6	6	0	-4.028795	-1.457397	-1.104002
7	6	0	-4.508485	-1.816833	0.157637
8	8	0	-4.857574	-1.307810	2.433000
9	6	0	-2.608507	2.017664	-0.462476
10	6	0	-1.108548	2.051898	-0.303968
11	6	0	-0.306674	0.974931	-0.023979
12	8	0	-0.607865	3.278826	-0.474718
13	6	0	1.122258	1.108554	0.094396
14	6	0	1.936112	-0.049399	0.375338
15	8	0	1.680853	2.263948	-0.054997
16	6	0	3.298253	0.040376	0.496516
17	6	0	4.204769	-1.122900	0.766682
18	8	0	3.938324	1.212754	0.357563
19	6	0	5.271347	-1.337479	-0.334211
20	6	0	6.031230	-2.641205	-0.128679
21	8	0	6.240438	-0.278455	-0.342085
22	1	0	-4.571008	-3.118104	-1.934368
23	1	0	-3.654655	1.022192	1.854991
24	1	0	-3.058104	0.038188	-2.307679
25	1	0	-4.990877	-2.774634	0.328013
26	1	0	-4.711645	-0.614432	3.092550
27	1	0	-2.828097	2.394382	-1.469587
28	1	0	-3.018284	2.762125	0.231275
29	1	0	-0.756295	-0.000210	0.105015
30	1	0	0.386034	3.194993	-0.356381
31	1	0	1.458301	-1.013959	0.491837
32	1	0	4.726086	-0.957873	1.719074
33	1	0	3.599675	-2.027728	0.866440
34	1	0	3.227155	1.899195	0.157050
35	1	0	4.763441	-1.366592	-1.310083
36	1	0	6.515192	-2.656014	0.854040
37	1	0	6.803283	-2.748958	-0.896522
38	1	0	5.354332	-3.497991	-0.198575
39	1	0	5.760458	0.564842	-0.338654

5D:

Center Number	Atomic Number	Atomic Type	Coordinates (Angstroms) X	Y	Z
1	8	0	-6.328667	-0.675779	-1.347448
2	6	0	-4.118847	1.809897	0.122121
3	6	0	-3.158145	1.012600	0.754288
4	6	0	-3.259638	-0.380577	0.679735
5	6	0	-4.318539	-0.970813	-0.021332
6	6	0	-5.269548	-0.157524	-0.648509
7	6	0	-5.179433	1.235850	-0.584056
8	8	0	-3.971737	3.168453	0.231270
9	6	0	-2.211780	-1.251277	1.359871
10	6	0	-0.903043	-1.287528	0.615292
11	6	0	0.279866	-0.822573	1.132432
12	8	0	-0.980687	-1.824514	-0.604184
13	6	0	1.511374	-0.890270	0.387295
14	6	0	2.736893	-0.399299	0.968528
15	8	0	1.519454	-1.392402	-0.802280
16	6	0	3.930085	-0.467684	0.297764
17	6	0	5.241627	0.010905	0.845248
18	8	0	4.010633	-0.986296	-0.938121
19	6	0	5.968612	1.047343	-0.048770
20	6	0	5.129845	2.291777	-0.342943
21	8	0	6.455863	0.447156	-1.259147
22	1	0	-6.296374	-1.643160	-1.334994
23	1	0	-2.347873	1.487960	1.298023
24	1	0	-4.403966	-2.052309	-0.078469
25	1	0	-5.929718	1.848393	-1.075022
26	1	0	-4.689622	3.623408	-0.231884
27	1	0	-2.025224	-0.892741	2.375444
28	1	0	-2.583745	-2.279465	1.434542
29	1	0	0.290131	-0.399465	2.129038
30	1	0	-0.052577	-1.791599	-0.984475
31	1	0	2.714911	0.028595	1.962860
32	1	0	5.079787	0.440233	1.837529
33	1	0	5.905849	-0.854923	0.961511
34	1	0	3.073759	-1.258230	-1.191065
35	1	0	6.871463	1.347063	0.492896
36	1	0	4.229309	2.041267	-0.914548
37	1	0	5.717840	3.005283	-0.927449
38	1	0	4.819478	2.780717	0.586894
39	1	0	5.711735	-0.008944	-1.683344

5E:

Center Number	Atomic Number	Atomic Type	Coordinates (Angstroms) X	Y	Z
1	8	0	4.685078	2.921623	-0.311469
2	6	0	5.349832	-0.693896	-0.380890
3	6	0	4.233065	-1.166593	0.317604
4	6	0	3.278695	-0.257733	0.791461
5	6	0	3.446433	1.113305	0.571047
6	6	0	4.570418	1.568000	-0.127959
7	6	0	5.529412	0.673349	-0.610283
8	8	0	6.316906	-1.534489	-0.867637
9	6	0	2.051105	-0.756801	1.540493
10	6	0	0.809802	-0.785937	0.687249
11	6	0	-0.320720	-0.058510	0.963280
12	8	0	0.888379	-1.598099	-0.368775
13	6	0	-1.494145	-0.138168	0.130712
14	6	0	-2.673609	0.621610	0.466265
15	8	0	-1.493419	-0.896452	-0.914476
16	6	0	-3.807802	0.567582	-0.301394
17	6	0	-5.068672	1.325175	-0.010376
18	8	0	-3.874081	-0.198982	-1.401747
19	6	0	-6.326903	0.432630	0.140203
20	6	0	-6.184123	-0.639951	1.221056
21	8	0	-6.717956	-0.140741	-1.117054
22	1	0	5.495084	3.127473	-0.799616
23	1	0	4.109614	-2.232196	0.489194
24	1	0	2.720823	1.833212	0.936167
25	1	0	6.405147	1.020212	-1.150734
26	1	0	6.094822	-2.453614	-0.660318
27	1	0	2.232524	-1.776223	1.900472
28	1	0	1.859371	-0.127568	2.413237
29	1	0	-0.335930	0.584463	1.834151
30	1	0	0.005056	-1.529333	-0.840780
31	1	0	-2.663701	1.248254	1.349220
32	1	0	-4.931725	1.912103	0.901606
33	1	0	-5.251769	2.026643	-0.834117
34	1	0	-2.981087	-0.659545	-1.478119
35	1	0	-7.152799	1.099492	0.407731
36	1	0	-5.387089	-1.350448	0.975486
37	1	0	-7.120796	-1.196475	1.317592
38	1	0	-5.950632	-0.186348	2.190465
39	1	0	-5.942907	-0.587370	-1.493179

5F:

Center Number	Atomic Number	Atomic Type	Coordinates (Angstroms) X	Y	Z
1	8	0	4.753181	-1.047964	2.530461
2	6	0	4.081683	-1.388252	-1.026263
3	6	0	3.410341	-0.193337	-1.314451
4	6	0	3.176974	0.732417	-0.293240
5	6	0	3.623526	0.466966	1.009309
6	6	0	4.292086	-0.731379	1.279236
7	6	0	4.527692	-1.667753	0.267229
8	8	0	4.281339	-2.257509	-2.066913
9	6	0	2.449219	2.025001	-0.592806
10	6	0	0.951286	2.006783	-0.414379
11	6	0	0.201711	0.922333	-0.036200
12	8	0	0.394662	3.193580	-0.673108
13	6	0	-1.229488	1.005231	0.101283
14	6	0	-1.985273	-0.157092	0.500936
15	8	0	-1.840926	2.118755	-0.133045
16	6	0	-3.346046	-0.113822	0.656420
17	6	0	-4.191138	-1.278735	1.077530
18	8	0	-4.042156	1.012961	0.433798
19	6	0	-5.326975	-1.635345	0.085089
20	6	0	-4.825613	-1.936806	-1.327998
21	8	0	-6.350075	-0.627560	0.072774
22	1	0	4.539288	-0.339207	3.154010
23	1	0	3.078975	-0.001390	-2.330034
24	1	0	3.452004	1.186744	1.805451
25	1	0	5.054580	-2.588869	0.497418
26	1	0	4.755423	-3.044676	-1.762541
27	1	0	2.833887	2.834007	0.040846
28	1	0	2.640828	2.337985	-1.626856
29	1	0	0.696092	-0.018680	0.163525
30	1	0	-0.592922	3.076975	-0.531091
31	1	0	-1.462918	-1.087094	0.686812
32	1	0	-3.550593	-2.152897	1.221344
33	1	0	-4.653313	-1.041598	2.044203
34	1	0	-3.368944	1.708212	0.152852
35	1	0	-5.822017	-2.526268	0.484533
36	1	0	-4.353089	-1.057729	-1.780084
37	1	0	-5.663617	-2.238934	-1.962739
38	1	0	-4.091729	-2.749979	-1.314860
39	1	0	-5.920045	0.228033	-0.084704

5G:

Center Number	Atomic Number	Atomic Type	Coordinates (Angstroms) X	Y	Z
1	8	0	4.715573	-1.037570	2.540210
2	6	0	4.041767	-1.362385	-1.060605
3	6	0	3.386743	-0.150614	-1.302034
4	6	0	3.178154	0.746714	-0.244803
5	6	0	3.634991	0.433048	1.038571
6	6	0	4.290646	-0.784285	1.262070
7	6	0	4.500053	-1.689784	0.219725
8	8	0	4.273429	-2.280493	-2.051482
9	6	0	2.461379	2.055479	-0.496869
10	6	0	0.958869	2.030025	-0.361887
11	6	0	0.206850	0.947959	0.018242
12	8	0	0.402018	3.209266	-0.652654
13	6	0	-1.227209	1.025053	0.124828
14	6	0	-1.984092	-0.133768	0.532751
15	8	0	-1.840028	2.129961	-0.143973
16	6	0	-3.347731	-0.095781	0.661224
17	6	0	-4.193916	-1.256373	1.091986
18	8	0	-4.046144	1.021680	0.401326
19	6	0	-5.302753	-1.648138	0.082439
20	6	0	-4.764842	-1.988064	-1.308257
21	8	0	-6.330050	-0.646857	0.015113
22	1	0	5.156843	-1.897988	2.584771
23	1	0	3.040964	0.091732	-2.303555
24	1	0	3.488768	1.118355	1.867414
25	1	0	5.011820	-2.633169	0.384368
26	1	0	3.909951	-1.964172	-2.891055
27	1	0	2.828694	2.828411	0.190041
28	1	0	2.682458	2.422971	-1.506684
29	1	0	0.701914	0.013749	0.246083
30	1	0	-0.587659	3.090103	-0.529124
31	1	0	-1.460097	-1.056379	0.748424
32	1	0	-3.550337	-2.121120	1.274156
33	1	0	-4.680946	-0.997693	2.040764
34	1	0	-3.371851	1.715450	0.119522
35	1	0	-5.803264	-2.529820	0.495399
36	1	0	-4.281393	-1.121382	-1.772569
37	1	0	-5.586124	-2.309027	-1.955524
38	1	0	-4.031093	-2.799750	-1.253819
39	1	0	-5.900495	0.207314	-0.151203

5H:

Center Number	Atomic Number	Atomic Type	Coordinates (Angstroms) X	Y	Z
1	8	0	4.504456	3.044081	-0.214651
2	6	0	5.446067	-0.508545	-0.318710
3	6	0	4.349946	-1.077170	0.340308
4	6	0	3.316700	-0.251672	0.801129
5	6	0	3.384794	1.131777	0.605810
6	6	0	4.488926	1.682744	-0.054109
7	6	0	5.526574	0.872102	-0.521953
8	8	0	6.488074	-1.263610	-0.791094
9	6	0	2.111334	-0.855238	1.509162
10	6	0	0.890367	-0.929485	0.629938
11	6	0	-0.271949	-0.246224	0.881294
12	8	0	1.025744	-1.731552	-0.429414
13	6	0	-1.419025	-0.356580	0.013388
14	6	0	-2.626043	0.369606	0.313678
15	8	0	-1.361867	-1.109709	-1.034483
16	6	0	-3.734447	0.293208	-0.492377
17	6	0	-5.006054	1.049941	-0.243714
18	8	0	-3.752896	-0.461247	-1.594937
19	6	0	-6.242954	0.151316	-0.103709
20	6	0	-7.523603	0.970057	0.043036
21	8	0	-6.016074	-0.679537	1.049847
22	1	0	5.308942	3.318958	-0.677347
23	1	0	4.302950	-2.152013	0.491871
24	1	0	2.596148	1.787822	0.960397
25	1	0	6.387918	1.293909	-1.031269
26	1	0	6.332024	-2.200955	-0.606690
27	1	0	2.351880	-1.872994	1.836890
28	1	0	1.865181	-0.270176	2.398992
29	1	0	-0.332460	0.390389	1.755018
30	1	0	0.151143	-1.690185	-0.922164
31	1	0	-2.659876	0.993248	1.198162
32	1	0	-4.897131	1.663981	0.654896
33	1	0	-5.170597	1.724645	-1.093220
34	1	0	-2.849913	-0.898093	-1.649352
35	1	0	-6.316573	-0.486886	-0.994119
36	1	0	-7.463549	1.632059	0.913728
37	1	0	-8.388122	0.309339	0.169963
38	1	0	-7.697099	1.580374	-0.849680
39	1	0	-6.764154	-1.285855	1.142751

5I:

Center Number	Atomic Number	Atomic Type	Coordinates (Angstroms) X	Y	Z
1	8	0	-6.506650	-0.605162	-1.070476
2	6	0	-4.097703	1.874751	0.056372
3	6	0	-3.130123	1.081518	0.682886
4	6	0	-3.294936	-0.307135	0.721651
5	6	0	-4.423755	-0.896031	0.138727
6	6	0	-5.381268	-0.086874	-0.484083
7	6	0	-5.228455	1.301562	-0.532043
8	8	0	-3.885532	3.229118	0.050431
9	6	0	-2.240657	-1.174630	1.395831
10	6	0	-0.969307	-1.285085	0.595677
11	6	0	0.250162	-0.840073	1.037909
12	8	0	-1.120766	-1.868598	-0.596323
13	6	0	1.443763	-0.978969	0.239245
14	6	0	2.708896	-0.508889	0.742001
15	8	0	1.375901	-1.527863	-0.927957
16	6	0	3.866610	-0.632380	0.015236
17	6	0	5.214006	-0.186412	0.501518
18	8	0	3.880144	-1.191197	-1.198489
19	6	0	5.882378	0.859037	-0.403254
20	6	0	7.291600	1.201325	0.075230
21	8	0	5.027162	2.016752	-0.389288
22	1	0	-6.513360	-1.569501	-0.986172
23	1	0	-2.264061	1.556589	1.132717
24	1	0	-4.557864	-1.973732	0.168047
25	1	0	-5.984206	1.911533	-1.017919
26	1	0	-4.611156	3.681810	-0.402685
27	1	0	-1.997576	-0.773817	2.383434
28	1	0	-2.637274	-2.186372	1.538129
29	1	0	0.320785	-0.377050	2.014158
30	1	0	-0.208888	-1.879606	-1.017577
31	1	0	2.748617	-0.047408	1.720677
32	1	0	5.123556	0.213952	1.515480
33	1	0	5.868007	-1.066221	0.544909
34	1	0	2.930367	-1.451367	-1.396498
35	1	0	5.929058	0.461688	-1.425748
36	1	0	7.270906	1.579357	1.103141
37	1	0	7.738807	1.968258	-0.566377
38	1	0	7.937361	0.317386	0.040117
39	1	0	5.397589	2.679811	-0.988434

5J:

Center Number	Atomic Number	Atomic Type	Coordinates (Angstroms) X	Y	Z
1	8	0	6.492524	-1.289098	-0.654402
2	6	0	4.471828	1.674591	-0.058994
3	6	0	3.346694	1.140680	0.579122
4	6	0	3.271785	-0.237316	0.807555
5	6	0	4.318491	-1.074158	0.400993
6	6	0	5.435977	-0.522473	-0.236278
7	6	0	5.523434	0.852461	-0.472615
8	8	0	4.493683	3.031375	-0.253286
9	6	0	2.045879	-0.823265	1.494779
10	6	0	0.846976	-0.907753	0.586986
11	6	0	-0.320440	-0.219291	0.800917
12	8	0	1.006121	-1.724331	-0.456864
13	6	0	-1.445330	-0.341529	-0.092127
14	6	0	-2.659607	0.392203	0.170253
15	8	0	-1.367482	-1.108854	-1.126626
16	6	0	-3.752431	0.301880	-0.655054
17	6	0	-5.047426	1.016365	-0.413112
18	8	0	-3.754246	-0.475012	-1.744189
19	6	0	-6.176068	0.075715	0.074117
20	6	0	-7.504148	0.811385	0.185847
21	8	0	-5.883125	-0.455596	1.376550
22	1	0	6.332569	-2.220880	-0.446481
23	1	0	2.547951	1.805745	0.891840
24	1	0	4.265964	-2.144584	0.579510
25	1	0	6.400706	1.261506	-0.964938
26	1	0	5.313790	3.294515	-0.694879
27	1	0	2.273384	-1.836331	1.845510
28	1	0	1.780202	-0.222466	2.368348
29	1	0	-0.401191	0.429354	1.664088
30	1	0	0.144233	-1.690389	-0.971523
31	1	0	-2.708105	1.038025	1.038082
32	1	0	-4.902309	1.808467	0.327038
33	1	0	-5.365834	1.476757	-1.355794
34	1	0	-2.850859	-0.910787	-1.778831
35	1	0	-6.275448	-0.753244	-0.639704
36	1	0	-7.423863	1.648047	0.888351
37	1	0	-8.282933	0.131404	0.543325
38	1	0	-7.808743	1.203471	-0.789096
39	1	0	-5.072998	-0.983438	1.331351

5K:

Center Number	Atomic Number	Atomic Type	Coordinates (Angstroms) X	Y	Z
1	8	0	-4.539245	2.934752	-0.351330
2	6	0	-5.404309	-0.636775	-0.241101
3	6	0	-4.271466	-1.148375	0.402742
4	6	0	-3.241393	-0.279586	0.783621
5	6	0	-3.347803	1.090558	0.521987
6	6	0	-4.487177	1.584019	-0.123086
7	6	0	-5.523075	0.729550	-0.510395
8	8	0	-6.445607	-1.436749	-0.634377
9	6	0	-2.001690	-0.821568	1.481718
10	6	0	-0.784630	-0.840431	0.594421
11	6	0	0.358761	-0.129417	0.854890
12	8	0	-0.901459	-1.624545	-0.480699
13	6	0	1.505515	-0.190340	-0.018892
14	6	0	2.693271	0.560732	0.294086
15	8	0	1.463941	-0.921629	-1.083070
16	6	0	3.804736	0.528800	-0.512167
17	6	0	5.060657	1.302383	-0.230749
18	8	0	3.834509	-0.195438	-1.635283
19	6	0	6.247005	0.480563	0.317734
20	6	0	6.773859	-0.604557	-0.619126
21	8	0	5.820090	-0.073521	1.574878
22	1	0	-5.366015	3.168732	-0.796847
23	1	0	-4.194959	-2.213014	0.605929
24	1	0	-2.562152	1.780359	0.813671
25	1	0	-6.411469	1.107300	-1.007791
26	1	0	-6.263830	-2.359011	-0.403353
27	1	0	-1.776648	-0.223720	2.368929
28	1	0	-2.191140	-1.848497	1.814610
29	1	0	0.405064	0.490805	1.741173
30	1	0	-0.030466	-1.547356	-0.975637
31	1	0	2.711785	1.165734	1.191661
32	1	0	5.385690	1.783495	-1.160194
33	1	0	4.833698	2.089239	0.493176
34	1	0	2.941842	-0.652611	-1.696546
35	1	0	7.051757	1.205789	0.504349
36	1	0	6.011249	-1.361252	-0.820099
37	1	0	7.644356	-1.095238	-0.169483
38	1	0	7.090824	-0.169426	-1.572830
39	1	0	6.569034	-0.537629	1.974241

5L:

Center Number	Atomic Number	Atomic Type	Coordinates (Angstroms) X	Y	Z
1	8	0	-4.769356	-1.290041	2.464960
2	6	0	-4.039727	-1.403590	-1.139197
3	6	0	-3.432441	-0.155477	-1.309151
4	6	0	-3.273917	0.693447	-0.207224
5	6	0	-3.732253	0.296935	1.054507
6	6	0	-4.339029	-0.953949	1.208020
7	6	0	-4.499102	-1.814647	0.116966
8	8	0	-4.166407	-2.193651	-2.251816
9	6	0	-2.610174	2.042212	-0.382182
10	6	0	-1.107300	2.069143	-0.253633
11	6	0	-0.305786	0.989327	0.016607
12	8	0	-0.603724	3.293367	-0.434971
13	6	0	1.125110	1.117216	0.114683
14	6	0	1.937393	-0.043165	0.390561
15	8	0	1.686826	2.269277	-0.047694
16	6	0	3.301270	0.040701	0.494073
17	6	0	4.205554	-1.125217	0.760060
18	8	0	3.945277	1.209206	0.340406
19	6	0	5.255153	-1.353804	-0.354192
20	6	0	6.012133	-2.659205	-0.148476
21	8	0	6.228688	-0.299263	-0.385107
22	1	0	-5.173240	-2.169605	2.460448
23	1	0	-3.089503	0.138532	-2.296073
24	1	0	-3.624616	0.944794	1.918616
25	1	0	-4.976563	-2.783382	0.242524
26	1	0	-4.596047	-3.031164	-2.026497
27	1	0	-2.850188	2.459103	-1.368410
28	1	0	-3.004228	2.758433	0.349579
29	1	0	-0.757722	0.016716	0.156097
30	1	0	0.391438	3.205417	-0.331026
31	1	0	1.456658	-1.004811	0.518659
32	1	0	4.741189	-0.955751	1.703692
33	1	0	3.597379	-2.026161	0.875156
34	1	0	3.235075	1.898060	0.145511
35	1	0	4.733002	-1.388853	-1.322327
36	1	0	6.511257	-2.667153	0.826703
37	1	0	6.771549	-2.777909	-0.927220
38	1	0	5.330146	-3.513288	-0.199674
39	1	0	5.752702	0.546300	-0.380530

5M:

Center Number	Atomic Number	Atomic Type	Coordinates (Angstroms)		
			X	Y	Z
1	8	0	-3.802215	3.210718	-0.113099
2	6	0	-5.234362	-0.101150	0.595337
3	6	0	-4.301278	-0.920164	-0.051125
4	6	0	-3.209029	-0.338705	-0.706978
5	6	0	-3.057043	1.051790	-0.717475
6	6	0	-4.000432	1.855010	-0.067199
7	6	0	-5.094039	1.289602	0.594334
8	8	0	-6.323722	-0.611066	1.252939
9	6	0	-2.178228	-1.215402	-1.405420
10	6	0	-0.888120	-1.330204	-0.636136
11	6	0	0.317593	-0.866572	-1.096546
12	8	0	-1.008823	-1.934831	0.548941
13	6	0	1.528992	-1.006429	-0.324944
14	6	0	2.778364	-0.515023	-0.845000
15	8	0	1.490245	-1.577133	0.833486
16	6	0	3.953345	-0.637689	-0.144617
17	6	0	5.281369	-0.156346	-0.653785
18	8	0	3.995415	-1.225766	1.055064
19	6	0	5.802665	1.158550	-0.035455
20	6	0	6.058475	1.111589	1.469774
21	8	0	4.849897	2.181781	-0.374516
22	1	0	-4.508753	3.670951	0.361952
23	1	0	-4.425901	-1.999357	-0.042871
24	1	0	-2.219702	1.520645	-1.224582
25	1	0	-5.831457	1.906981	1.098710
26	1	0	-6.323763	-1.577659	1.200379
27	1	0	-2.584255	-2.225099	-1.534403
28	1	0	-1.957693	-0.817796	-2.399429
29	1	0	0.363369	-0.386625	-2.066095
30	1	0	-0.087641	-1.944819	0.949902
31	1	0	2.793105	-0.037696	-1.816608
32	1	0	6.026974	-0.936468	-0.463243
33	1	0	5.212257	-0.014436	-1.735424
34	1	0	3.051264	-1.497730	1.265924
35	1	0	6.751428	1.373478	-0.547207
36	1	0	5.136388	0.920435	2.024690
37	1	0	6.478398	2.065780	1.807168
38	1	0	6.779773	0.324375	1.713438
39	1	0	5.190199	3.032290	-0.063407

5N:

Center Number	Atomic Number	Atomic Type	Coordinates (Angstroms)		
			X	Y	Z
1	8	0	-4.839079	-1.371712	2.423324
2	6	0	-4.023319	-1.448552	-1.118510
3	6	0	-3.420753	-0.198778	-1.308775
4	6	0	-3.278835	0.673696	-0.222521
5	6	0	-3.752002	0.299363	1.040842
6	6	0	-4.355313	-0.953421	1.210947
7	6	0	-4.495196	-1.835440	0.137746
8	8	0	-4.186184	-2.344635	-2.142887
9	6	0	-2.618578	2.021648	-0.415281
10	6	0	-1.116470	2.055643	-0.277407
11	6	0	-0.312402	0.980598	0.003352
12	8	0	-0.617109	3.280855	-0.463342
13	6	0	1.117727	1.114449	0.106924
14	6	0	1.933227	-0.041582	0.390797
15	8	0	1.675405	2.267995	-0.058389
16	6	0	3.296492	0.047878	0.498613
17	6	0	4.204334	-1.113661	0.771449
18	8	0	3.936487	1.218194	0.342776
19	6	0	5.257995	-1.342020	-0.339106
20	6	0	6.018826	-2.644193	-0.127286
21	8	0	6.227964	-0.284252	-0.370315
22	1	0	-4.694832	-0.689215	3.094408
23	1	0	-3.063629	0.090192	-2.293839
24	1	0	-3.652020	0.975219	1.886136
25	1	0	-4.966506	-2.802286	0.276170
26	1	0	-3.820199	-1.984837	-2.963555
27	1	0	-2.853685	2.421811	-1.409678
28	1	0	-3.020012	2.748397	0.301836
29	1	0	-0.761201	0.006969	0.145877
30	1	0	0.377882	3.197589	-0.353664
31	1	0	1.455672	-1.004540	0.520871
32	1	0	4.736665	-0.938822	1.715951
33	1	0	3.599246	-2.016488	0.887976
34	1	0	3.224473	1.903625	0.142597
35	1	0	4.738770	-1.381761	-1.308635
36	1	0	6.514873	-2.647715	0.849487
37	1	0	6.781136	-2.762360	-0.903283
38	1	0	5.339996	-3.500787	-0.178323
39	1	0	5.749113	0.559687	-0.370366

5O:

Center Number	Atomic Number	Atomic Type	Coordinates (Angstroms) X	Y	Z
1	8	0	4.711654	-0.988396	2.550170
2	6	0	4.063495	-1.359279	-1.052606
3	6	0	3.396866	-0.157219	-1.310276
4	6	0	3.174286	0.748551	-0.266314
5	6	0	3.626099	0.454199	1.025564
6	6	0	4.291441	-0.751961	1.267280
7	6	0	4.517493	-1.667877	0.234194
8	8	0	4.253135	-2.208701	-2.111279
9	6	0	2.449699	2.049504	-0.536086
10	6	0	0.949413	2.024263	-0.380095
11	6	0	0.200357	0.938520	-0.004345
12	8	0	0.390315	3.207071	-0.651489
13	6	0	-1.232451	1.015740	0.117470
14	6	0	-1.986971	-0.147426	0.517388
15	8	0	-1.846636	2.124725	-0.130873
16	6	0	-3.349354	-0.109612	0.658762
17	6	0	-4.193173	-1.275338	1.080228
18	8	0	-4.048665	1.012149	0.420769
19	6	0	-5.314232	-1.647997	0.076969
20	6	0	-4.793042	-1.962433	-1.326058
21	8	0	-6.341580	-0.645114	0.040210
22	1	0	5.160947	-1.843807	2.608149
23	1	0	3.059406	0.057511	-2.319288
24	1	0	3.468352	1.147777	1.845369
25	1	0	5.041588	-2.600472	0.427097
26	1	0	4.721950	-3.006295	-1.826705
27	1	0	2.824159	2.838110	0.128740
28	1	0	2.655859	2.395351	-1.556705
29	1	0	0.696758	0.001096	0.206815
30	1	0	-0.598200	3.086943	-0.519226
31	1	0	-1.462208	-1.073551	0.715432
32	1	0	-3.549581	-2.144376	1.240643
33	1	0	-4.668878	-1.031529	2.038645
34	1	0	-3.376010	1.708743	0.141922
35	1	0	-5.810462	-2.536831	0.479605
36	1	0	-4.317306	-1.086541	-1.780948
37	1	0	-5.621564	-2.273900	-1.968707
38	1	0	-4.056840	-2.773058	-1.294817
39	1	0	-5.913313	0.211147	-0.118527

5P:

Center Number	Atomic Number	Atomic Type	Coordinates (Angstroms) X	Y	Z
1	8	0	-4.090819	-2.345228	-2.042402
2	6	0	-4.191199	-0.811672	1.252618
3	6	0	-3.575195	0.424716	1.020678
4	6	0	-3.116225	0.738539	-0.261844
5	6	0	-3.283452	-0.178057	-1.309887
6	6	0	-3.899902	-1.408193	-1.060450
7	6	0	-4.359821	-1.735824	0.219329
8	8	0	-4.619486	-1.064444	2.529875
9	6	0	-2.440777	2.067141	-0.524171
10	6	0	-0.938335	2.090056	-0.384471
11	6	0	-0.156273	1.045218	0.033997
12	8	0	-0.414837	3.276153	-0.712032
13	6	0	1.276255	1.169450	0.144303
14	6	0	2.063196	0.052844	0.600238
15	8	0	1.854087	2.283986	-0.162891
16	6	0	3.428553	0.129097	0.722979
17	6	0	4.288044	-1.002479	1.208672
18	8	0	4.097641	1.248845	0.428259
19	6	0	5.015471	-1.812604	0.114472
20	6	0	5.983929	-1.009919	-0.751746
21	8	0	3.996220	-2.440753	-0.683149
22	1	0	-3.730948	-2.026429	-2.882624
23	1	0	-3.460339	1.124228	1.842575
24	1	0	-2.935032	0.063902	-2.310543
25	1	0	-4.841373	-2.693866	0.390419
26	1	0	-5.027359	-1.940787	2.581572
27	1	0	-2.670178	2.417093	-1.538268
28	1	0	-2.835380	2.834366	0.154069
29	1	0	-0.623800	0.104329	0.290998
30	1	0	0.578043	3.187135	-0.580258
31	1	0	1.564477	-0.873542	0.855292
32	1	0	5.046166	-0.592798	1.885170
33	1	0	3.666696	-1.696976	1.780240
34	1	0	3.408196	1.914308	0.126123
35	1	0	5.580352	-2.591987	0.645402
36	1	0	5.463148	-0.232342	-1.317067
37	1	0	6.489937	-1.675487	-1.460100
38	1	0	6.751919	-0.534032	-0.132961
39	1	0	4.427419	-3.005487	-1.339624

5Q:

Center Number	Atomic Number	Atomic Type	Coordinates (Angstroms) X	Y	Z
1	8	0	-4.703211	-1.044119	2.504071
2	6	0	-3.900733	-1.457938	-1.017625
3	6	0	-3.271734	-0.243954	-1.320393
4	6	0	-3.111265	0.720321	-0.320900
5	6	0	-3.588720	0.473400	0.974172
6	6	0	-4.214882	-0.744446	1.258929
7	6	0	-4.377182	-1.719164	0.269017
8	8	0	-4.028473	-2.364590	-2.037556
9	6	0	-2.426762	2.032508	-0.636828
10	6	0	-0.929690	2.067132	-0.450846
11	6	0	-0.150469	1.025591	-0.019029
12	8	0	-0.406850	3.258531	-0.759733
13	6	0	1.278416	1.159029	0.123874
14	6	0	2.063644	0.044318	0.587122
15	8	0	1.854759	2.279959	-0.162368
16	6	0	3.426095	0.128074	0.735200
17	6	0	4.284494	-1.002885	1.224172
18	8	0	4.093177	1.254562	0.462540
19	6	0	5.027768	-1.802518	0.132723
20	6	0	6.000810	-0.988690	-0.717969
21	8	0	4.020390	-2.430911	-0.679475
22	1	0	-4.537514	-0.310110	3.112899
23	1	0	-2.914992	-0.067636	-2.330229
24	1	0	-3.473342	1.222432	1.753262
25	1	0	-4.871305	-2.655487	0.510445
26	1	0	-4.475998	-3.163647	-1.724349
27	1	0	-2.623117	2.322460	-1.676677
28	1	0	-2.842844	2.837504	-0.018019
29	1	0	-0.616827	0.079395	0.220033
30	1	0	0.583519	3.175108	-0.606264
31	1	0	1.566390	-0.887483	0.824637
32	1	0	5.033012	-0.594556	1.912036
33	1	0	3.659463	-1.703884	1.783665
34	1	0	3.405045	1.918229	0.153320
35	1	0	5.591339	-2.582115	0.664709
36	1	0	5.481530	-0.210359	-1.283655
37	1	0	6.518102	-1.646546	-1.425323
38	1	0	6.759494	-0.512501	-0.088011
39	1	0	4.460958	-2.992943	-1.332029
